# LncRNA WAC-AS1 expression in human tumors correlates with immune infiltration and affects prognosis

**DOI:** 10.1186/s41065-023-00290-z

**Published:** 2023-05-30

**Authors:** Yanyang Wang, Haiyan Gong, Yue Cao

**Affiliations:** 1 Department of Nuclear Medicine, Nanjing Drum Tower Hospital, Affiliated Hospital of Medical School, Nanjing University , Nanjing, 210008 China; 2 Medical Examination Center, Nanjing Drum Tower Hospital, Affiliated Hospital of Medical School, Nanjing University, Nanjing, 210008 China; 3 The Laboratory Center for Basic Medical Science, Nanjing Medical University, 101 Longmian Avenue, Jiangning District, Nanjing, Jiangsu 211166 China

**Keywords:** WAC-AS1, Pan-cancer, Prognosis, Immune cell infiltration, Chemotherapy resistance

## Abstract

**Background:**

WAC-antisense RNA1 (WAC-AS1) is a newly identified long non-coding RNA (lncRNA) implicated in the prognosis and development of a few types of tumors. However, the correlations of WAC-AS1 with immune infiltration and patient prognosis in pan-cancer remain unclear. In the present study, we aimed to investigate the prognostic value and immunological functions of WAC-AS1 across 33 different types of cancers.

**Methods:**

To investigate the potential oncogenic roles of WAC-AS1, bioinformatics analyses were performed using the Cancer Genome Atlas (TCGA) and Genotype Tissue-Expression (GTEx) datasets. The correlations of WAC-AS1 with prognosis, clinical phenotype, tumor mutational burden (TMB), microsatellite instability (MSI), tumor regulation-related genes, tumor microenvironment, immune cell infiltration, and drug resistance to commonly used chemotherapy drugs in different types of tumors were explored. Gene Set Enrichment Analysis (GSEA) and Gene Set Variation Analysis (GSVA) were performed to explore the biological functions of WAC-AS1 in tumors. In situ hybridization (ISH) was performed in tissue microarray (TMA) to confirm the expression of WAC-AS1 in multiple tumor tissues.

**Results:**

WAC-AS1 showed aberrant expression in most cancers when compared to the normal tissues. It also has prognostic value in multiple types of cancers. Elevated WAC-AS1 expression was associated with poor prognosis and overall survival in adrenocortical carcinoma (ACC), breast invasive carcinoma (BRCA), and liver hepatocellular carcinoma (LIHC). A significant negative correlation between WAC-AS1 expression and overall survival was observed in brain lower-grade glioma (LGG), pancreatic adenocarcinoma (PAAD), and skin cutaneous melanoma (SKCM). The expression of WAC-AS1 also showed a correlation with clinical stage in six types of tumors, and with tumor mutational burden and microsatellite instability in several different types of cancers. The immune scores of those cancers were found to be significant. Additionally, the effectiveness of fluorouracil and four other anticancer drugs was significantly different based on the expression of WAC-AS1 in these cancers. Moreover, the ISH results showed in six types of tumors, the expression of WAC-AS1 was consistent with the Pan-cancer analysis using TCGA and GTEx database.

**Conclusions:**

These results indicate an intensive involvement of WAC-AS1 in the regulation of immune responses, immune cell infiltration, and malignant properties in various types of cancers, suggesting that WAC-AS1 may serve as a prognostic marker across diverse types of cancers.

**Supplementary Information:**

The online version contains supplementary material available at 10.1186/s41065-023-00290-z.

## Introduction

Cancer is still a major cause of death and clearly affects quality of life worldwide. The World Health Organization (WHO) reports rapid increases in cancer incidence and mortality; however, newly developing therapies, especially immunotherapy that includes immune checkpoint blocking therapy, have reduced the mortality of some kinds of cancers, including melanoma and non-small cell lung cancer [1, 2]. The development and improvement of public databases, such as TCGA and GTEx, have increased the possibility of revealing novel targets with high potential for immunotherapy, while allowing establishment of their correlations with clinical phenotype and prognosis by analysis of pan-cancer gene expression. These targets can include long non-coding RNAs (lncRNAs).

The lncRNAs are a subclass of non-coding RNAs with a length of more than 200 nucleotides. They do not encode proteins, but they have been reported to participate in several tumor-related biological prosses [3]. Recent studies have demonstrated a tight association between abnormal expression of specific lncRNAs and tumor proliferation, invasion, and metastasis. Accumulating evidence indicates that lncRNAs play an essential role in regulating the tumor microenvironment, immune cell infiltration, and immune tolerance [4–7].

WAC-antisense RNA1(WAC-AS1), a novel lncRNA located in 10p12.1, is the antisense RNA head-to-head of WAC (WW domain-containing adaptor with coiled-coil). The study of this lncRNA is very limited; however, the existing evidence indicates that WAC-AS1 may take part in multiple biological processes in various tumors. WAC-AS1 was characterized as a ferroptosis-related lncRNA that served as a protective factor for survival in glioma [8]. Expression of WAC-AS1, together with another 7 genes, was also significantly correlated with the survival of patients with ovarian cancer [9]. By contrast, in hepatocellular carcinoma, WAC-AS1 regulated the glycolysis gene ARPP19 while functioning as a competing endogenous RNA (ceRNA) that inhibited the targeting of ARPP19 by miR-320d, thereby promoting glycolysis and tumor progression [10]. All this evidence suggests that WAC-AS1 might play an important role in multiple tumor types and that its abnormal expression could affect patient prognosis.

Nevertheless, studies on WAC-AS1 are still limited, as recent investigations have tended to focus on a specific type of cancer. No pan-cancer studies have explored the relationship between WAC-AS1 and various cancers; therefore, in the present study, we searched multiple databases, including TCGA and GTEx, to evaluate the expression levels of WAC-AS1 and their association with clinical presentations and prognoses in different tumor settings. We also explored the potential correlations between WAC-AS1 expression and MSI, TMB, the tumor microenvironment, and immune infiltration levels in thirty-three different types of cancer. In addition, we performed synergistic expression analysis of genes involved in autophagy, ferroptosis, pyroptosis, and hypoxia. We conducted GSVA and GSEA analysis to evaluate the biological function of WAC-AS1 in cancer. We also used the Cellminer database to investigate the association between drug resistance and WAC-AS1 expression. ISH was also done in TMA to confirm the expression of WAC-AS1 in multiple tumor tissues.

Our findings suggest that WAC-AS1 can be a useful prognostic marker in a wide range of cancers and that WAC-AS1 plays a critical role in the tumor microenvironment and tumor immunity through its effects on tumor-infiltrating immune cells, TMB, and MSI. Moreover, WAC-AS1 was associated with sensitivity to five chemotherapy drugs. The current study provides novel perspectives regarding the functional role of WAC-AS1 in a variety of cancers.

## Methods

### Data collection and analysis

We used the University of California Santa Cruz (UCSC) Xena functional genomics explorer (https://xenabrowser.net/) to download original high-throughput sequencing data [11], including mRNA and lncRNA expression data and single nucleotide polymorphisma (SNP) data, for 33 tumor types from the TCGA database (https://portal.gdc.cancer.gov/) [12] for subsequent analysis. We also downloaded gene expression data for different tissues from the Genotype Tissue-Expression (GTEx) database (https://commonfund.nih.gov/GTEx) [13]. The fragments per kilobase million (FPKM) values data were downloaded from TCGA and GTEx database and merged according to the common genes. Use the normalizeBetweenArrays function to correct batch effects so that to make the gene expression of two data sets at the same level. Then the (FPKM + 1) format RNA-seq data were Log2 transformed for the following analysis. Data were analyzed using R software Version 4.0.2 (https://www.R-project.org) with the R package “ggpubr”. Box plots were generated using the R package “ggplot2”.

### Analysis of the correlations of WAC-AS1 with prognosis and clinical phenotype

The TCGA database was used to retrieve the survival and clinical phenotype data. The indicator of overall survival (OS) was employed to examine the correlation between WAC-AS1 expression and patient prognosis. Survival (*P* < 0.05) was analyzed for each cancer type using the Kaplan–Meier analysis (log-rank test). The R packages “survival” and “survminer” were adopted to estimate the survival curves. The possibility that WAC-AS1 expression influences survival at a pan-cancer level was addressed by performing Cox analysis with the “survival” and “forestplot” packages in the R language.

### Relationship between WAC-AS1 expression and immunity

The tumor purity, immune, and stromal scores were calculated for each tumor sample using the ESTIMATE algorithm [14]. The degree of immune infiltration was calculated using the R software package “estimate” to test the correlations between WAC-AS1 expression and these scores.

We also used the CIBERSORT, which is a method to characterize cell composition from gene expression profiles and is the most commonly cited tool for estimating and analyzing immune infiltrating cells. RNA-seq data from 33 cancers patients from different subgroups were analyzed using CIBERSORT algorithm to infer relative proportions of 22 kinds of immune infiltrating cells and to conduct correlation analysis between WAC-AS1 expression levels and immune infiltrating cells content [15]. The Spearman test was used to evaluate the correlation between WAC-AS1 expression and the immune cell infiltration level in each cancer (*P* < 0.05 as significant).

The co-expression of WAC-AS1 and immune-related genes was also analyzed using data from the TISIDB database (http://cis.hku.hk/TISIDB/index.php) [16]. Specifically, genes related to the major histocompatibility complex (MHC), immune stimulator, immune-inhibitor, immune checkpoint, chemokine, and chemokine receptor proteins were analyzed with the R-package “ggplot2”.

### Correlation of WAC-AS1 expression with tumor mutation burden, tumor microsatellite instability, and tumor regulation-related gene expression

For each tumor sample, we calculated the variable frequency and variation number or exon length. The nonsynonymous mutation site was divided by the size of the coding sequence to determine the TMB. The value of the tumor MSI for each patient (retrieved from TCGA) was derived from the following study: the landscape of microsatellite instability across 39 cancer types [17].

We determined the association between WAC-AS1 expression and TMB or MSI using Spearman’s rank correlation coefficient analysis. Radar maps, generated by the “Fmsb” package in the R language, were used to present the results.

We used the expression profile data retrieved from TCGA to explore the expression of tumor-regulated genes, including those associated with hypoxia, autophagy, pyroptosis, and ferroptosis, in different types of cancer and to assess the association between the expression of those genes and WAC-AS1 expression. Heat maps were generated using the R-package “ggplot2.”

### Gene set variation analysis and gene set enrichment analysis

GSVA implements a statistical method to study gene set enrichment. It comprehensively scores the targeted gene set, converts the alterations of gene expression into related pathway-level changes, and then identifies the biological function of the sample. In order to reduce interference with redundant information in the pathway, we removed duplicate genes from each gene set. Therefore, the genes which appears in two or more pathways were removed. We employed the molecular signatures database (v7.0 version) http://www.broadinstitute.org/msigdb [18] to retrieve the gene sets (50 hallmark pathways) and then scored each set of genes using the R (“GSVA” package) to trace the possibly altered biological functions in each sample.

GSEA is used to interpret the distinguished gene expression profiles of two different samples and then to identify the general expression trends of the preset set. In the current study, we used the “cluster profiler” and “enrich blot” packages for GSEA analysis. We compared the pathways distinguished between the high and low WAC-AS1 expression groups to explore the possible molecular mechanism of prognosis differences in 33 types of tumors from different patients and showed 3 types of tumors in the results.

### Prediction of chemosensitivity

The Cellminer database is built upon 60 human cancer cell lines collected by the Cancer Research Center of the National Cancer Institute (NCI) [19]. We used the NCI-60 cell line, which is the cancer cell line generally used for anticancer drug evaluation, to retrieve drug sensitivity data and RNA sequencing data to analyze the correlation between specific genes and the sensitivity of commonly used antitumor drugs (*P* less than 0.05 was considered statistically significant).

### In situ hybridization (ISH)

The multi-tumor tissue microarray (TMA) block (20 types of tumors, 61 tumors per block, 2 cores of 1.5 mm per tumor, Cat No.ZL-MTU122) were constructed by Shanghai Wellbio Biotechnology Co., Ltd (Wellbio Biotechnology Co., Shanghai, China). The expression of WAC-AS1 was detected using ISH Test Kit (Boster, Wuhan, China) according to the manufacturer’s protocols. Three-phase 30-base oligonucleotide probes labeled with DIG-dUTP at the 3’ end were purchased from Boster (Wuhan, China). The sequences used for the probes are:

1. 5’-GGAGAATCTGATTTCCAGAGTTACCACATTATAATACTAT-3’,
2. 5’-TAGAAATTCTGGAGTTGAAAAGTATTATAACTAAAACTGA-3’,
3. 5’-CAGCTTTGATGAAATACATGAATCTAGATATTCAAGAGGC-3’.

### Assessment of in situ hybridization

Using the therhold analysis module and the HDAB-DAB filters in visiopharm software, the region of interest (ROI) was segmented based on the intensity of staining, and corresponding labels are scored (strong positive: 0–75; moderate positive: 76–120; weakly positive: 121–160; negative: 161–212). And exported the corresponding area in the corresponding area (μm2). Histochemistry score (H-score), is a histological scoring method that converting the number of positive cells and their staining intensity in each slice into corresponding numerical values. H-Score = ∑(pi × i) = (percentage of weak intensity × 1) + (percentage of moderate intensity × 2) + (percentage of strong intensity × 3). The H-score is between 0 and 300, and the larger the data, the stronger the comprehensive positive intensity.

### Statistical analysis

The R language (version 4.0) was employed for all statistical analyses. We calculated the hazard ratios (HRs) and 95% confidence intervals using univariate survival analysis. The survival of patients with different WAC-AS1 expression levels (high and low groups) was compared using Kaplan–Meier analysis. The statistical tests used in all analyses were bilateral. *P* < 0.05 was considered statistically significant.

## Results

### Differential expression of WAC-AS1 in human tumors and normal tissues

**Fig. 1:**
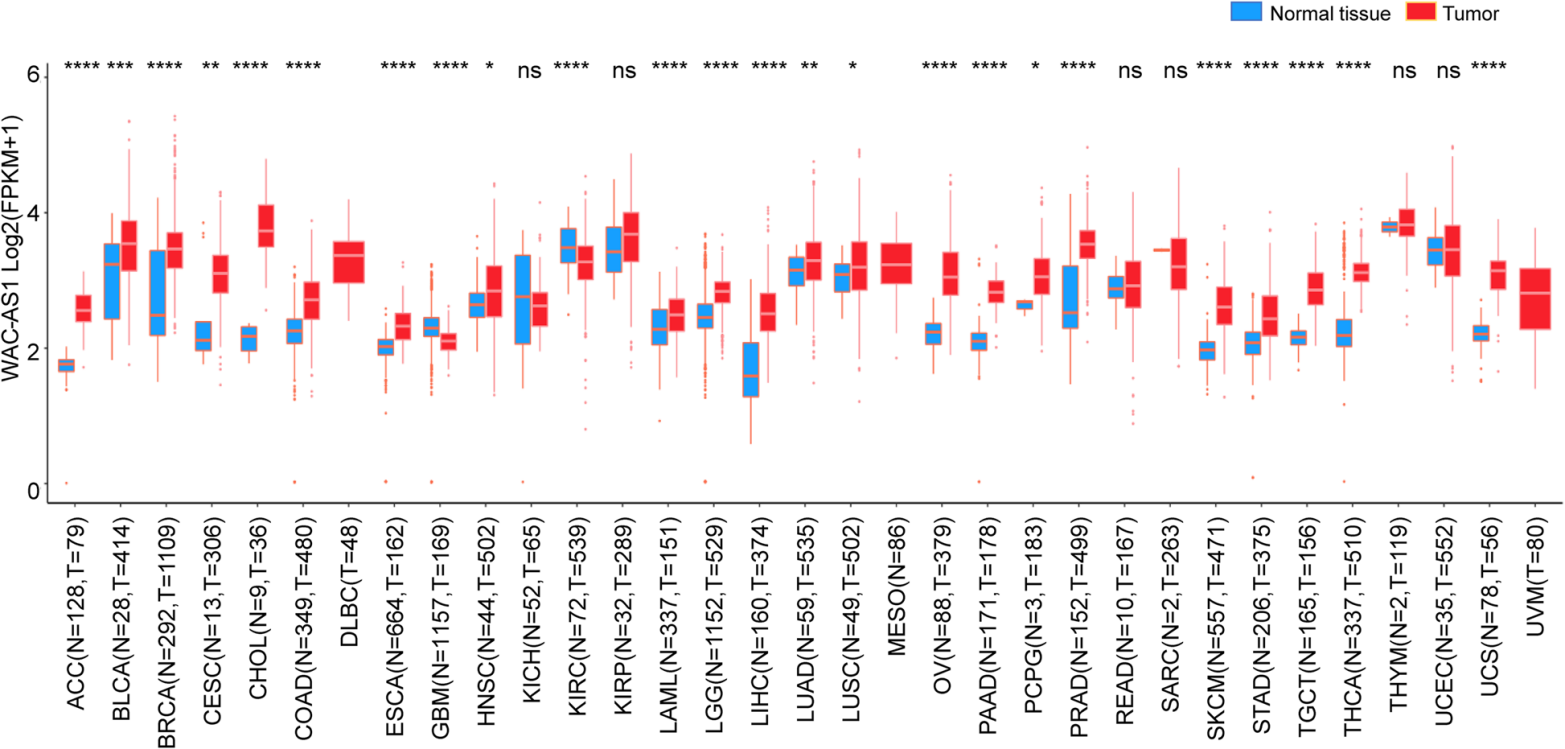
Expression levels of lncRNA WAC-AS1 in 33 different types of cancer using the TCGA and GTEx databases. “*” indicates *P* value smaller than 0.05, “**”indicates *P* value smaller than 0.01, “***” indicates *P* value smaller than 0.001, and “****” indicate *P* values smaller than 0.0001, “ns” means no significance

We first analyzed the expression of WAC-AS1 in 33 types of human cancers using information retrieved from the TCGA and GTEx pan-cancer databases. No normal tissue data were available for lymphoid neoplasm diffuse large B-cell lymphoma (DLBC), mesothelioma (MESO), and uveal melanoma (UVM), but we found significantly different WAC-AS1 expression in 24 tumor types. Of those 24 cancers, WAC-AS1 was expressed more strongly in 22 tumors (adrenocortical carcinoma (ACC), bladder urothelial carcinoma (BLCA), breast invasive carcinoma (BRCA), cervical squamous cell carcinoma and endocervical adenocarcinoma (CESC), cholangiocarcinoma (CHOL), colon adenocarcinoma (COAD), esophageal carcinoma (ESCA), head and neck squamous cell carcinoma (HNSC), acute myeloid leukemia (LAML), brain lower grade glioma (LGG), liver hepatocellular carcinoma (LIHC), lung adenocarcinoma (LUAD), lung squamous cell carcinoma (LUSC), ovarian serous cystadenocarcinoma (OV), pancreatic adenocarcinoma (PAAD), pheochromocytoma and paraganglioma (PCPG), prostate adenocarcinoma (PRAD), skin cutaneous melanoma (SKCM), stomach adenocarcinoma (STAD), testicular germ cell tumor (TGCT), thyroid carcinoma (THCA), and uterine carcinosarcoma (UCS)) when compared with the normal tissues. In contrast, WAC-AS1 expression was lower in glioblastoma multiforme (GBM) and kidney renal clear cell carcinoma (KIRC) tumors than in the normal tissues and was similar in kidney chromophobe (KICH), kidney renal papillary cell carcinoma (KIRP), rectum adenocarcinoma (READ), sarcoma (SARC), thymoma (THYM), and uterine corpus endometrial carcinoma (UCEC) tumor tissues and in normal tissues (Fig. 1).

### Association between WAC-AS1 expression and cancer patient prognosis

**Fig. 2:**
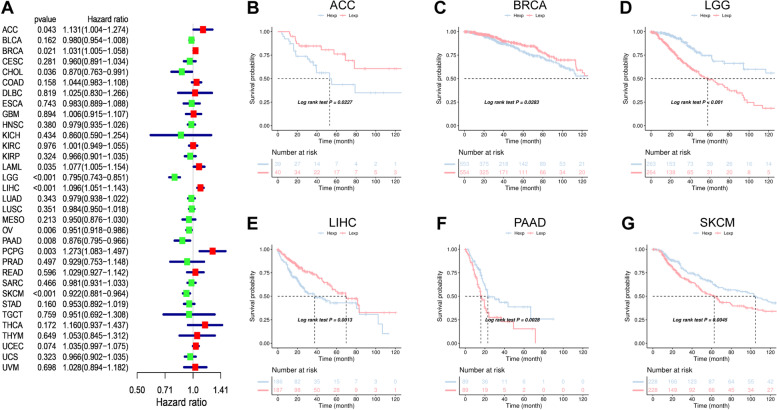
Association between WAC-AS1 expression and overall survival (OS). **A** Forest plot of OS in 33 types of tumors. **B**–**G** Kaplan-Meier analysis of the association between WAC-AS1 expression and OS

We assessed the efficacy of the WAC-AS1 expression level as a predictor of patient prognosis by conducting a survival association analysis in the TCGA cohort for each cancer. As shown in Fig. 2A, the WAC-AS1 expression levels were associated with overall survival in ACC (*P* = 0.043), BRCA (*P* = 0.021), CHOL (*P* = 0.036), LAML (*P* = 0.035), LGG (*P* < 0.001), LIHC (*P* < 0.001), OV (*P* = 0.006), PAAD (*P* = 0.008), PCPG (*P* = 0.003), and SKCM (*P* < 0.001). Furthermore, in ACC, BRCA, LAML, LIHC, and PCPG, WAC-AS1 was a high-risk gene, whereas it may be a low-risk gene in CHOL, LGG, OV, PAAD, and SKCM.

Kaplan–Meier survival analysis revealed an association between high WAC-AS1 expression and poor prognosis in patients with ACC (*P* = 0.0227), BRCA (*P* = 0.0283), and LIHC (*P* = 0.0013), indicating that WAC-AS1 has oncogenic potential in these cancer types. By contrast, higher levels of WAC-AS1 predicted longer overall survival times in patients with LGG (*P* < 0.001), PAAD (*P* = 0.0028), and SKCM (*P* = 0.0045) (Fig. 2B-G).

### Association between WAC-AS1 levels and clinical cancer phenotypes

**Fig. 3:**
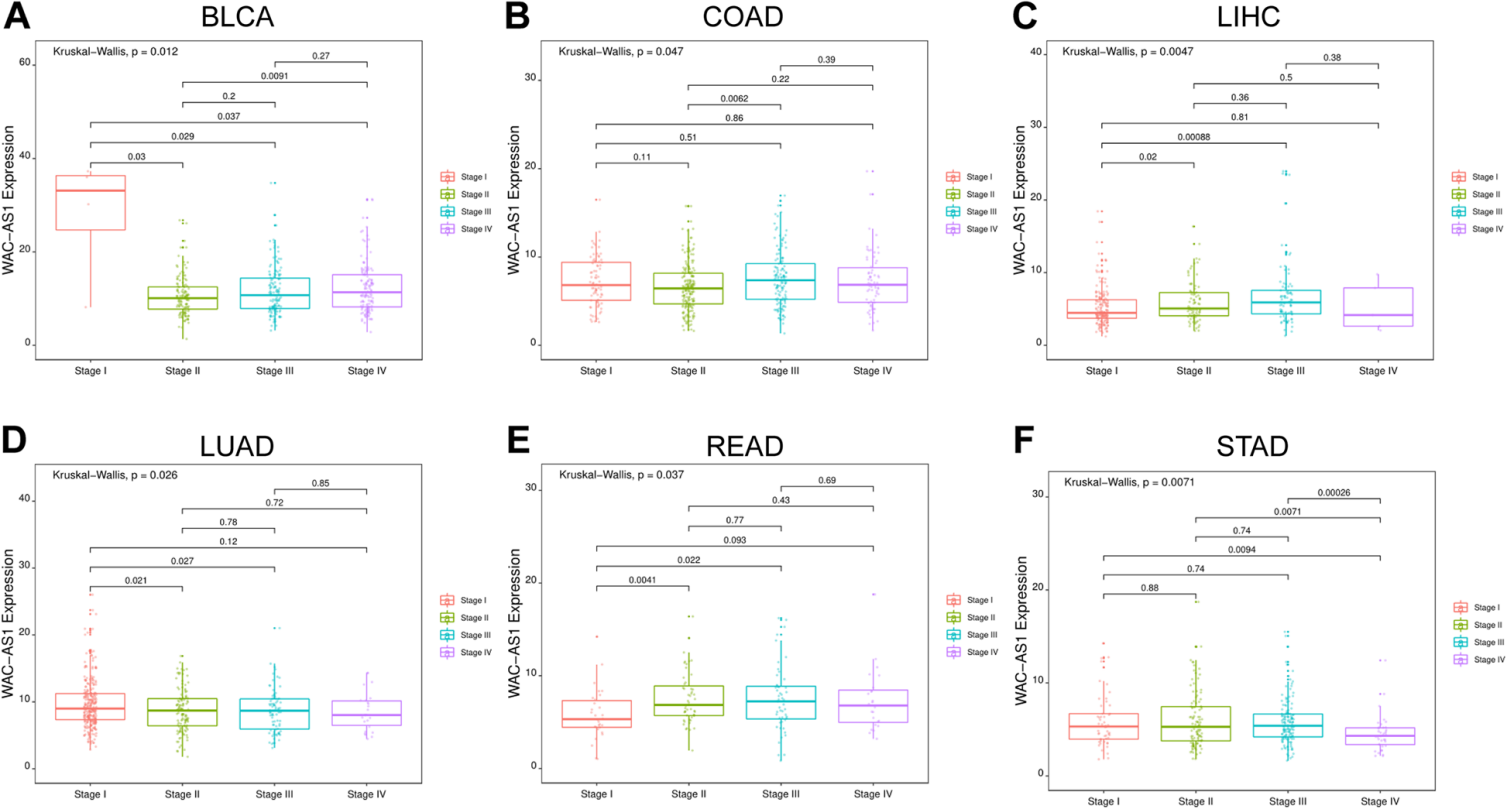
Association between WAC-AS1 expression and tumor stage in **A** Bladder urothelial carcinoma (BLCA), **B** Colon adenocarcinoma (COAD), **C** Liver hepatocellular carcinoma (LIHC), **D** Lung adenocarcinoma (LUAD), **E** Rectum adenocarcinoma (READ), and **F** Stomach adenocarcinoma (STAD)

We also explored the relevance of WAC-AS1 expression to tumor clinical stages, tumor grades and the patients ages. WAC-AS1 expression was associated with tumor grades and patients ages in some kinds of tumors (Supplementary Figs. 1 and 2). We observed a strong association between WAC-AS1 expression and the staging of BLCA, COAD, LIHC, LUAD, READ, and STAD. The most significant differences in WAC-AS1 expression were obtained for stage I and stage II cancers, except for COAD and STAD (Fig. 3). As shown in Fig. 3, the expression of WAC-AS1 was lower for BLCA (Fig. 3A; *P* = 0.03) and LUAD (Fig. 3D; *P* = 0.021) in stage II than in stage I, but was higher in stage II than in stage I LIHC (Fig. 3C; *P* = 0.02) and READ (Fig. 3E; *P* = 0.0041). In COAD, a significantly elevated expression of WAC-AS1 was observed only from stage II to stage III (Fig. 3B; *P* = 0.0062). STAD showed no statistically significant differences in WAC-AS1 levels in stages I, II, and III, but the level was markedly decreased in stage IV compared to stage III, stage II, and stage I (*P* = 0.00026, *P* = 0.0071, and *P* = 0.0094, respectively) (Fig. 3F).

### Relationship between WAC-AS1 expression and the tumor microenvironment

**Fig. 4:**
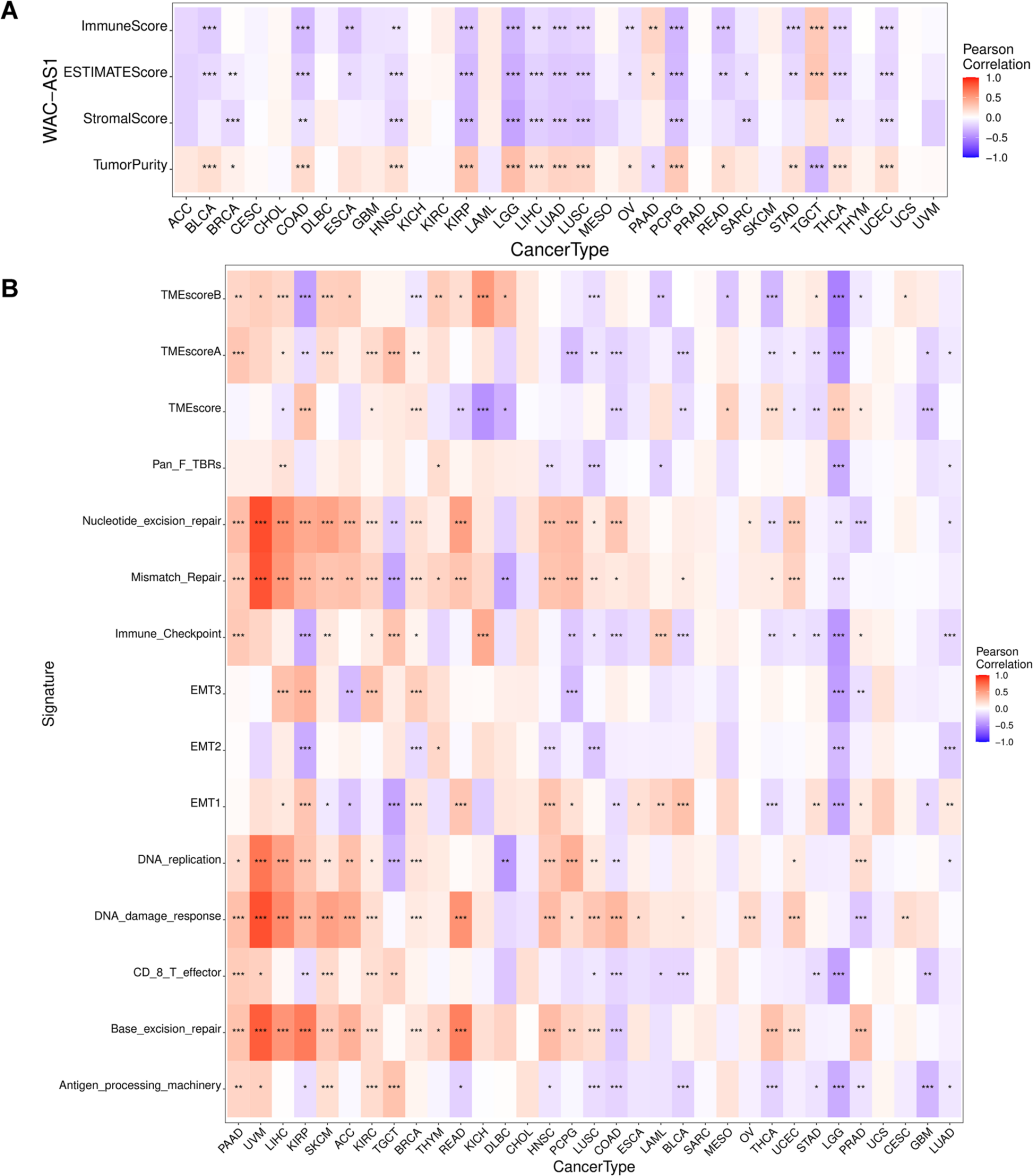
Analysis of WAC-AS1 expression with tumor microenvironment in 33 cancer types. **A** Association of WAC-AS1 with tumor microenvironment scores. **B** Association of WAC-AS1 with tumor microenvironment-related biological processes. “*” indicates *P* value smaller than 0.05, “**”indicates *P* value smaller than 0.01, “***” indicates *P* value smaller than 0.001

The tumor microenvironment, or tumor stroma, is a complex network of cells (immune cells, endothelial cells, fibroblasts, cancer cells, etc.), extracellular matrix, growth factors, and chemicals, and it inevitably impacts tumor diagnosis, survival outcome, and drug resistance [20]. We studied the relationship between WAC-AS1 and the tumor microenvironment using the ESTIMATE algorithm to compare the tumor purity, stromal score, and immune score in the 33 cancer types. As shown in Fig. 4A, WAC-AS1 expression was related to the tumor microenvironment in 19 cancers (Supplementary Table 1) and was significantly negatively correlated with the immune score as well as the stromal score, while positively related to tumor purity, in KIRP, LGG, and PCPG. By contrast, in PAAD and TGCT, WAC-AS1 levels were positively correlated with immune cell infiltration and negatively associated with tumor purity (Fig. 4A, Table 1).

**Table 1:** Correlation of WAC-AS1 expression with tumor microenvironment scores by TIMER

	KIRP (*P*-Value/COR)	LGG (*P*-Value/COR)	PCPG (*P*-Value/COR)	PAAD (*P*-Value/COR)	TGCT (*P*-Value/COR)
StromalScore	***/-0.31	***/-0.39	***/-0.26	0.08	0.15
ImmuneScore	***/-0.26	***/-0.31	***/-0.31	**/0.21	***/0.28
ESTIMATEScore	***/-0.30	***/-0.35	***/-0.30	*/0.15	***/0.29
TumorPurity	***/0.30	***/0.33	***/0.27	*/-0.16	***/-0.30

*KIRP* Kidney renal papillary cell carcinoma, *LGG* Brain Lower Grade Glioma, *PCPG* Pheochromocytoma and Paraganglioma, *PAAD* Pancreatic adenocarcinoma, *TGCT* Testicular Germ Cell Tumors

“*” indicates *P* value smaller than 0.05, “**”indicates *P* value smaller than 0.01, “***” indicates *P* value smaller than 0.001

Correlation analysis of the relationship between the tumor microenvironment-based gene signature [21] and WAC-AS1 expression levels (Supplementary Fig. 3) revealed the strongest relationship for KIRP (*n* = 14), BRCA (*n* = 12), LUSC (*n* = 12), and LGG (*n* = 12) (Fig. 4B). WAC-AS1 expression levels were positively related to the scores for Mismatch Repair, DNA replication, and Nucleotide excision repair in KIRP, BRCA, and LUSC, whereas WAC-AS1 expression was negatively correlated with Mismatch Repair and DNA replication scores in LGG. Moreover, among these 4 cancer types, WAC-AS1 expression levels had an opposite association with the scores of TME score B and EMT2. These data indicate that WAC-AS1 may have a critical role in the tumor microenvironment and the formation of multiple tumor-related components in those 4 tumor types.

### Relationship between WAC-AS1 levels and tumor immune cell infiltration

**Fig. 5:**
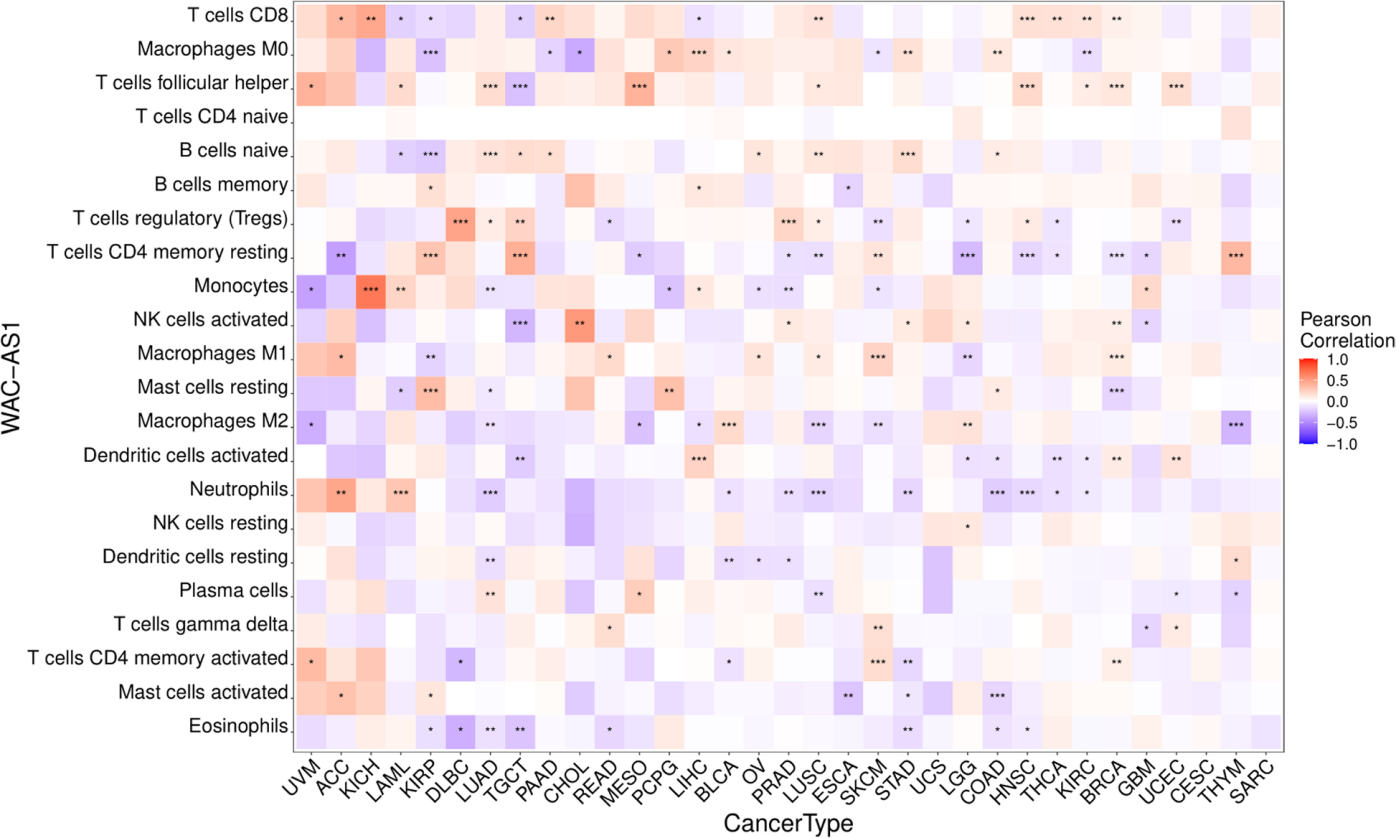
Relationship between WAC-AS1 expression and tumor infiltration of different immune cells in 33 types of tumors. “*” indicates *P* value smaller than 0.05, “**”indicates *P* value smaller than 0.01, “***” indicates *P* value smaller than 0.001

The levels of immune cell infiltration were clearly related to WAC-AS1 expression in most of the cancers, except for UCS, CESC, and SARC (Supplementary Table 2). WAC-AS1 expression levels were significantly related to CD8 T lymphocytes in 12 tumor types, M0 macrophage cells in 10 tumor types, and follicular helper T cells in 10 tumor types (Fig. 5).

WAC-AS1 expression had the most significant correlation with immune cell infiltration in the following tumors: BRCA (*n* = 8), KIRP (*n* = 9), LUAD (*n* = 10), LUSC (*n* = 9), SKCM (*n* = 8), and TGCT (*n* = 8) (Table 2).

**Table 2:** Correlation of WAC-AS1 expression with immune cell infiltration in different types of cancer

Cell Type	BRCA (*P*-Value/Cor)	KIRP (*P*-Value/Cor)	LUAD (*P*-Value/Cor)	LUSC (*P*-Value/Cor)	SKCM (*P*-Value/Cor)	TGCT (*P*-Value/Cor)
B cells naive	0.05	***/-0.22	***/0.14	**/0.13	0.09	*/0.18
B cells memory	0.04	*/0.16	-0.02	0.00	0.07	-0.01
Plasma cells	-0.02	-0.05	**/0.14	**/-0.14	0.02	-0.03
T cells CD8	**/0.08	*/-0.15	0.08	**/0.14	0.00	*/-0.20
T cells CD4 naive	0.00	0.00	0.00	-0.04	0.00	0.00
T cells CD4 memory resting	***/-0.11	***/0.30	-0.04	**/-0.14	**/0.14	***/0.40
T cells CD4 memory activated	**/0.09	-0.12	-0.06	-0.01	***/0.18	0.03
T cells follicular helper	***/0.13	-0.03	***/0.18	*/0.11	0.00	***/-0.27
T cells regulatory (Tregs)	-0.01	-0.08	*/0.09	*/0.09	**/-0.14	**/0.22
T cells gamma delta	-0.01	-0.12	-0.08	-0.05	**/0.16	0.08
NK cells resting	0.00	0.03	0.03	-0.01	-0.09	-0.13
NK cells activated	**/0.08	0.03	0.00	0.08	-0.04	***/-0.31
Monocytes	-0.03	0.08	**/-0.12	0.01	*/-0.11	-0.11
Macrophages M0	0.05	***/-0.26	0.08	0.08	*/-0.11	0.06
Macrophages M1	***/0.11	**/-0.19	0.04	*/0.10	***/0.23	-0.08
Macrophages M2	-0.05	-0.08	**/-0.12	***/-0.17	**/-0.14	-0.15
Dendritic cells resting	-0.05	0.05	**/-0.14	-0.04	-0.03	0.08
Dendritic cells activated	**/0.10	0.10	0.02	0.05	-0.01	**/-0.22
Mast cells resting	***/-0.18	***/0.34	*/-0.10	0.05	-0.02	0.02
Mast cells activated	0.03	*/0.13	-0.01	-0.06	-0.02	0.00
Eosinophils	-0.03	*/-0.14	**/-0.13	-0.02	-0.08	**/-0.25
Neutrophils	-0.04	-0.01	***/-0.21	***/-0.19	-0.01	-0.07

*BRCA* Breast invasive carcinoma, *KIRP* Kidney renal papillary cell carcinoma, *LUAD* Lung adenocarcinoma, *LUSC* Lung squamous cell carcinoma, *SKCM* Skin Cutaneous Melanoma, *TGCT* Testicular Germ Cell Tumors

“*” indicates *P* value smaller than 0.05, “**”indicates *P* value smaller than 0.01, “***” indicates *P* value smaller than 0.001

WAC-AS1 expression had a positive correlation with infiltration of naïve B lymphocytes in LUAD, LUSC, and TGCT, but a negative correlation in KIRP. Elevated WAC-AS1 expression was also positively correlated with increased infiltration of CD8 T lymphocytes in BRCA and LUSC, but was negatively correlated in KIRP and TGCT. WAC-AS1 expression was negatively correlated with infiltration of resting memory CD4 T lymphocytes in BRCA and LUSC, and positively correlated in KIRP, SKCM, and TGCT. WAC-AS1 expression levels were significantly positively correlated with follicular helper T cell infiltration in BRCA, LUAD, and LUSC but negatively correlated in TGCT.

WAC-AS1 expression had a positive correlation with M1 macrophage infiltration in BRCA, LUSC, and SKCM, but a negative correlation with KIRP. Infiltration of naïve CD4 T lymphocytes and resting NK cells had no correlation with or only slight differences in WAC-AS1 expression levels in any type of cancer, which could be attributed to differences in immune cell infiltration in different cancer types. Overall, WAC-AS1 appeared to affect immune cells to some extent and to participate in immune cell–oncological interactions.

### Relationship between WAC-AS1 expression and immune-related genes

**Fig. 6:**
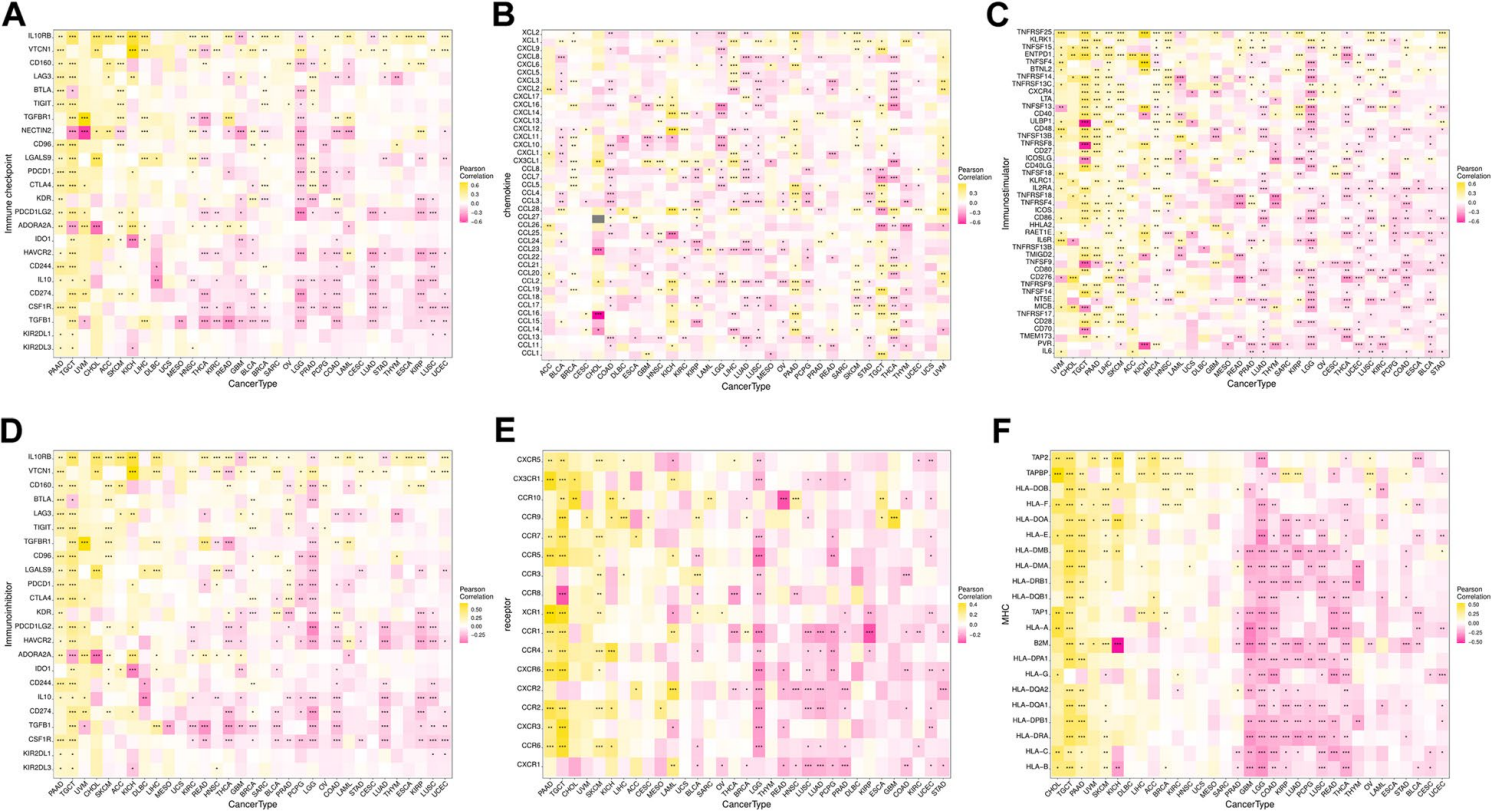
Co-expression of WAC-AS1 and immune-related genes. **A** Correlation between WAC-AS1 and immune checkpoints. **B** Correlation between WAC-AS1 and chemokines. **C** Correlation between WAC-AS1 and immunostimulatory genes. **D** Correlation between WAC-AS1 and immunoinhibitory genes. **E** Correlation between WAC-AS1 and chemokines receptors. **F** Correlation between WAC-AS1 and MHC genes. “*” indicates *P* value smaller than 0.05, “**”indicates* P* value smaller than 0.01, “***” indicates *P* value smaller than 0.001

Analysis of gene co-expression based on the TISIDB database revealed genes that encoded MHC, immune-stimulator, immune-inhibitor, chemokine, and chemokine receptor proteins, as well as immune checkpoint proteins. Almost all analyzed genes were strongly correlated with WAC-AS1 in all types of tumors except UCS, MESO, and SARC (Fig. 6).

### Relationship between WAC-AS1 expression levels and the TMB and MSI

**Fig. 7:**
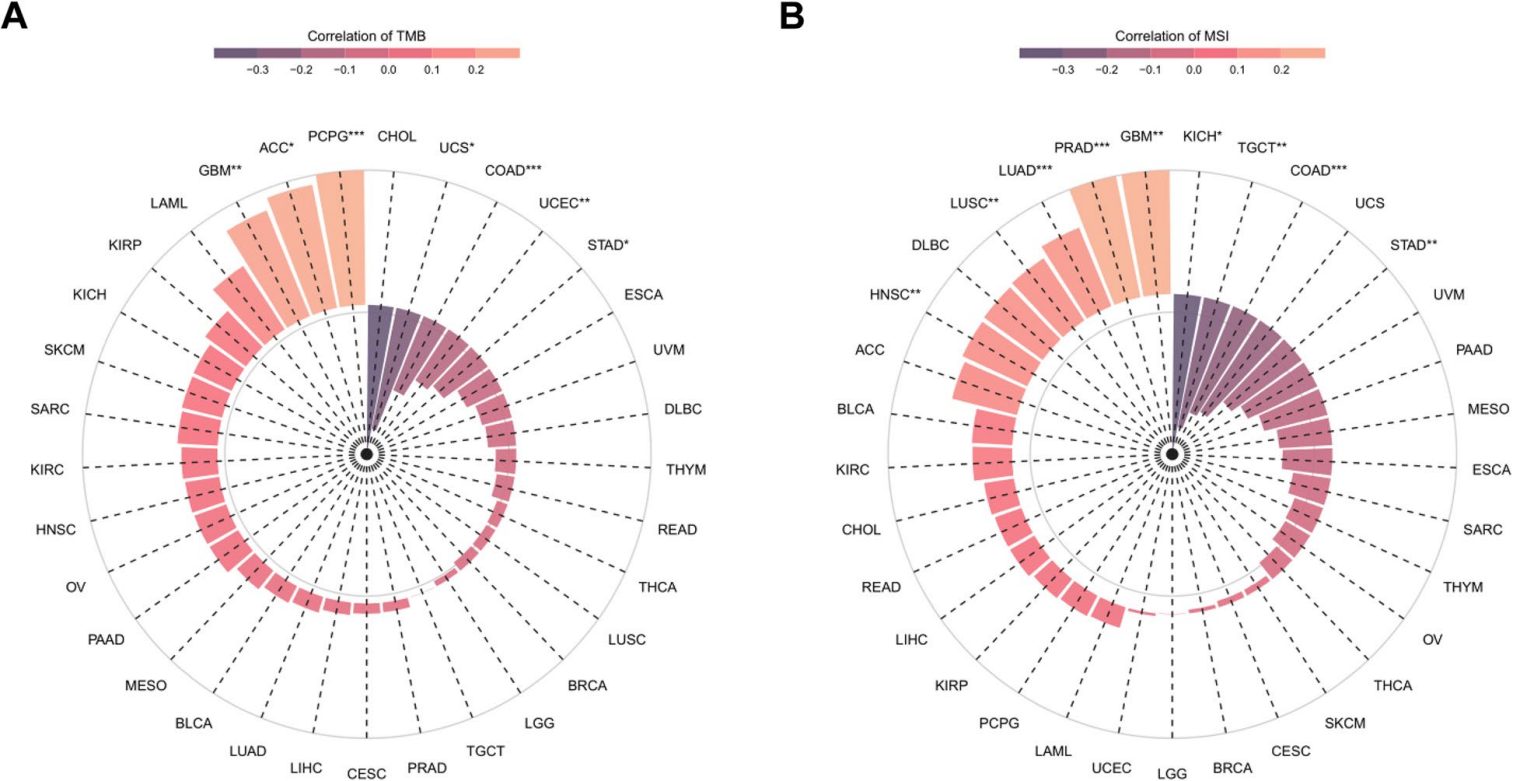
Correlation of WAC-AS1 expression with tumor mutational burden (TMB), microsatellite instability (MSI). **A** The correlations of WAC-AS1 expression and TMB in cancers. **B** The correlations of WAC-AS1 and MSI in cancers. “*” indicates *P* value smaller than 0.05, “**”indicates* P* value smaller than 0.01, “***” indicates *P* value smaller than 0.001

Both TMB and MSI affect the sensitivity to immune checkpoint blockades (ICBs) to modulate tumor initiation, and they can both serve as independent efficacy predictors for ICBs [22, 23]. WAC-AS1 expression was positively correlated with TMB in PGCG, ACC, and GBM, but negatively correlated with TMB in UCS, COAD, UCEC, and STAD (Fig. 7A). In general, a higher MSI in GBM, PRAD, LUAD, LUSC, and HNSC was associated with higher WAC-AS1 expression, while an opposite trend was noted for KICH, TGCT, COAD, and STAD (Fig. 7B).

### Relationship between WAC-AS1 expression levels and tumor-regulation related genes

WAC-AS1 is a ferroptosis-related lncRNA in ovarian cancer; therefore, we explored the relationship between WAC-AS1 expression and tumor regulation-related genes involved in hypoxia, autophagy, and pyroptosis, as well as ferroptosis.

**Fig. 8:**
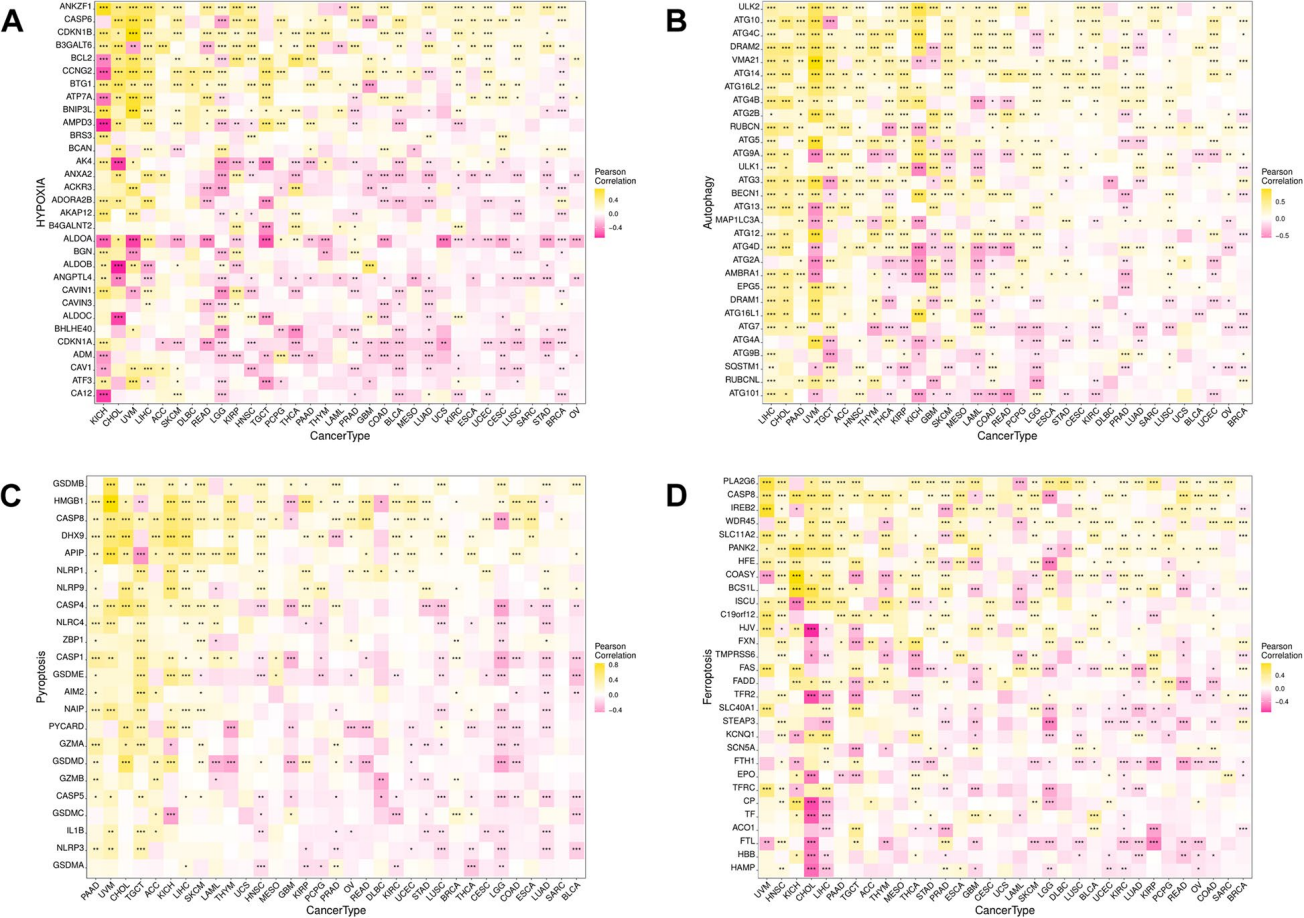
Co-expression of WAC-AS1 and tumor regulatory genes. **A** Correlation between WAC-AS1 and hypoxia genes. **B** Correlation between WAC-AS1 and antophagy genes. **C** Correlation between WAC-AS1 and pyroptosis genes. **D** Correlation between WAC-AS1 and ferroptosis genes. “*” indicates *P* value smaller than 0.05, “**”indicates *P* value smaller than 0.01, “***” indicates *P* value smaller than 0.001

Significant correlations were noted between WAC-AS1 and tumor regulation-related genes in all types of cancers, except for UCS, SARC, and MESO. Moreover, in UVM, the correlation coefficients between WAC-AS1 expression levels and the tumor regulation-related genes were all greater than 0.5, indicating that WAC-AS1 might be essential in the process of tumor regulation (Fig. 8).

### Gene set variation analysis and gene set enrichment analysis

Analysis of overall survival (OS), tumor microenvironment, and mutations revealed important but conflicting roles of WAC-AS1 in different cancers. WAC-AS1 is an important risk factor for LIHC, whereas it has a protective role on LGG (Fig. 2A, D-E); therefore, LIHC and LGG were selected as representative cancers for follow-up analysis. In addition, since WAC-AS1 did not appear to have an impact on SARC prognosis, SARC was adopted as a control. The top 10 genes associated with WAC-AS1 were identified in these three tumors using heat maps based on co-expression analysis (Supplementary Fig. 4A-C).

**Fig. 9:**
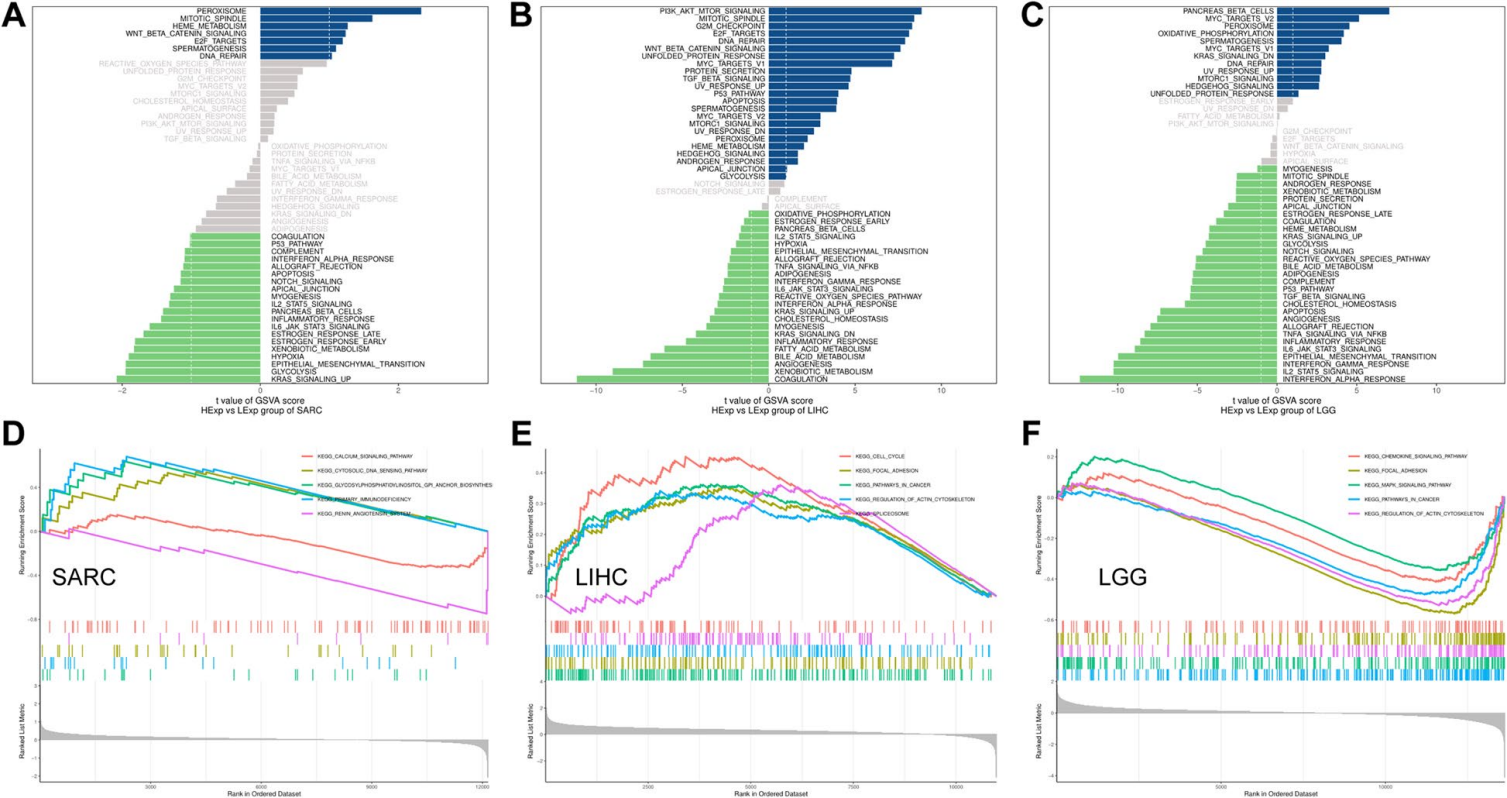
Significant WAC-AS1-related biological pathways analyzed by GSEA and GSVA. **A**-**C** Significant WAC-AS1-related biological pathways in SARC (**A**), LIHC (**B**), LGG (**C**) obtained by GSVA. **D**-**F** Significant WAC-AS1-related top five oncological signatures in SARC (**D**), LIHC (**E**), LGG (**F**) identified by GSEA analysis. Curves of different colors represents different functions or pathways regulated in different cancers. Peaks of upward curve represent positive regulation, while peaks of downward curve represent negative regulation

The possible mechanisms of action of WAC-AS1 in LIHC, LGG, and SARC were analyzed using GSVA and GSEA. GSVA analysis showed that, in SARC, the peroxisome, mitotic spindle, and heme metabolic pathways were the most upregulated in the WAC-AS1-positive expression group. By contrast, the most involved pathways negatively related to WAC-AS1 expression in SARC were KRAS signaling, glycolysis, and epithelial-mesenchymal transition (Fig. 9A). By contrast, in LIHC, mitotic spindle, TGF beta signaling, P53 pathway, apoptosis, heme metabolism, androgen response, apical junction, and glycolysis were significantly positively related to WAC-AS1, whereas the opposite relationship was seen in LGG. Pancreas beta cells, oxidative phosphorylation, and KARS signaling in DN were significantly negatively linked to WAC-AS1 in LIHC, but these pathways were positively linked in LGG (Fig. 9B, C).

The GSEA results showed that the calcium signaling pathway, and the renin-angiotensin system were negatively related to WAC-AS1, and the cytosolic DNA sensing pathway, glycosylphosphatidylinositol GPI anchor biosynthesis, and primary immunodeficiency were positively linked to WAC-AS1 in SARC (Fig. 9D). In LIHC, the GSEA results showed that the cell cycle, focal adhesion, pathways in cancer, regulation of actin cytoskeleton, and spliceosome were upregulated in the WAC-AS1-positive expression group. In LGG, chemokine signaling pathway, focal adhesion, MAPK signaling pathway, pathways in cancer, and regulation of actin cytoskeleton were downregulated in the WAC-AS1-positive group (Fig. 9E, F).

### Relationship between chemosensitivity and WAC-AS1

**Fig. 10:**
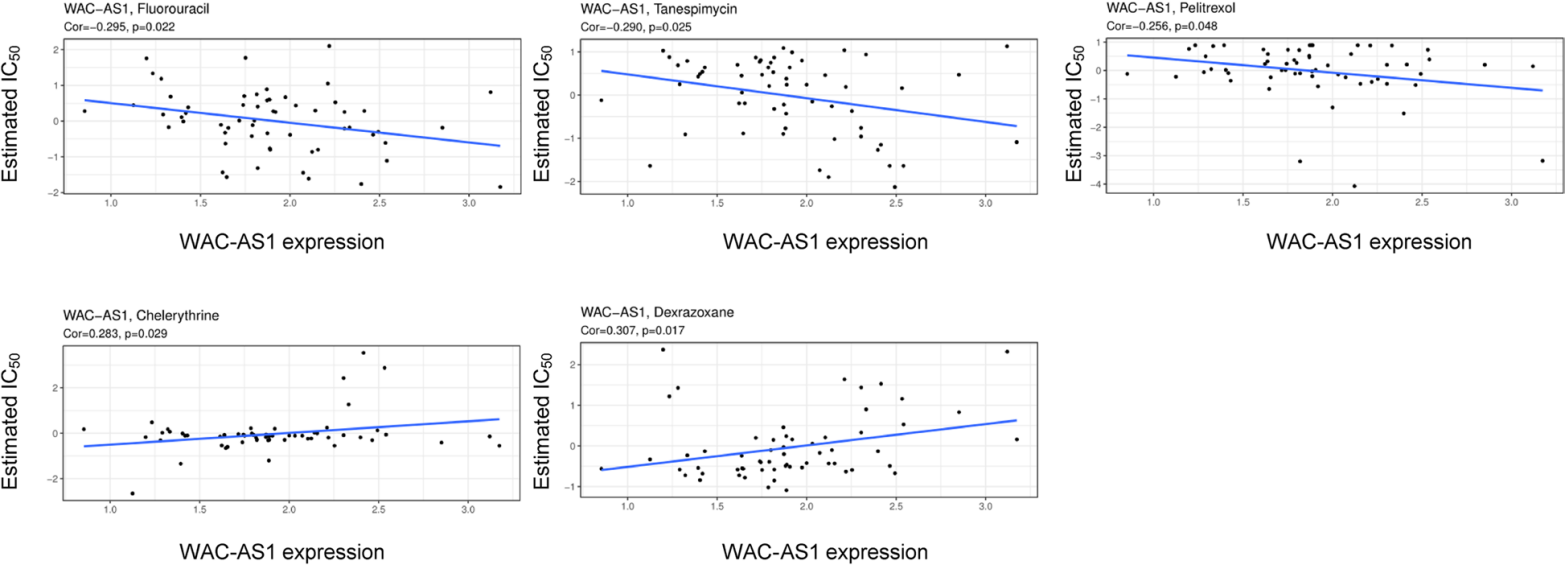
Analysis of WAC-AS1 expression with effectiveness of five common anti-cancer drugs using Cellminer database. *P* value smaller than 0.05 was considered statistically significant

Chemotherapy combined with surgery is one of the most useful treatments for various cancers, especially in early-stage tumors. Our examination of possible correlations between WAC-AS1 and commonly used antitumor reagents using the Cellminer database revealed that predictive high WAC-AS1 expression was associated with drug tolerance. In particular, we noted that tumors with high WAC-AS1 expression had markedly less response to fluorouracil, tanespimycin, and pelitrexol, while tumors with low WAC-AS1 expression were less responsive to dexrazoxane and chelerythrine (Fig. 10, Table 3).

**Table 3:** Correlation of WAC-AS1 expression with estimated IC_50_ of five chemotherapy drugs

Drug	cor	*P*-value
Dexrazoxane	0.307261	0.016938
Fluorouracil	-0.29527	0.022001
Tanespimycin	-0.28998	0.024612
Chelerythrine	0.282677	0.028641
Pelitrexol	-0.25625	0.048121

### Confirmation of the WAC-AS1 expression in multi-tumor tissue microarray (TMA)

**Fig. 11:**
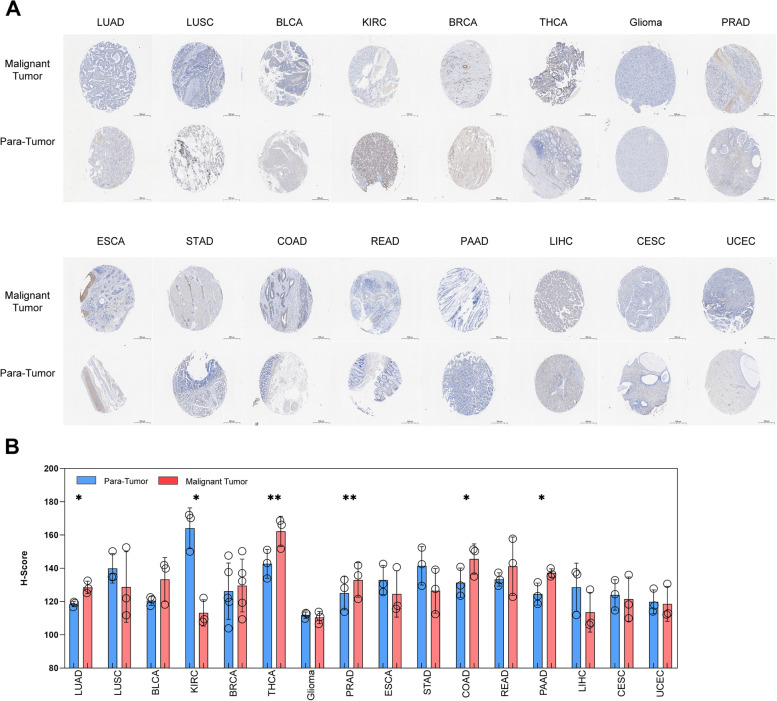
LncRNA WAC-AS1 expression in a multi-tumor tissue microarray detected by in situ hybridization (ISH). **A** Representative images of WAC-AS1 expression in malignant tumor tissues and para-tumor tissues. Scale bar, 500 μM. **B** Comparison of lncRNA WAC-AS1 expression between human malignant tumor tissues and para-tumor tissues in human different tumors. One circle represents one tissue sample. “*” indicates *P* value smaller than 0.05, “**”indicates *P* value smaller than 0.01

In order to confirm the expression of WAC-AS1 by online database analysis, we selected a multi-tumor tissue microarray and used in situ hybridization to detect the expression of WAC-AS1. The TMA template and the arrangement of the tissues are shown in Supplementary 5A-B. In situ hybridization staining of the tumor tissues indicated a predominant location of WAC-AS1 in the cytoplasm of the cells. Moreover, in twenty types of tumors, there are only two cases of small cell lung cancer tissues (SCLC), and one SKCM tissue lost in processing, resulting in less than three cases of SKCM tissues, so they are not included in the further analysis. Sixteen types of tumors (including LUAD, LUSC, BLCA, KIRC, BRCA, THCA, Glioma, PRAD, ESCA, STAD, COAD, READ, PAAD, LIHC, CESC, UCEC) were analyzed by paired t-tests to compare the H-Score of WAC-AS1 in situ hybridization between malignant tumor tissues and para-tumor tissues (Fig. 11A-B). In addition, there are two types of tumors (including OV and Lymphoma) were analyzed with two sets of t-tests for H-Score comparison between malignant tumor tissues and normal tissues (Supplementary Fig. 6A-B). The results showed that compared with para-tumor tissues, the expression of WAC-AS1 increased in LUAD, THCA, PRAD, COAD, and PAAD, while decreased in KIRC. These results are consistent with the expression of WAC-AS1 in pan-cancer analysis using the TCGA and GTEx databases. However, the expression of WAC-AS1 has the same trend with the online database analysis in BLCA and BRCA compared to the para-tumor tissue, but not was statistically significant, which may be due to the small sample size.

## Discussion

In recent years, increasing evidence has shown that lncRNA plays an important role in the occurrence and development of multiple tumors. WAC-AS1 is a novel lncRNA with few reports until now. Even so, the present reports have shown evidence for an involvement of WAC-AS1 in the prognosis and immunotherapy responses of tumor patients. For this reason, we conducted an in-depth exploration into WAC-AS1 expression in 33 tumor types included in the TCGA and GTEx databases.

Recent studies have identified WAC-AS1 as a protective factor in glioma but a risk factor in hepatocellular carcinoma, which are in line with our findings [8, 10]. Furthermore, we observed a decrease in the expression of WAC-AS1 in GBM, the advanced gliomas, compared to normal tissues, which is in contrast to its expression in LGG and is not related to the prognosis. These findings imply that WAC-AS1 is downregulated with the occurrence and development of brain glioma, and no longer plays a protective role in the prognosis of this disease. WAC-AS1 has been identified as a protective factor in ovarian carcinoma, which contrasts with our results [9, 24]. Although we observed a similar trend for a protective role of WAC-AS1 in patients with OV, the Kaplan–Meier survival analysis indicated that there was no significant difference in survival between patients with high and low WAC-AS1 expression. These data suggest that WAC-AS1 alone may not be sufficient as a prognostic factor, and its combination with other related genes, such as m6A-related lncRNAs, may be necessary for improved prediction in the prognosis of OV.

The characteristics of the tumor microenvironment play an important role in determining clinical outcomes and are considered key indicators for evaluating the efficacy of immunotherapy [25]. The ESTIMATE scores confirmed negative correlations for WAC-AS1 expression and both stromal and immune scores in the tumor microenvironment of 10 cancer types and a positive correlation in 2 cancers. Tumor-infiltrating immune cells are essential for both tumor occurrence and development, and can affect the efficacy of immunotherapy. In the present study, immune checkpoint blockades (ICBs) only worked in specific tumor-infiltrating T cell subsets [26]. Natural killer cells, inflammatory tumor-associated macrophages, dendritic cells, effector T lymphocytes, and memory T lymphocytes are immune activators or inflammatory factors within the stroma. Myeloid-derived suppressor cells, immunosuppressive tumor-associated macrophages, and regulatory T lymphocytes are known to participate in immunosuppression in the tumor microenvironment [27–29]. In this study, a significant positive correlation between WAC-AS1 expression and the presence of follicular helper T cells was observed in nine types of tumors. Moreover, the expression of WAC-AS1 was significantly positively correlated with the infiltration of CD8 T lymphocytes in eight types of tumors. Among them, WAC-AS1 was identified as a protective factor for the prognosis of PAAD, which may be related to the immune infiltration of CD8 T lymphocytes in PAAD with high WAC-AS1 expression. The expression of WAC-AS1 was significantly negatively correlated with the presence of M0 macrophages in five types of tumors. Therefore, the poor prognosis of LIHC patients with high WAC-AS1 expression may be related to macrophage infiltration.

Our analysis using the TISIDB database revealed significant associations between almost all immune-related genes, including MHC, immunostimulatory and immunoinhibitory factors, chemokine, and chemokine receptor proteins, as well as the immune checkpoint proteins, and WAC-AS1 expression in all types of tumors except for UCS, MESO, and SARC. Therefore, our research findings illustrate that WAC-AS1 has a broad tumor applicability and highlight that WAC-AS1 expression is strongly linked to immune cell behaviors and immune-related factors in several cancer types.

TMB and MSI also contribute to ICBs. We found significant correlations of WAC-AS1 expression with TMB and MSI in diverse types of tumors. TMB, defined as the number of non-inherited mutations per megabase among the interrogated genomic sequence, is currently recognized as a key player in generating immunogenic neopeptides on the MHC of tumor cell surface, and has been shown to positively affect the response of patients to ICBs [22, 30–32]. MSI occurs in short, repeated DNA sequences, especially in colorectal cancer. Several studies have reported that high-frequency MSI can be used as an independent predictor for clinical characteristics and prognosis in colorectal cancer [23, 33]. Our findings indicate that changes in TMB and MSI in tumors due to the expression of WAC-AS1 may have an impact on patients’ response to ICB therapy. Despite the significant correlations of WAC-AS1 with TMB and MSI, the correlation coefficients across all types of cancers were less than 0.5, suggesting that WAC-AS1 expression may not be used as an independent predictor for patients’ response to ICBs.

The role of WAC-AS in regulating cancer development is contradictory. In this study, we explored the mechanisms and involvement of WAC-AS1 in LIHC and LGG. The results revealed that WAC-AS1 played a dual role, acting as both a risk factor and a protective factor, which warrants further investigation. The GSVA and GSEA results showed opposite correlations between WAC-AS1 and some pathways in LIHC and LGG tumors. For instance, there was a significant positive correlation between WAC-AS1 expression and the TGF-beta signaling and glycolysis in LIHC, while a negative correlation was observed in LGG. The TGF-beta signaling participates in the progression of multiple types of cancers and can regulate gastric cell invasion and metastasis by mediating tumor-associated macrophages. Therefore, TGF-beta may function as an immuno-inhibitor in tumor immune responses [34]. An increase in glycolysis can promote malignant behaviors, such as proliferation, invasion, and metastasis, in various types of cancers. Accumulating evidence has shown that upregulation of glycolysis mediates immunotherapy resistance by regulating the infiltration of immune cells into the tumor immune microenvironment [35, 36]. Our findings are also consistent with the study by Xia et al. [10], showing that WAC-AS1 can promote glycolysis in LIHC. Therefore, high WAC-AS1 expression activates the TGF-beta signaling pathway and promotes glycolysis in LIHC, whereas WAC-AS1 is found to have the opposite effect in LGG. Similarly, WAC-AS1 and oxidative phosphorylation were significantly negatively correlated in LIHC but positively correlated in LGG. The suppression of oxidative phosphorylation is known to improve immune responses [37].The different functions of WAC-AS1 observed in the related signaling pathways resulted in different prognoses for patients with LIHC and LGG.

In general, WAC-AS1 affects the tumor immune response either on its own or through signal transduction, and it has opposing functions in different types of cancer. We only confirmed the expression of WAC-AS1 in multi-tumor tissue microarray. Although the ISH results showed in 6 types of tumors, the expression of WAC-AS1 was consistent with the online database analysis, more evidence should be obtained by in-depth experiments using different tumor types.

In summary, our preliminary analysis of WAC-AS1 expression in 33 human tumors revealed diverse WAC-AS1 expression in cancer, suggesting that WAC-AS1 is likely to be an important player in tumorigenesis. The effect of WAC-AS1 expression on tumor immunity and the regulatory process also appears to vary with tumor type. Nevertheless, our current results help to reveal the role of WAC-AS1 in tumor initiation and progression and have important implications for future validation of WAC-AS1-based experiments.

## Supplementary Information

**Additional file 1: Supplementary Figure 1.** Association between WAC-AS1 expression and tumor grade in (A) Cholangiocarcinoma (CHOL), (B) Head and neck aquamous cell carcinoma (HNSC), (C) Brain low grade glioma (LGG), (D) Liver hepatocellular carcinoma (LIHC).

**Additional file 2: Supplementary Figure 2.** Association between WAC-AS1 expression and patients age in (A) Glioblastoma multiforme (GBM), (B) Brain low grade glioma (LGG), (C) Testicular germ cell tumors (TGCT), (D) Uterine corpus endometrial carcinoma (UCEC).

**Additional file 3: Supplementary Figure 3.** The tumor microenvironment-based gene signature in high and low WAC-AS1 expression groups in 33 types of tumors of The Cancer Genome Atlas (TCGA) cohort. “*”, “**”, “***” and “****” indicate *P* values smaller than 0.05, 0.01, 0.001 and 0.0001 respectively. “ns” means no significance.

**Additional file 4: Supplementary Figure 4.** The correlation analysis of WAC-AS1 in SARC, LIHC and LGG. Top 10 genes most positively and top 10 genes most negatively associated with WAC-AS1 were shown in heatmap.

**Additional file 5: Supplementary Figure 5.** The TMA template and the arrangement of the tissues.

**Additional file 6: Supplementary Figure 6.** LncRNA WAC-AS1 expression in OV and Lymphoma in TMA by ISH detection.

**Additional file 7: Supplementary Table 1.** Correlations of WAC-AS1 expression with tumor microenvironment scores by TIMER across 33 types of tumor.

**Additional file 8: Supplemental Table 2.** Correlation of WAC-AS1 expression with immune cell infiltration in 33 types of tumor.

## Data Availability

All data, models, and code generated or used during the study appear in the submitted article.

## Abbreviations

LncRNA: Long non-coding RNA
WAC-AS1: WAC-antisense RNA1
TCGA: The Cancer Genome Atlas
GTEx: Genotype Tissue-Expression
FPKM: Fragments per kilobase million
OS: Overall survival
TMB: Tumor mutational burden
MSI: Microsatellite instability
GSEA: Gene set enrichment analysis
GSVA: Gene set variation analysis
ICBs: Immune checkpoint blockades
MHC: Major histocompatibility complex
ISH: In situ hybridization
TMA: Tissue microarray
H-Score: Histochemistry score
ACC: Adrenocortical carcinoma
BLCA: Bladder urothelial carcinoma
BRCA: Breast invasive carcinoma
CESC: Cervical squamous cell carcinoma and endocervical adenocarcinoma
CHOL: Cholangiocarcinoma
COAD: Colon adenocarcinoma
DLBC: Lymphoid neoplasm diffuse large B-cell lymphoma
ESCA: Esophageal carcinoma
GBM: Glioblastoma multiforme
HNSC: Head and neck squamous cell carcinoma
KICH: Kidney chromophobe
KIRC: Kidney renal clear cell carcinoma
KIRP: Kidney renal papillary cell carcinoma
LAML: Acute myeloid leukemia
LGG: Brain lower grade glioma
LIHC: Liver hepatocellular carcinoma
LUAD: Lung adenocarcinoma
LUSC: Lung squamous cell carcinoma
MESO: Mesothelioma
OV: Ovarian serous cystadenocarcinoma
PAAD: Pancreatic adenocarcinoma
PCPG: Pheochromocytoma and paraganglioma
PRAD: Prostate adenocarcinoma
READ: Rectum adenocarcinoma
SARC: Sarcoma
SCKM: Skin cutaneous melanoma
STAD: Stomach adenocarcinoma
TGCT: Testicular germ cell tumor
THCA: Thyroid carcinoma
THYM: Thymoma
UCEC: Uterine corpus endometrial carcinoma
UCS: Uterine carcinosarcoma
UVM: Uveal melanoma
