# Supplementary figures and images for: LncRNA WAC-AS1 expression in human tumors correlates with immune infiltration and affects prognosis
Source: Hereditas. 2023 May 30;160:26. doi: 10.1186/s41065-023-00290-z (PMC10227977; doi:10.1186/s41065-023-00290-z)

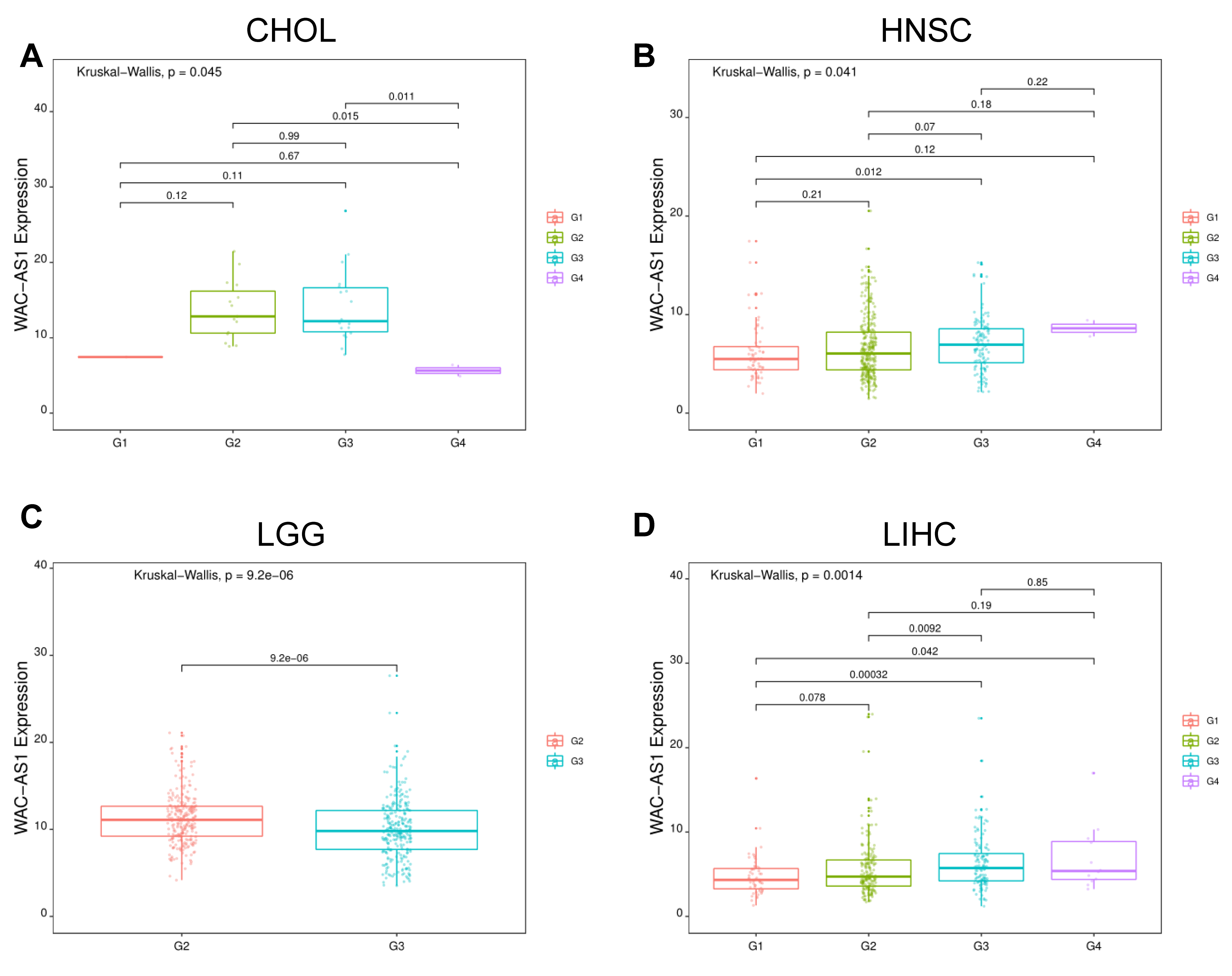

Supplement: Supplementary file 1 — Additional file 1: Supplementary Figure 1. Association between WAC-AS1 expression and tumor grade in (A) Cholangiocarcinoma (CHOL), (B) Head and neck aquamous cell carcinoma (HNSC), (C) Brain low grade glioma (LGG), (D) Liver hepatocellular carcinoma (LIHC). [file 41065_2023_290_MOESM1_ESM.tif]

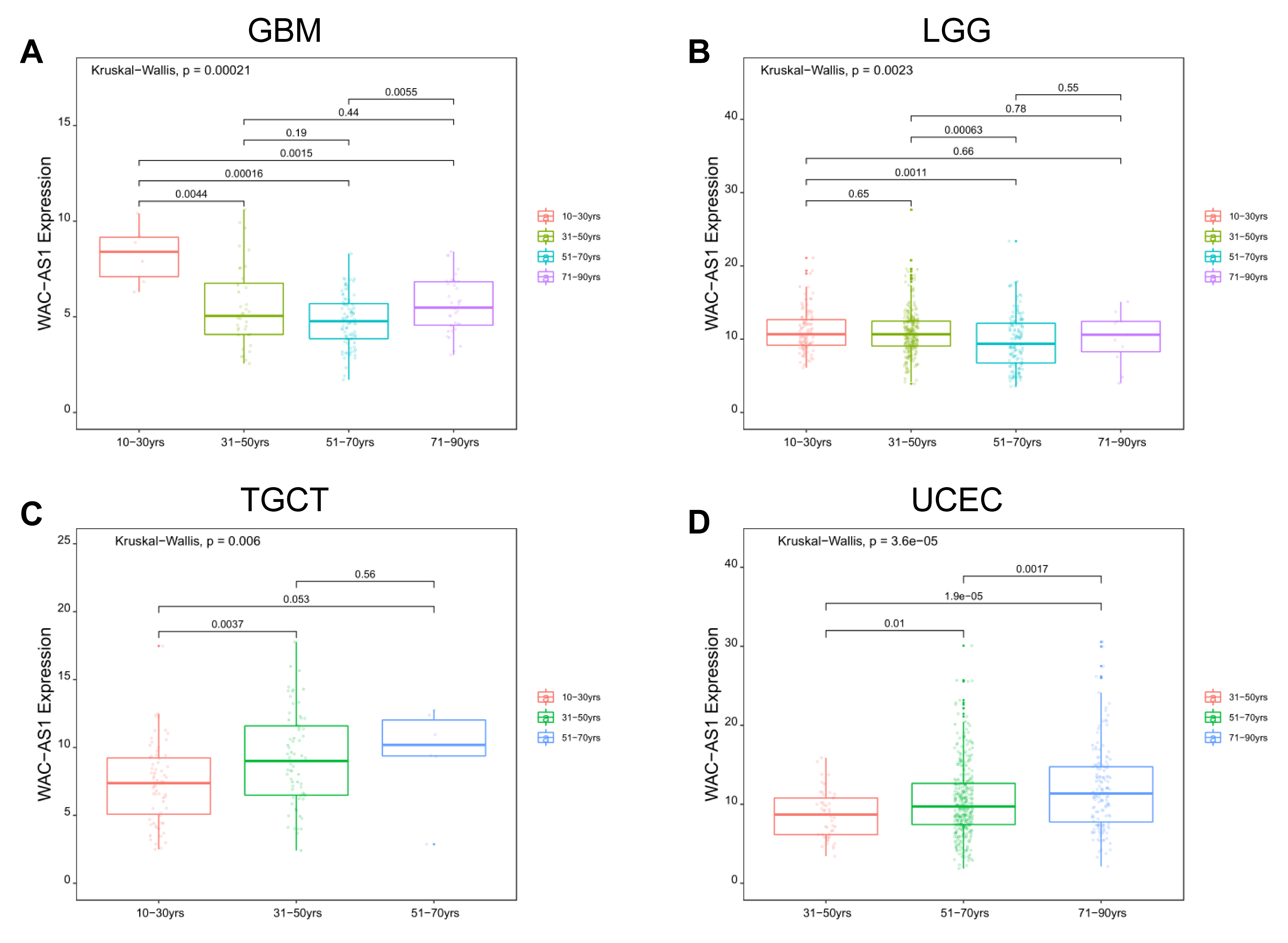

Supplement: Supplementary file 2 — Additional file 2: Supplementary Figure 2. Association between WAC-AS1 expression and patients age in (A) Glioblastoma multiforme (GBM), (B) Brain low grade glioma (LGG), (C) Testicular germ cell tumors (TGCT), (D) Uterine corpus endometrial carcinoma (UCEC). [file 41065_2023_290_MOESM2_ESM.tif]

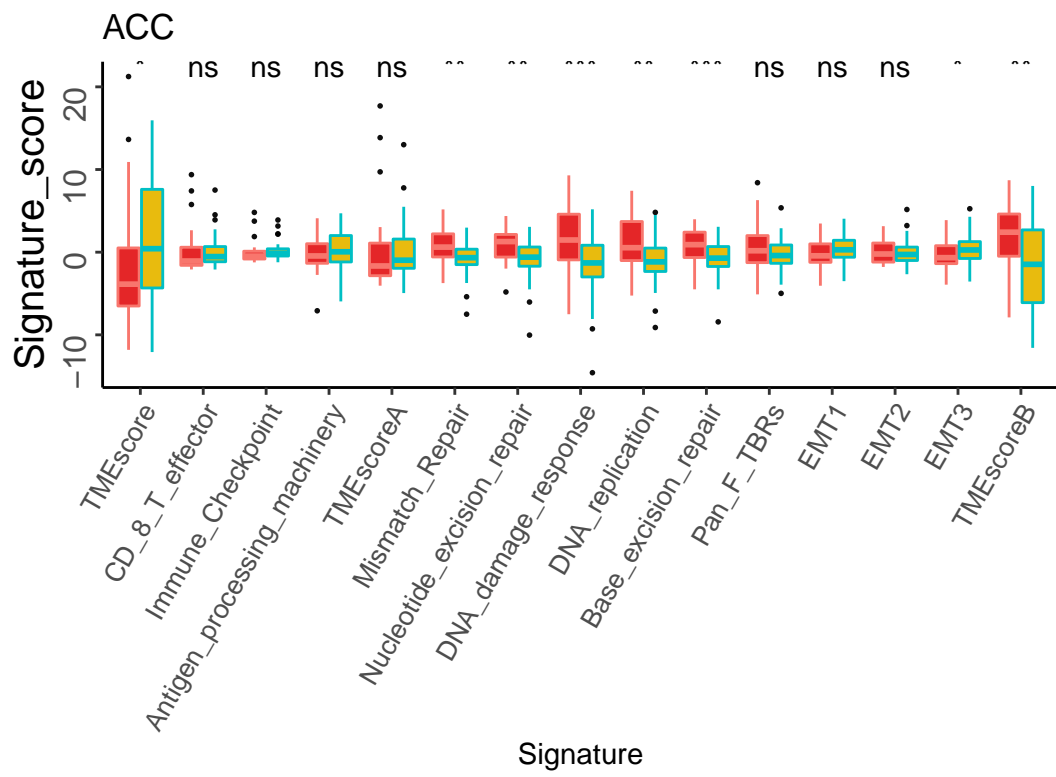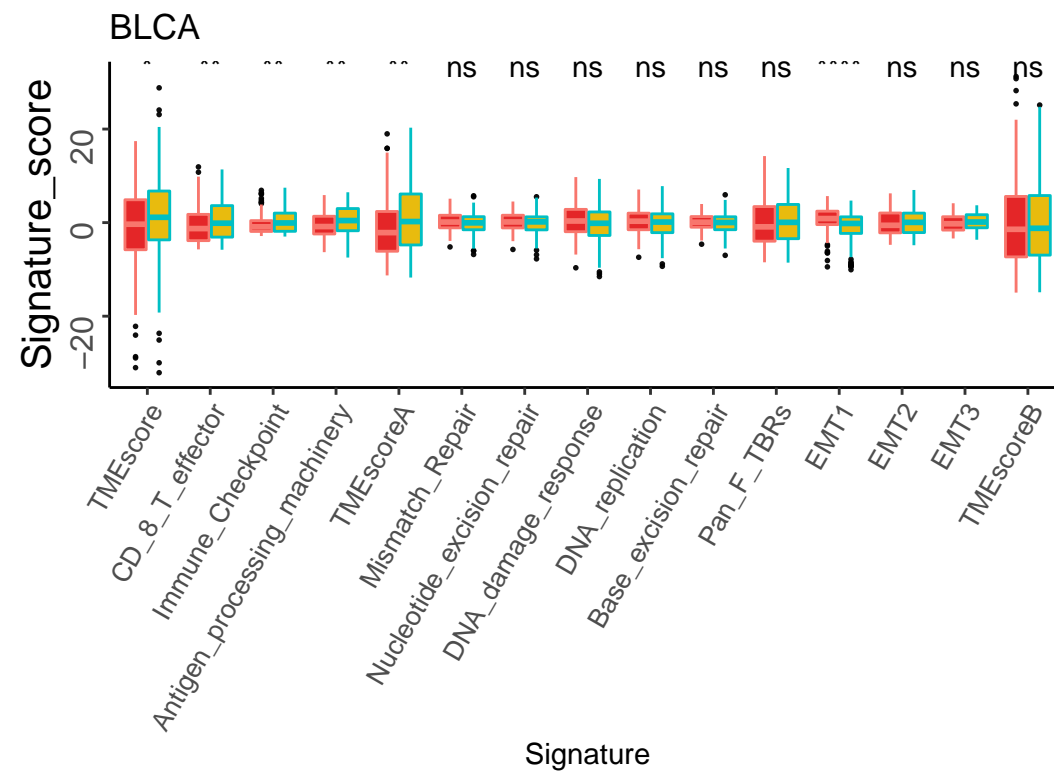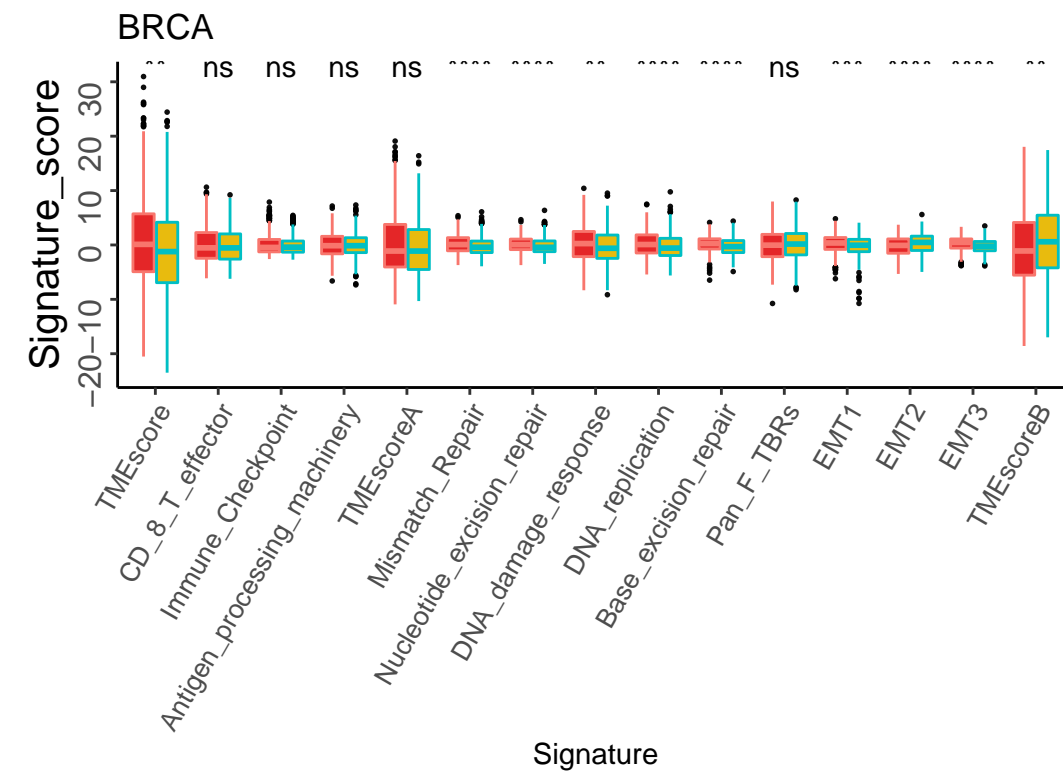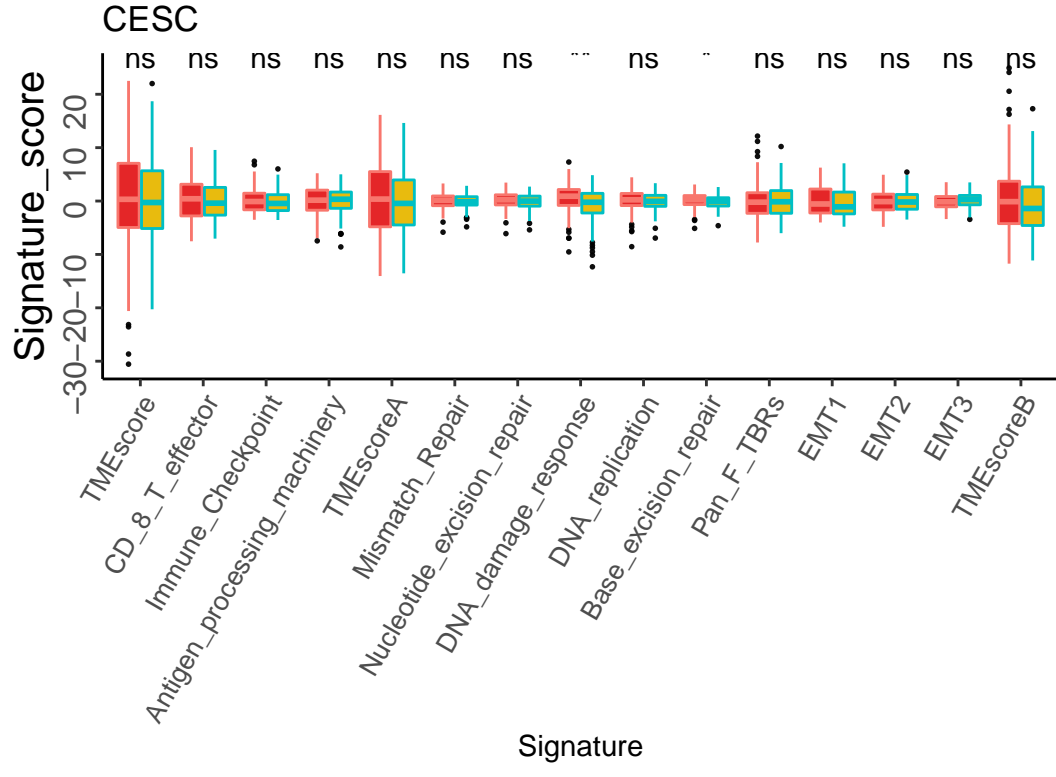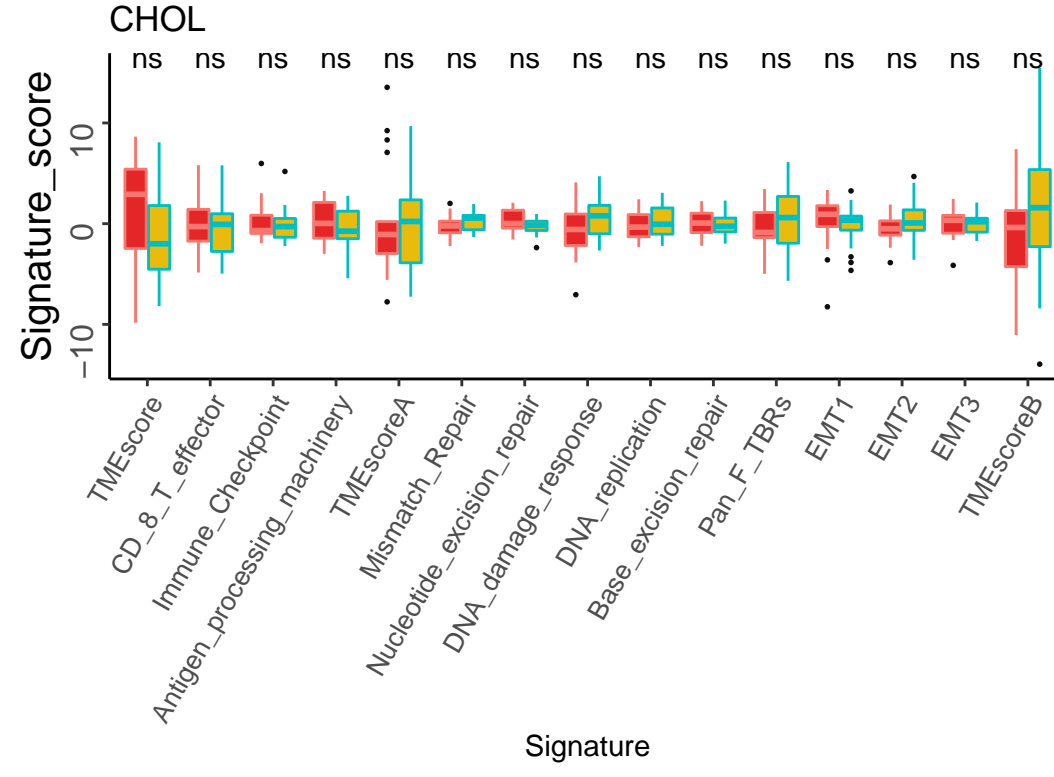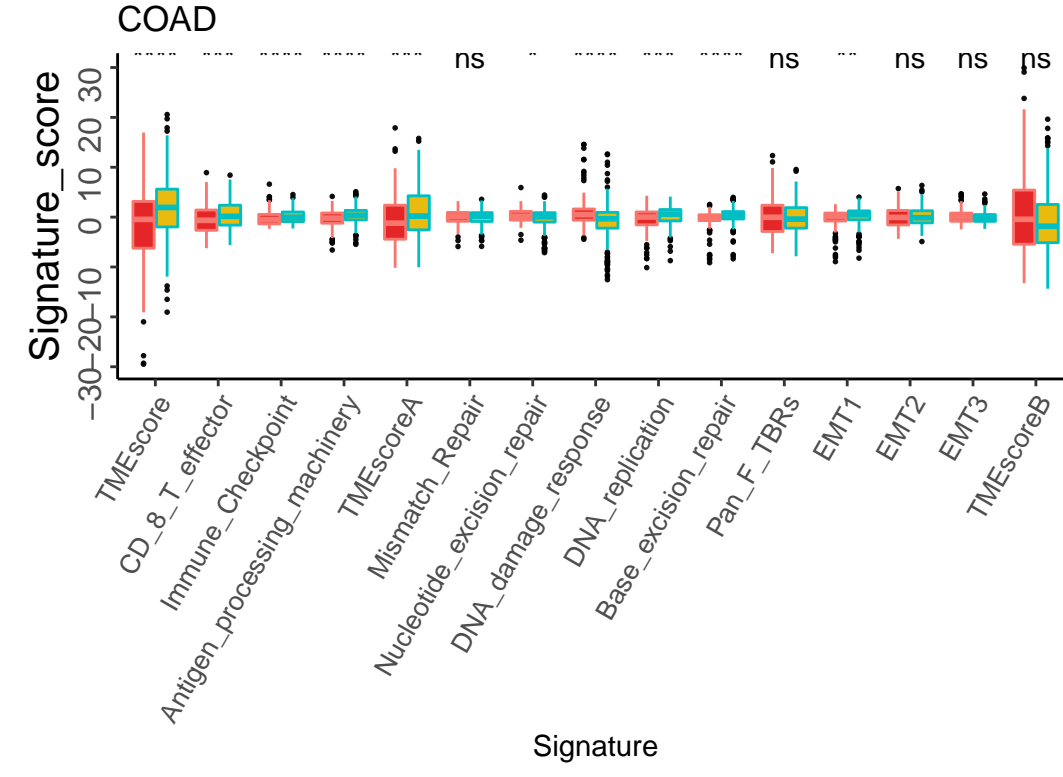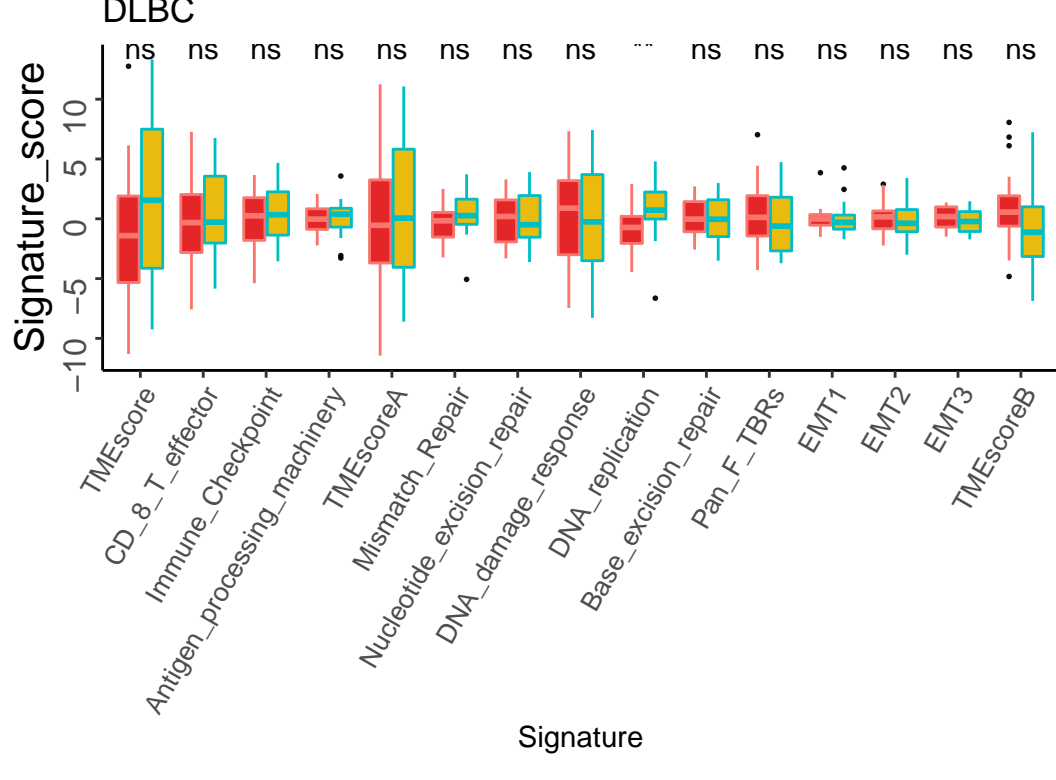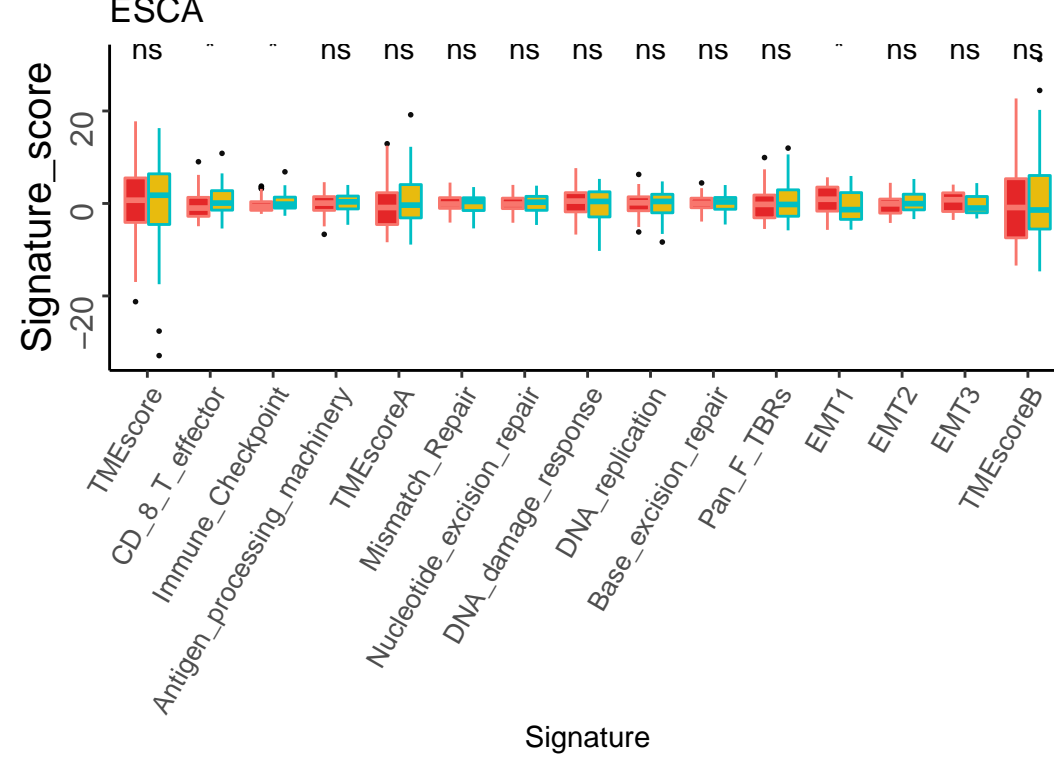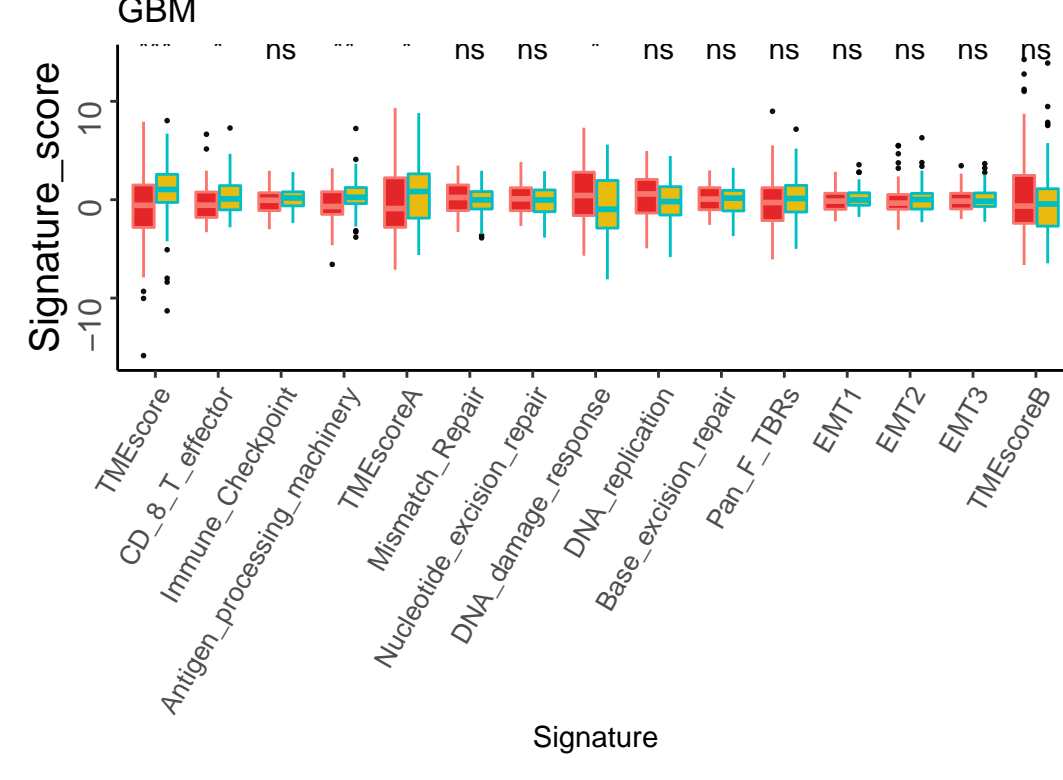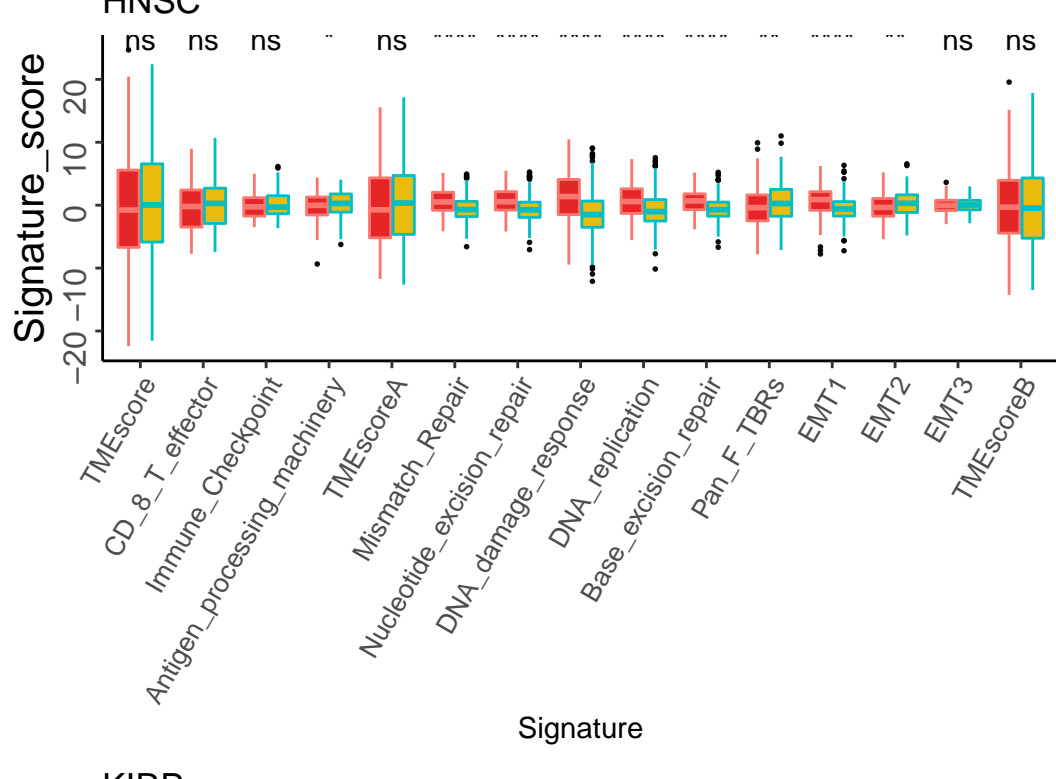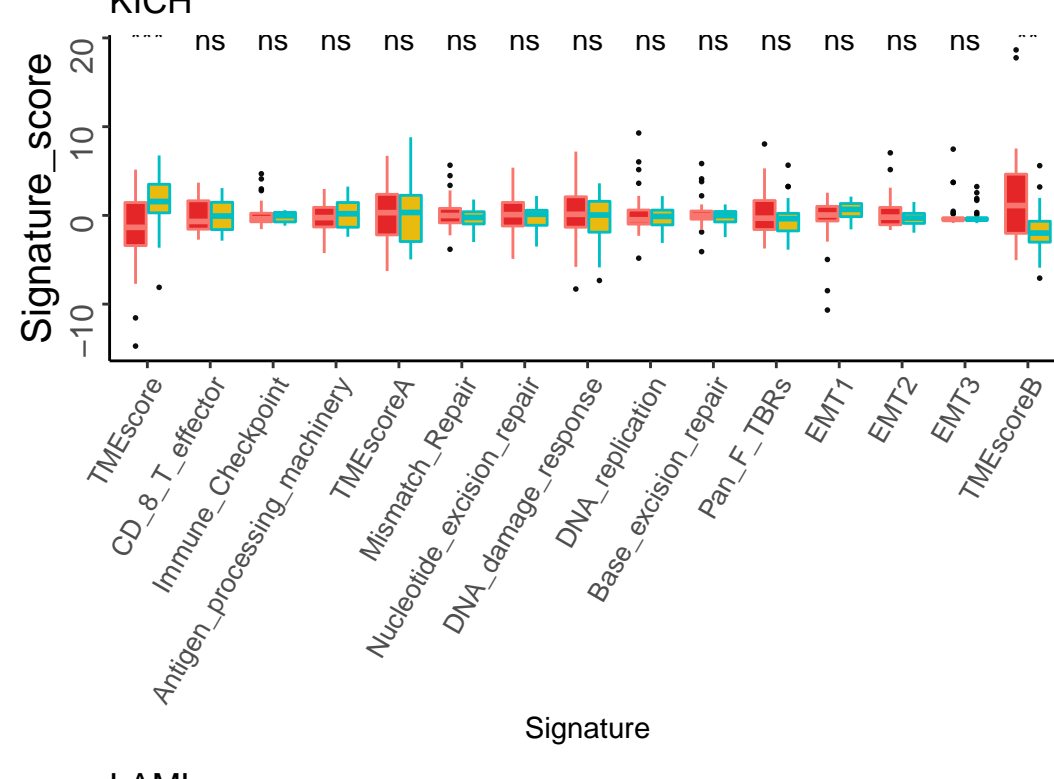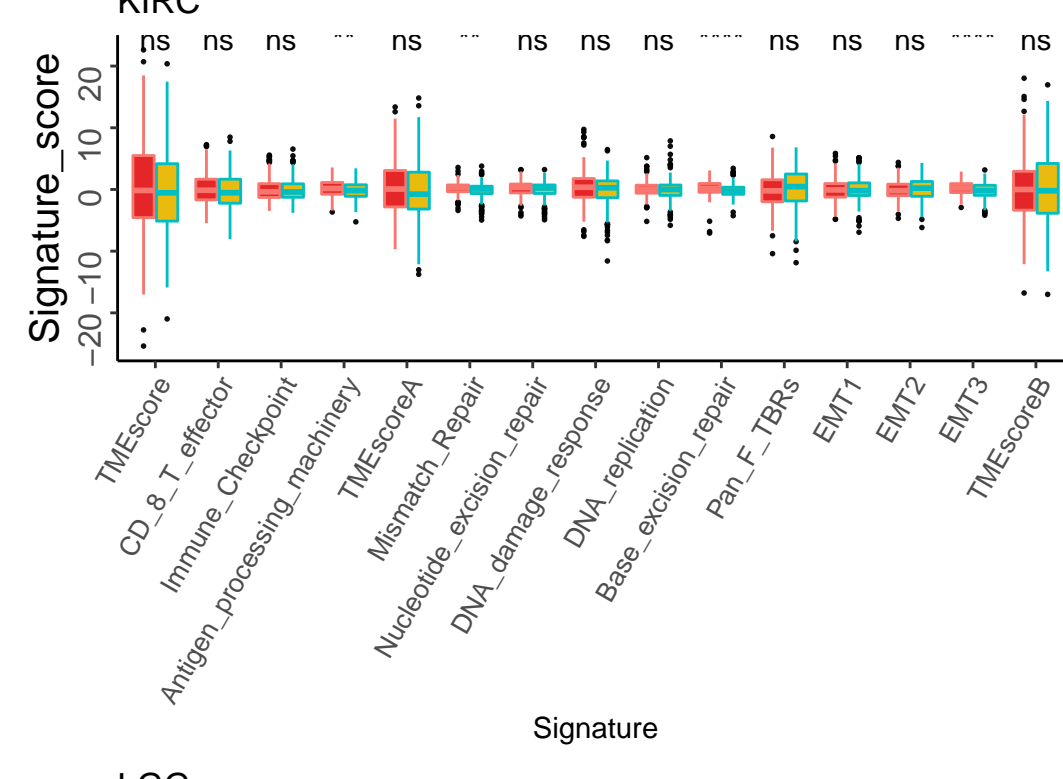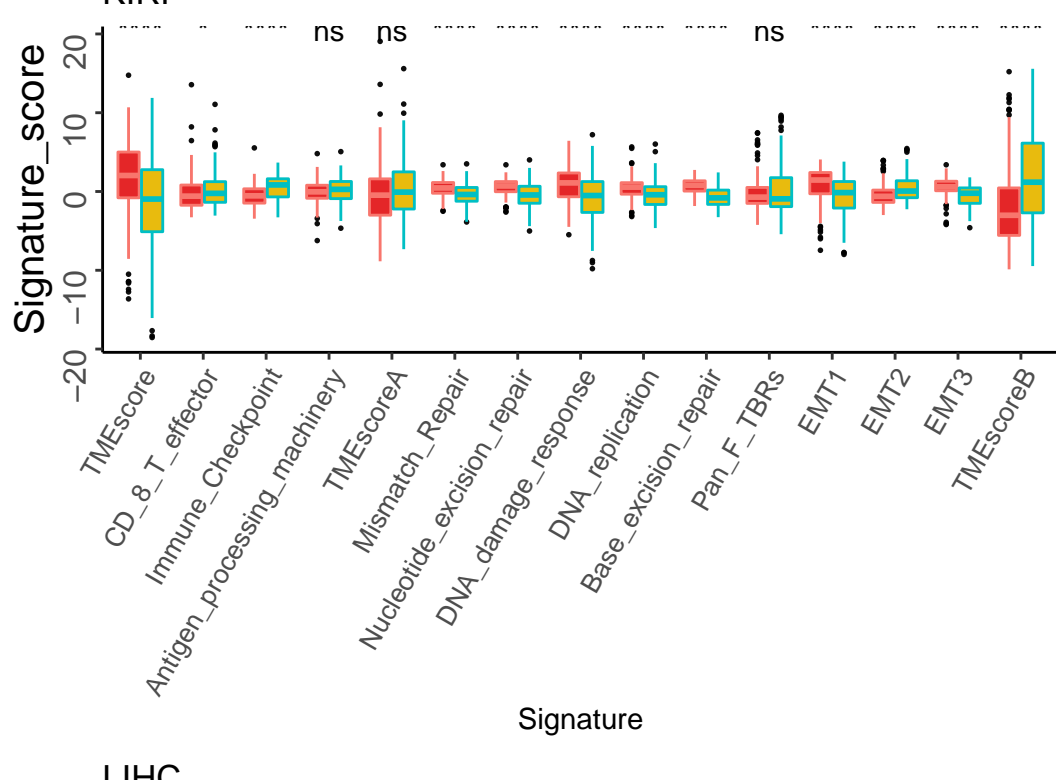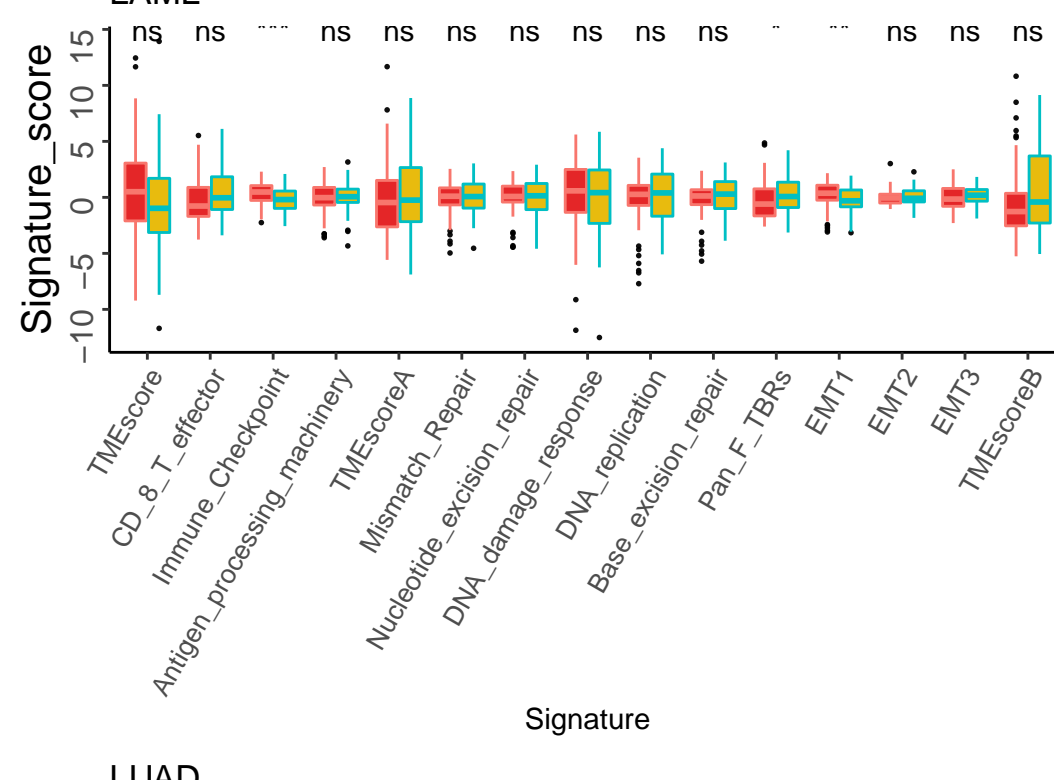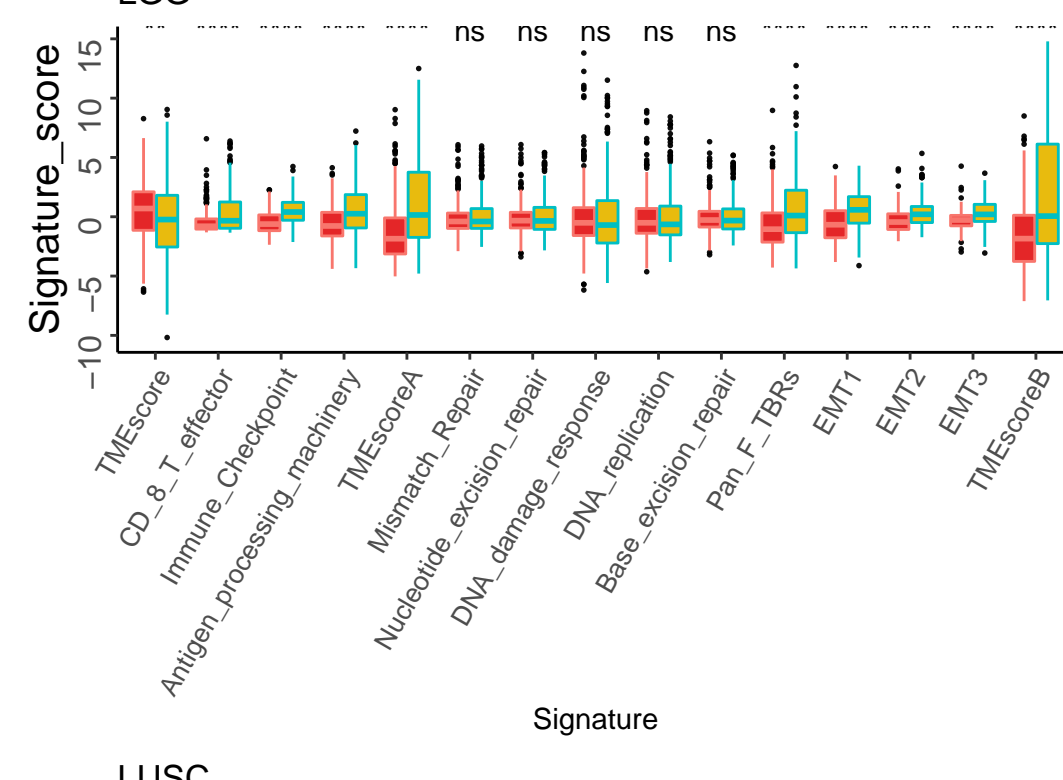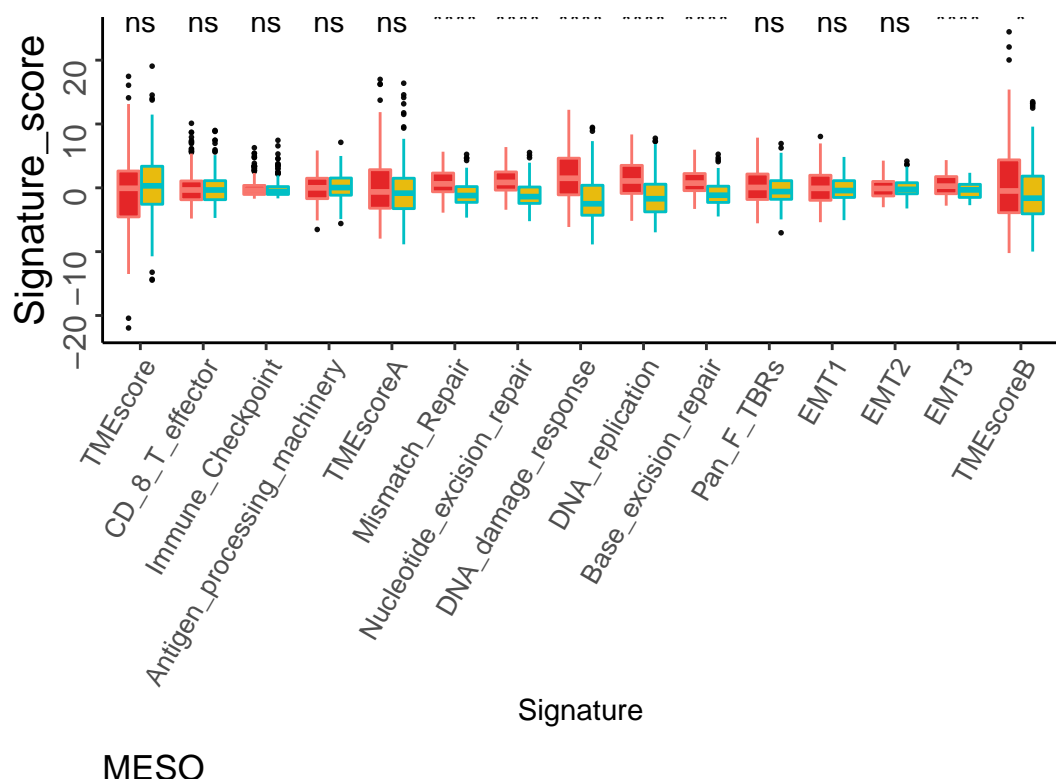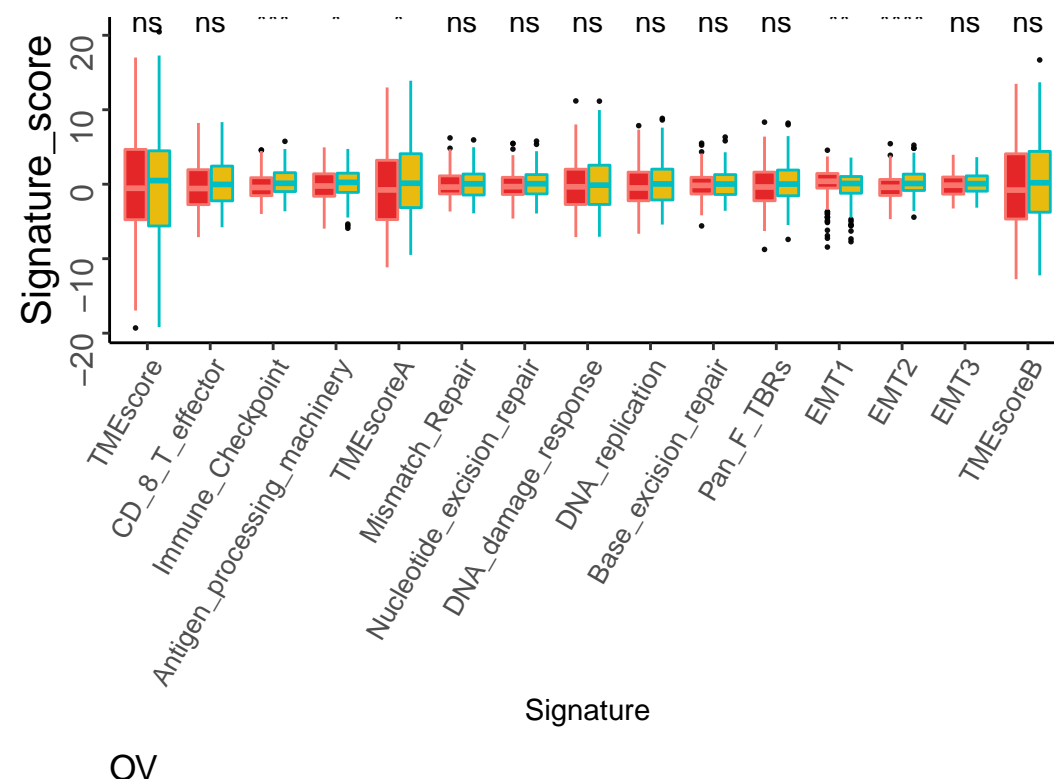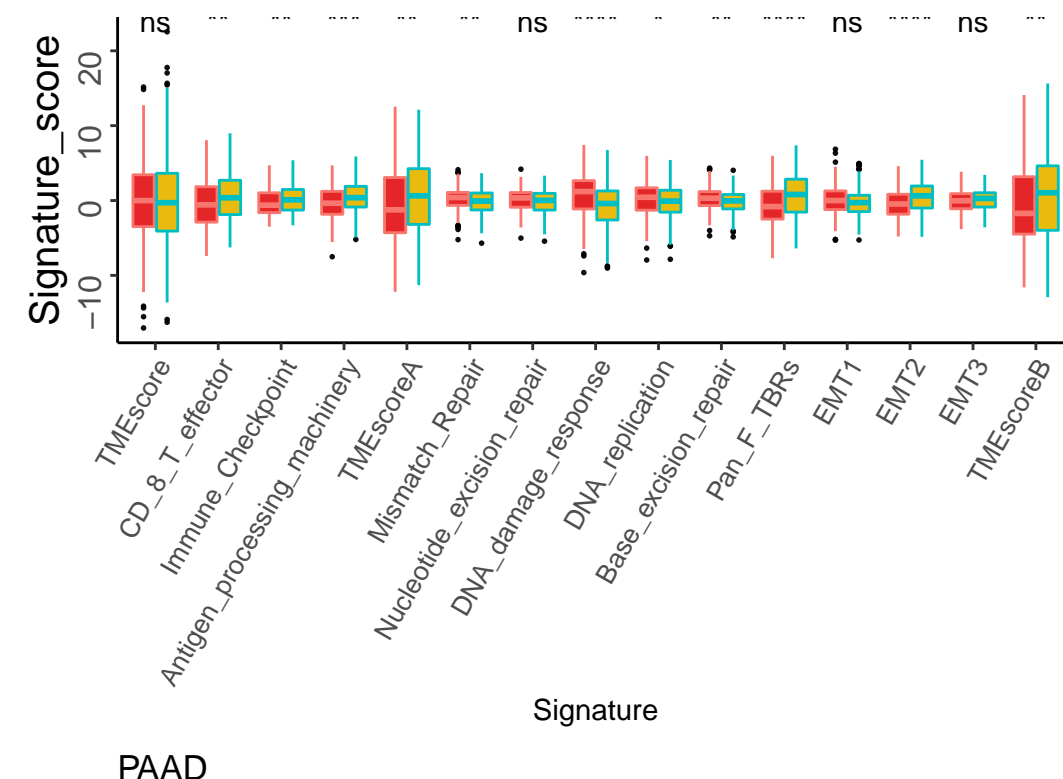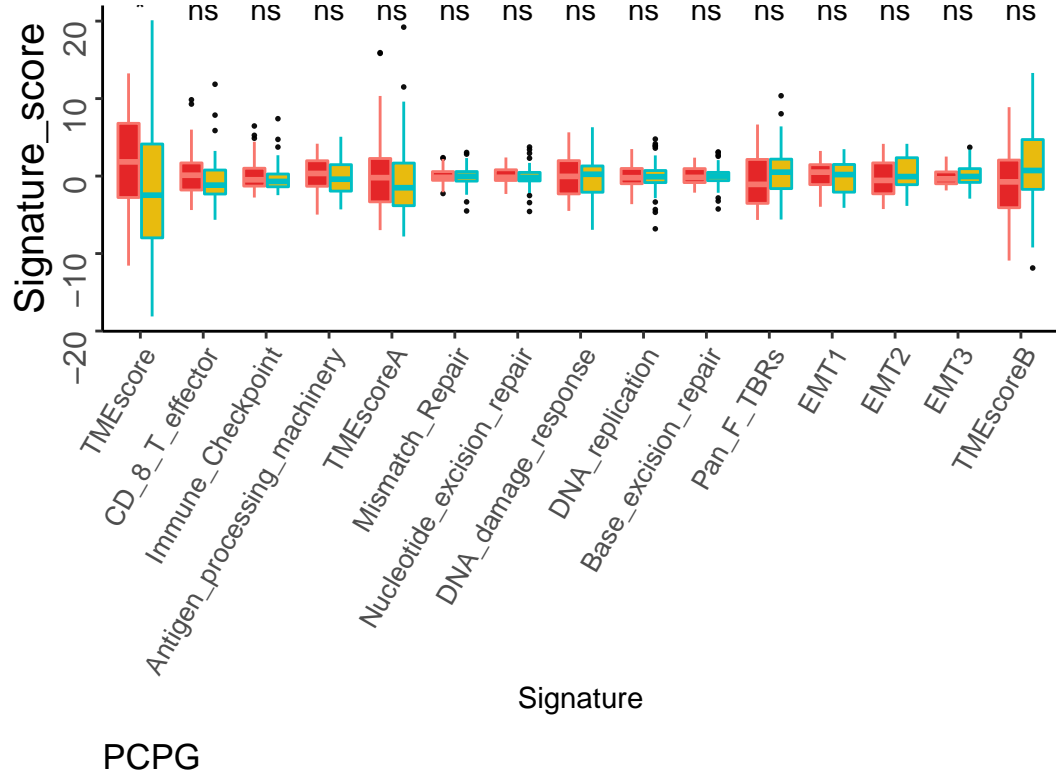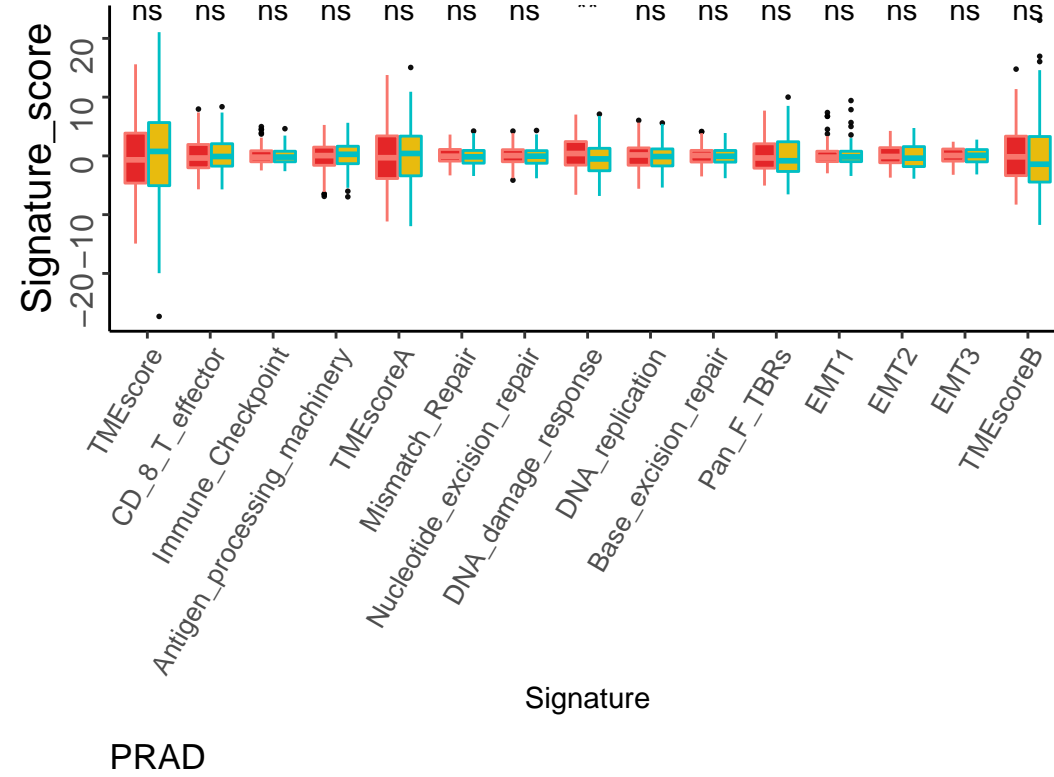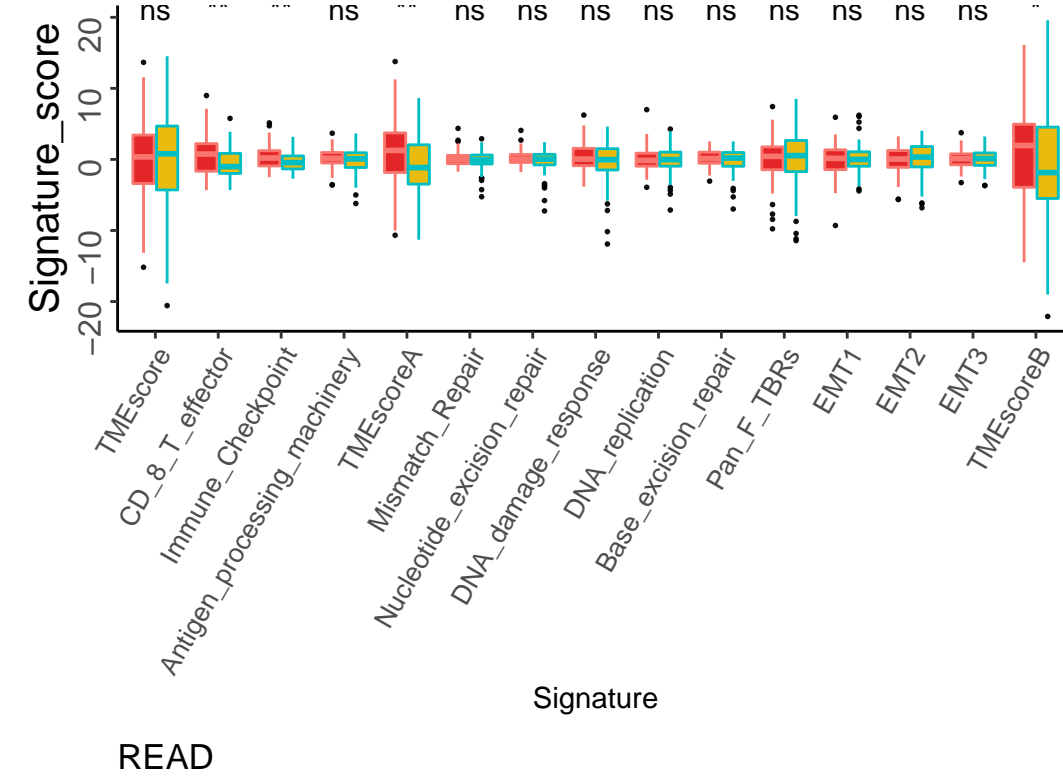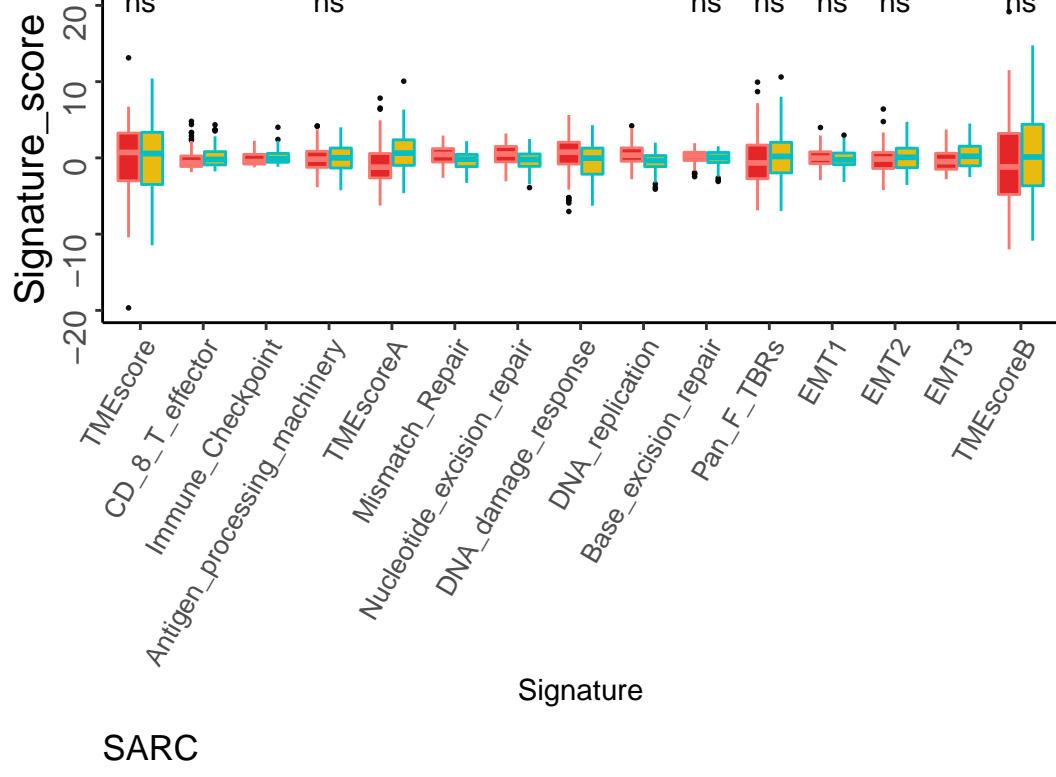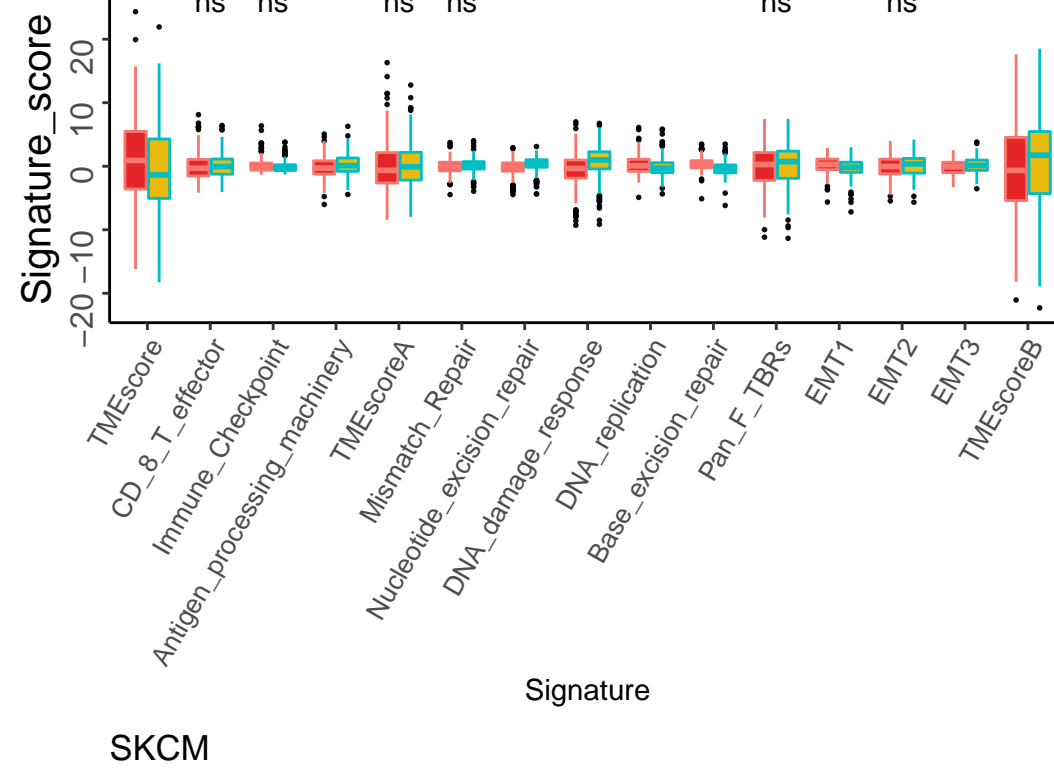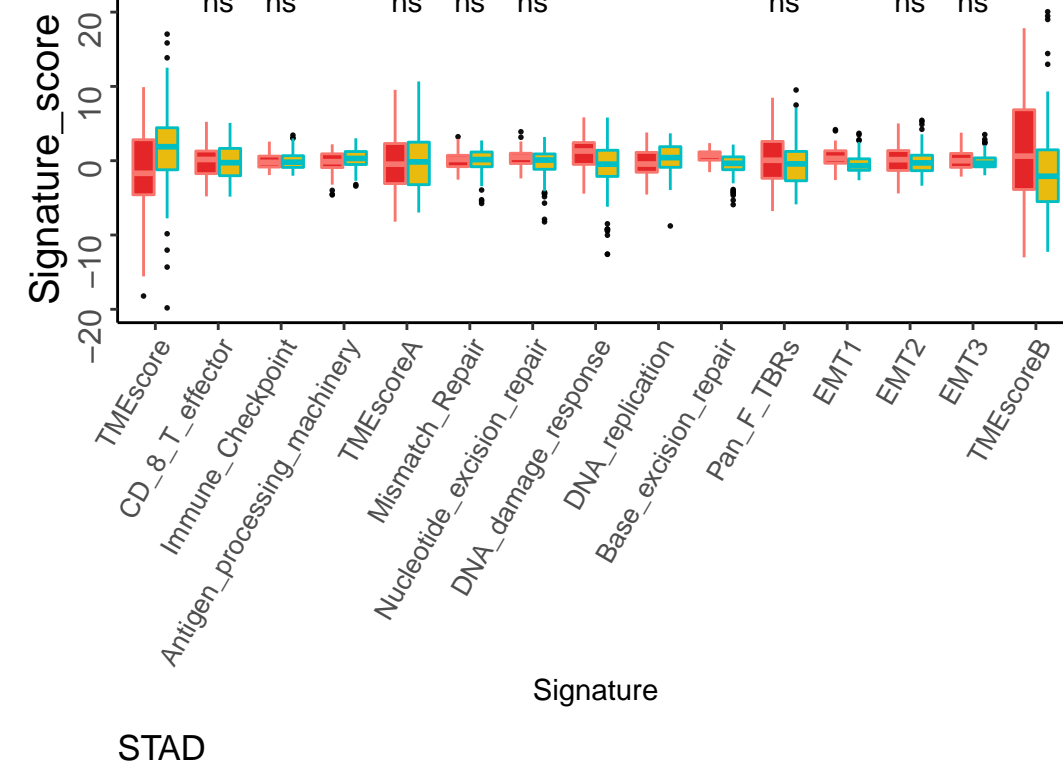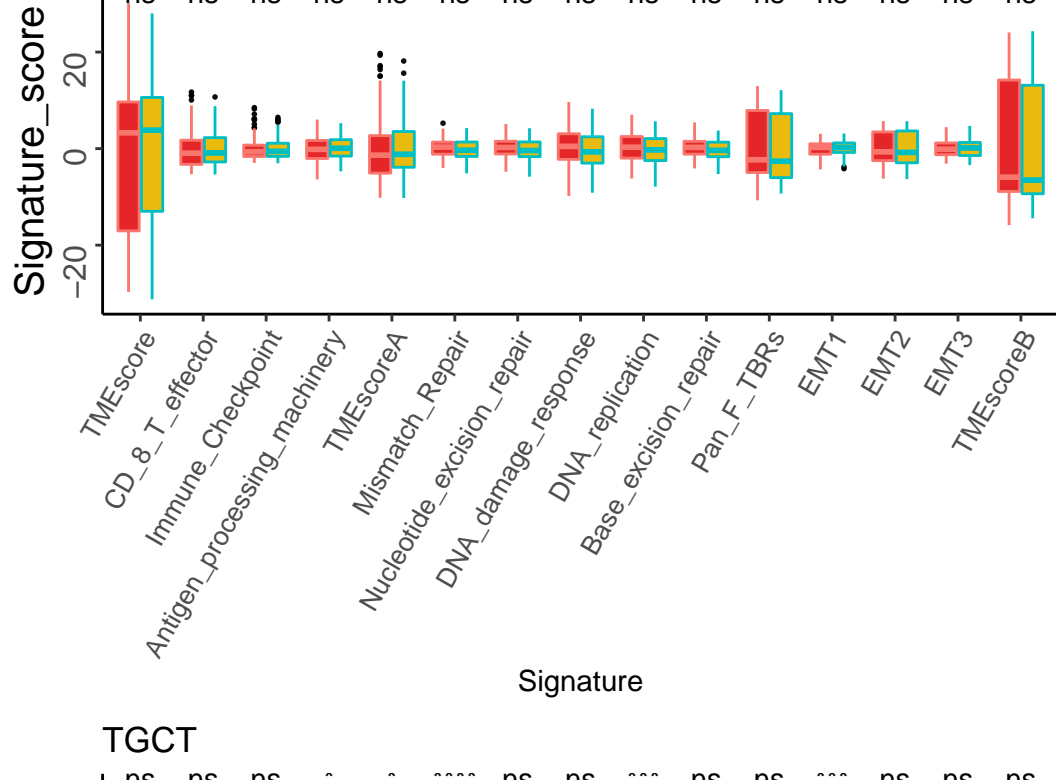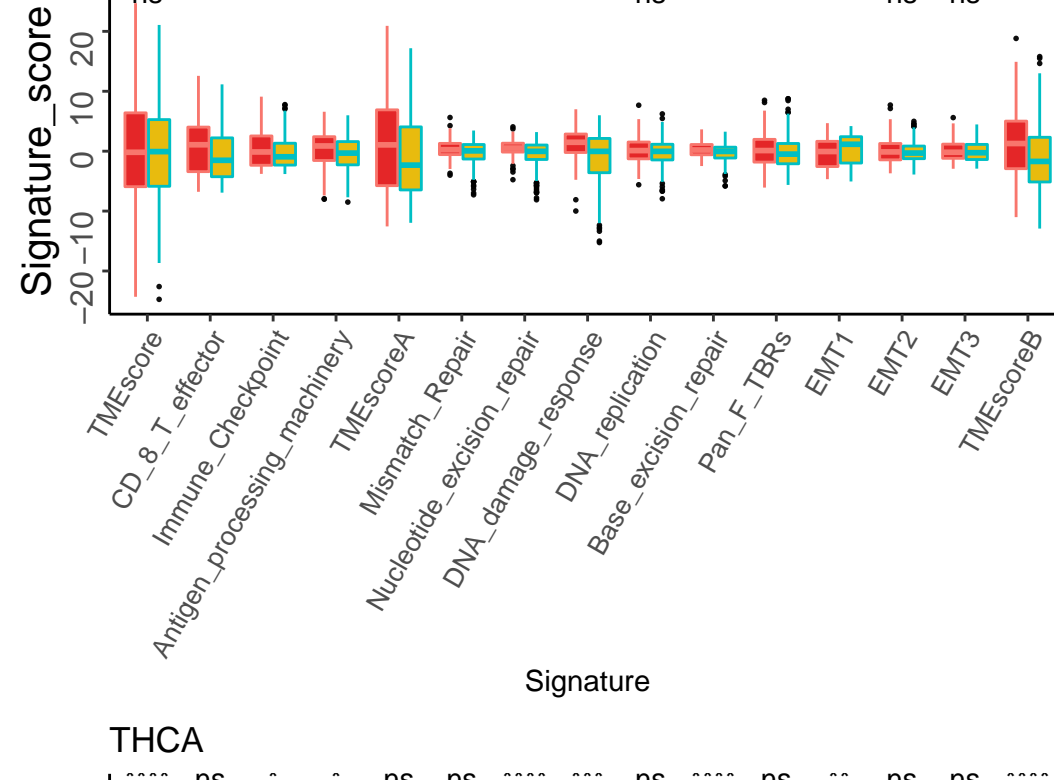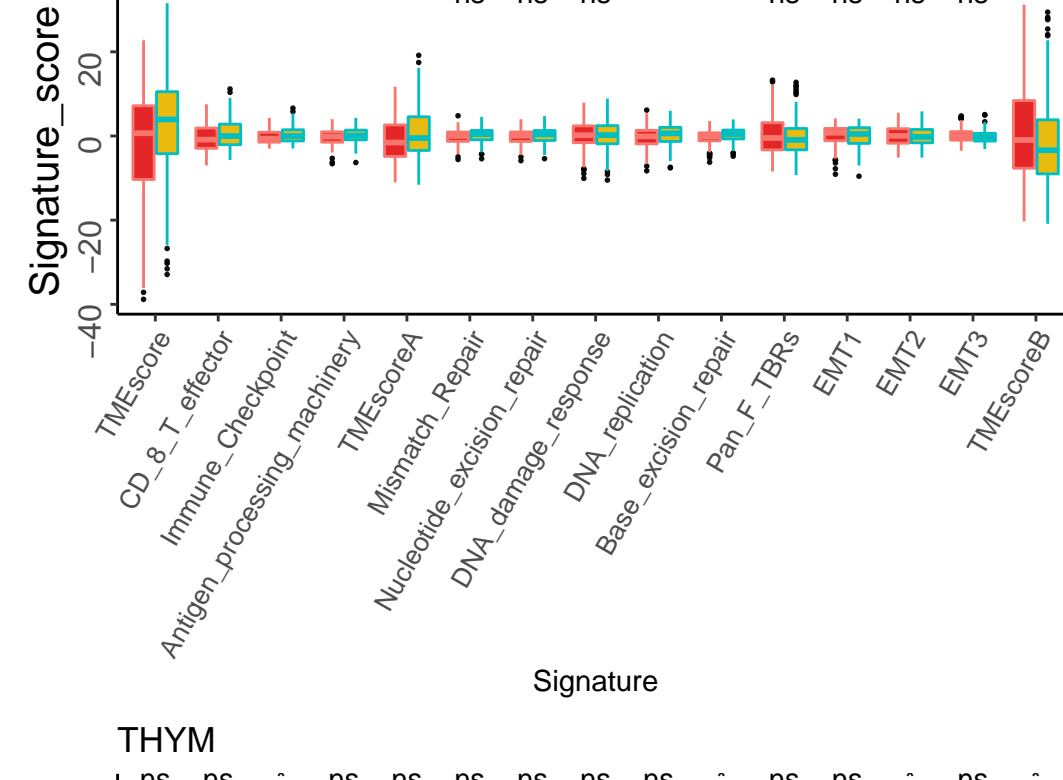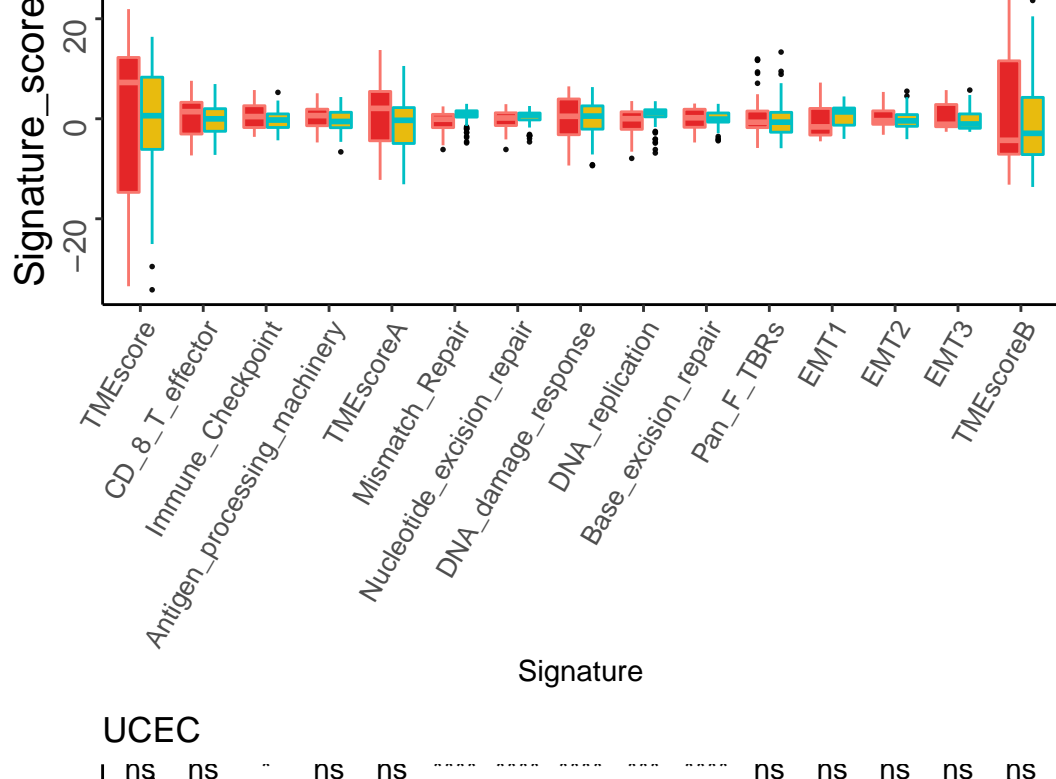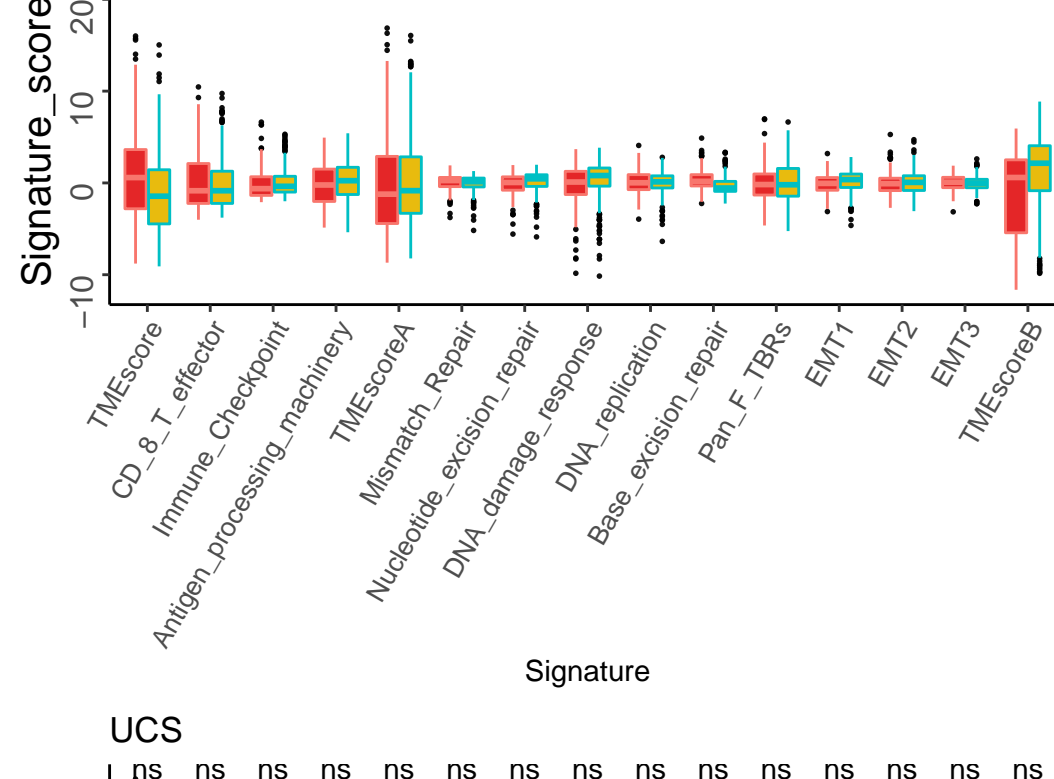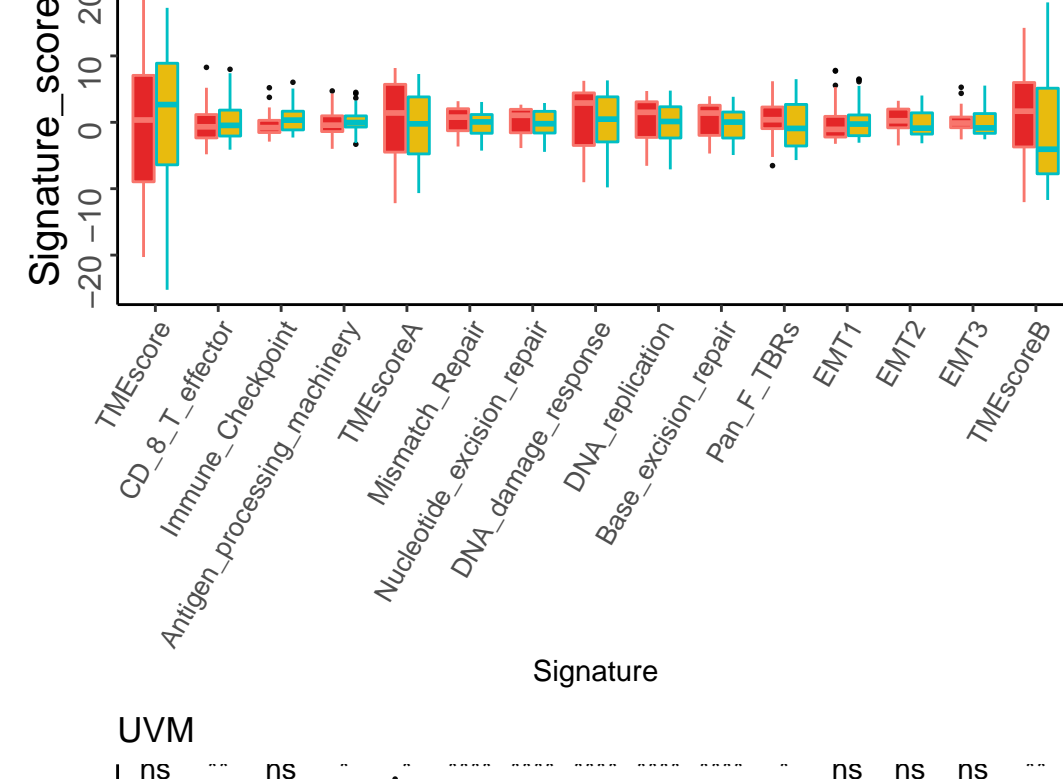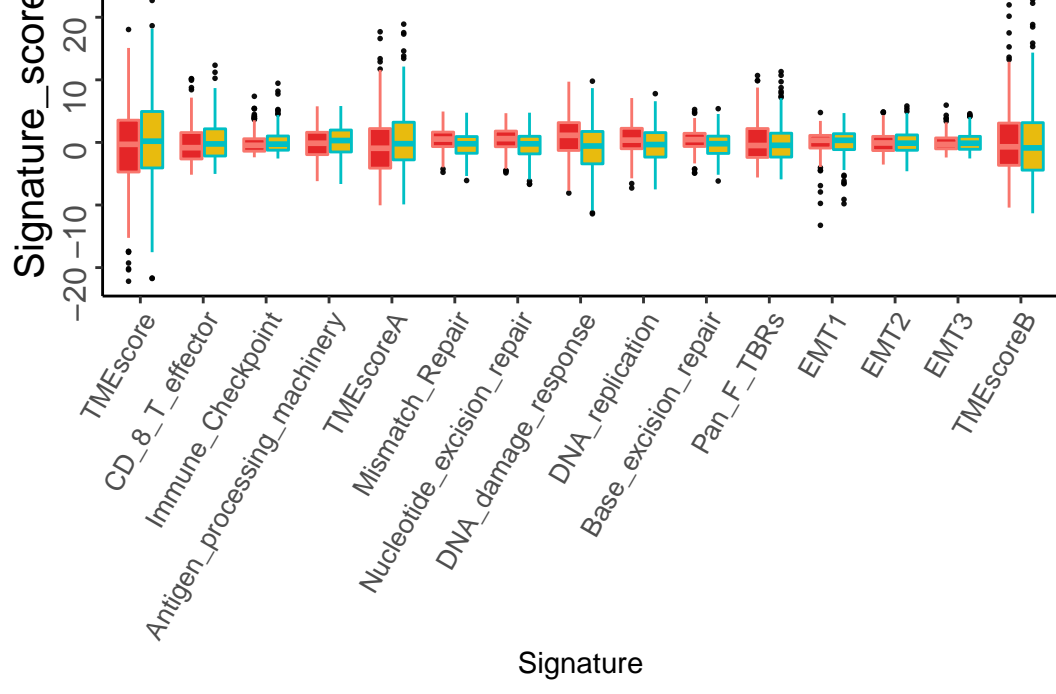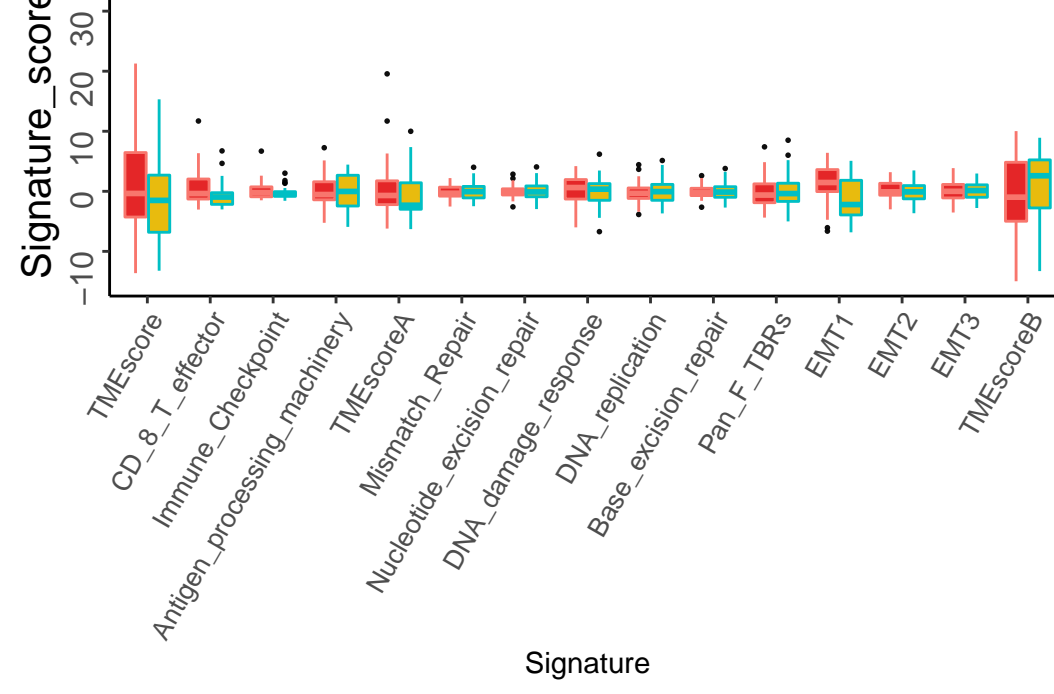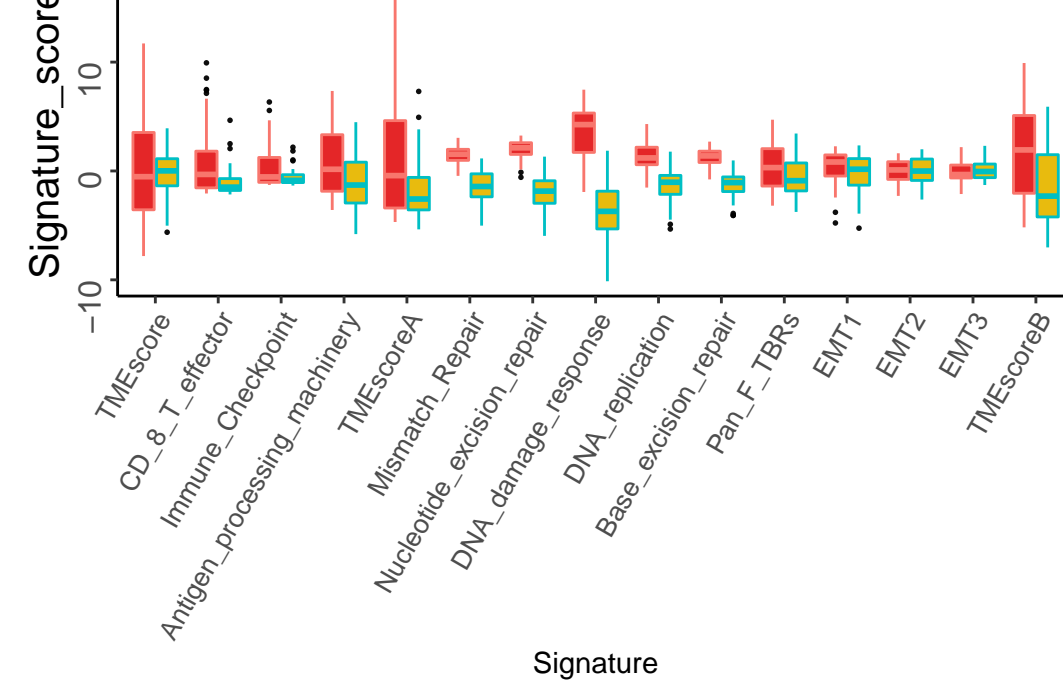

Supplement: Supplementary file 3 — Additional file 3: Supplementary Figure 3. The tumor microenvironment-based gene signature in high and low WAC-AS1 expression groups in 33 types of tumors of The Cancer Genome Atlas (TCGA) cohort. “*”, “**”, “***” and “****” indicate P values smaller than 0.05, 0.01, 0.001 and 0.0001 respectively. “ns” means no significance. [file 41065_2023_290_MOESM3_ESM.pdf]

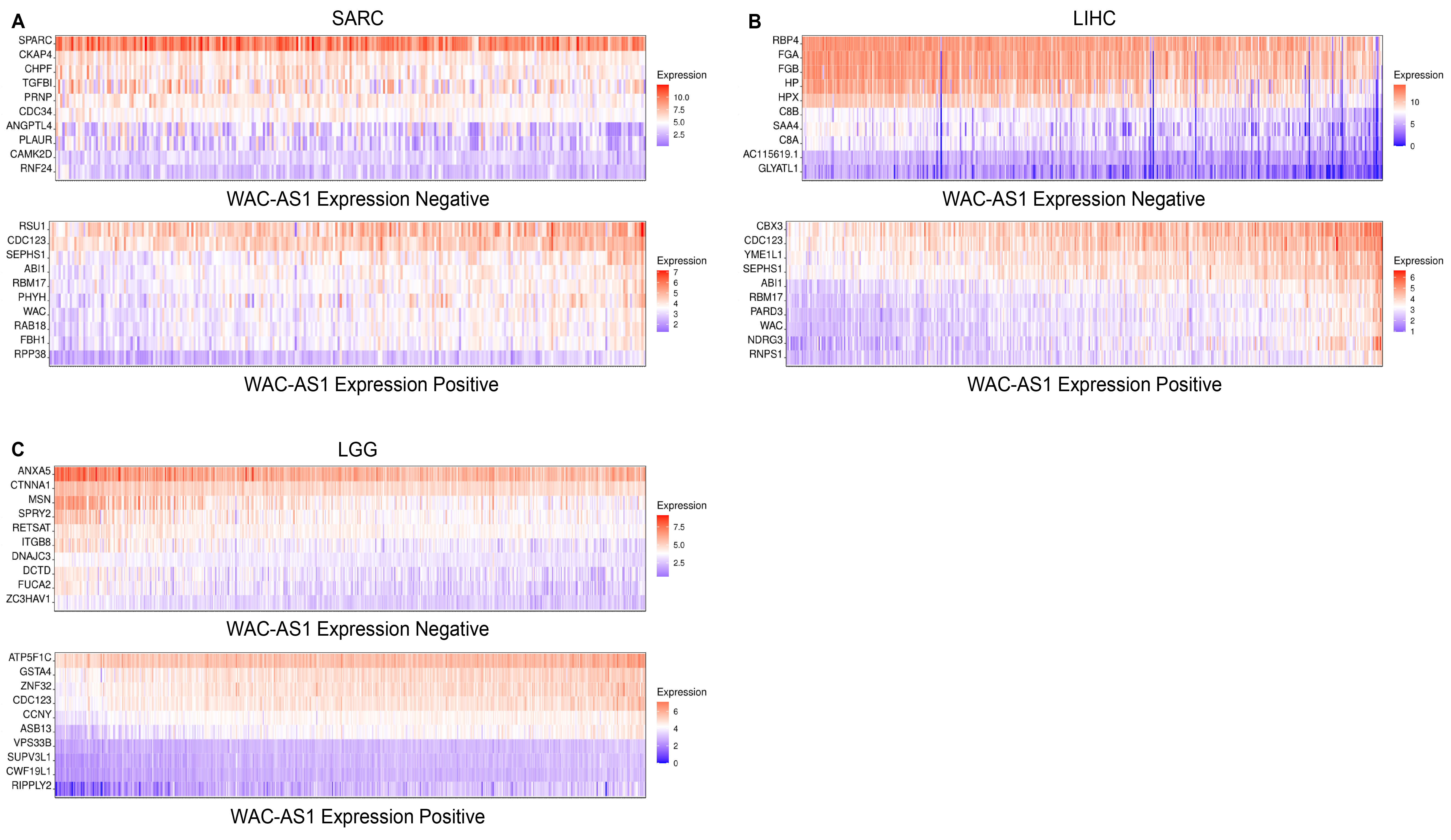

Supplement: Supplementary file 4 — Additional file 4: Supplementary Figure 4. The correlation analysis of WAC-AS1 in SARC, LIHC and LGG. Top 10 genes most positively and top 10 genes most negatively associated with WAC-AS1 were shown in heatmap. [file 41065_2023_290_MOESM4_ESM.tif]

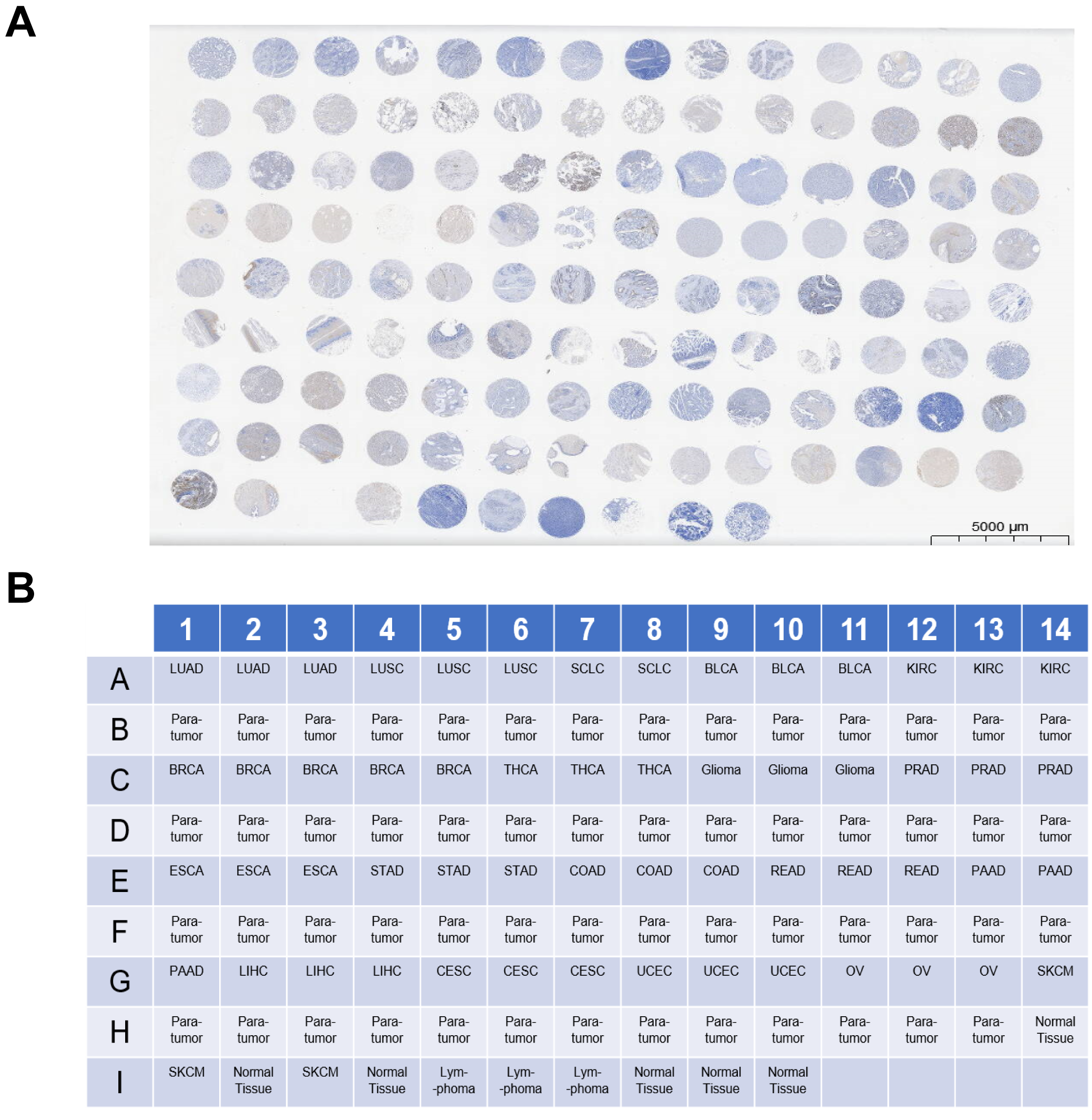

Supplement: Supplementary file 5 — Additional file 5: Supplementary Figure 5. The TMA template and the arrangement of the tissues. [file 41065_2023_290_MOESM5_ESM.tif]

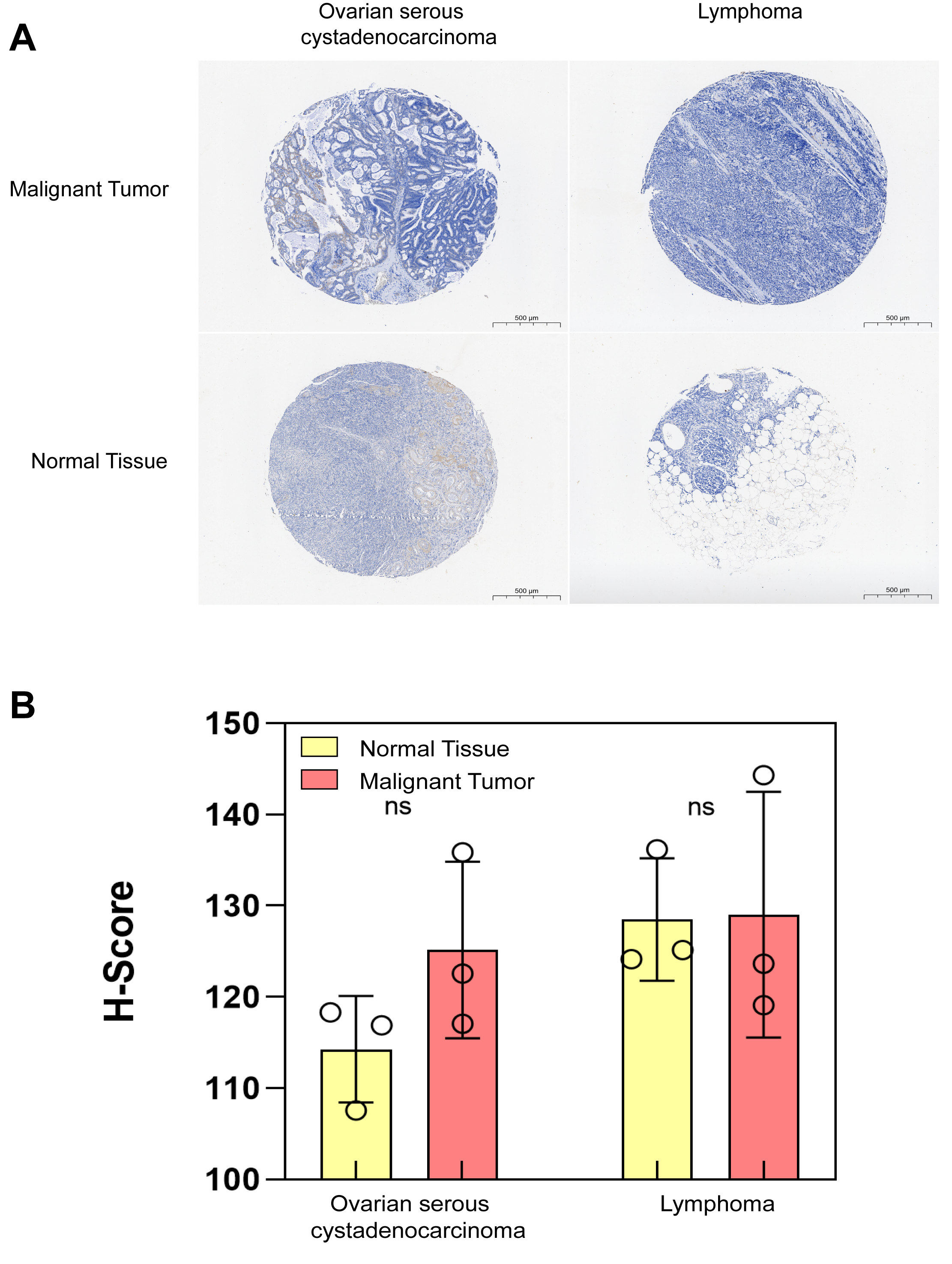

Supplement: Supplementary file 6 — Additional file 6: Supplementary Figure 6. LncRNA WAC-AS1 expression in OV and Lymphoma in TMA by ISH detection. [file 41065_2023_290_MOESM6_ESM.tif]
