# Supplementary material for: LncRNA WAC-AS1 expression in human tumors correlates with immune infiltration and affects prognosis
Source: Hereditas. 2023 May 30;160:26. doi: 10.1186/s41065-023-00290-z (PMC10227977; doi:10.1186/s41065-023-00290-z)
Supplement: Supplementary file 7 — Additional file 7: Supplementary Table 1. Correlations of WAC-AS1 expression with tumor microenvironment scores by TIMER across 33 types of tumor. [file 41065_2023_290_MOESM7_ESM.pdf]

Supplemental Table1.

Correlations of WAC-AS1 expression with tumor microenvironment scores by TIMER across 33 types of tumor.

Cancer Type	Score Name	cor	p-Value
ACC	StromalScore	-0.197007	0.081819517
ACC	ImmuneScore	-0.16910046	0.136278735
ACC	ESTIMATEScore	-0.18858382	0.096027836
ACC	TumorPurity	0.12672594	0.265749108
BLCA	StromalScore	-0.08268306	0.089429147
BLCA	ImmuneScore	-0.21816037	5.95E-06
BLCA	ESTIMATEScore	-0.1601076	0.000951374
BLCA	TumorPurity	0.169683986	0.000456595
BRCA	StromalScore	-0.16774489	1.21E-08
BRCA	ImmuneScore	0.006383601	0.829532721
BRCA	ESTIMATEScore	-0.08043793	0.006581262
BRCA	TumorPurity	0.074941867	0.01137013
CESC	StromalScore	-0.00989229	0.862280086
CESC	ImmuneScore	-0.0493225	0.386802376
CESC	ESTIMATEScore	-0.03651385	0.521841594
CESC	TumorPurity	0.027142912	0.634033479
CHOL	StromalScore	0.054946609	0.750268578
CHOL	ImmuneScore	0.033844072	0.844646247
CHOL	ESTIMATEScore	0.044571941	0.796312976
CHOL	TumorPurity	-0.02405861	0.88923107
COAD	StromalScore	-0.13699989	0.001758147
COAD	ImmuneScore	-0.29406787	8.21E-12
COAD	ESTIMATEScore	-0.22544545	2.10E-07
COAD	TumorPurity	0.18783414	1.65E-05
DLBC	StromalScore	0.084744263	0.566860842
DLBC	ImmuneScore	-0.07438526	0.615337931
DLBC	ESTIMATEScore	0.008203747	0.955867673
DLBC	TumorPurity	-0.00654089	0.96480676
ESCA	StromalScore	-0.05584115	0.477579941
ESCA	ImmuneScore	-0.23279905	0.002701516
ESCA	ESTIMATEScore	-0.15588126	0.046241033
ESCA	TumorPurity	0.146616092	0.061016294
GBM	StromalScore	-0.07013371	0.342811488
GBM	ImmuneScore	-0.1202032	0.103149336
GBM	ESTIMATEScore	-0.10200595	0.167084208
GBM	TumorPurity	0.098680919	0.181427566
HNSC	StromalScore	-0.17684144	6.34E-05
HNSC	ImmuneScore	-0.11453096	0.009925093
HNSC	ESTIMATEScore	-0.16305401	0.000230128
HNSC	TumorPurity	0.167065446	0.000159805
KICH	StromalScore	0.057328451	0.650115312
KICH	ImmuneScore	0.008307628	0.947632793
KICH	ESTIMATEScore	0.030924793	0.806809195
KICH	TumorPurity	-0.01911689	0.879858523
KIRC	StromalScore	-0.01358308	0.749945634
KIRC	ImmuneScore	0.081666468	0.054943071
KIRC	ESTIMATEScore	0.045388485	0.286655399
KIRC	TumorPurity	-0.01974886	0.643072235
KIRP	StromalScore	-0.31405759	4.41E-08
KIRP	ImmuneScore	-0.26416998	4.91E-06
KIRP	ESTIMATEScore	-0.30102998	1.65E-07
KIRP	TumorPurity	0.300399337	1.76E-07
LAML	StromalScore	0.083463804	0.308261528
LAML	ImmuneScore	0.100830503	0.217996925
LAML	ESTIMATEScore	0.099822187	0.222657949

LAML	TumorPurity	-0.10007374	0.22148855
LGG	StromalScore	-0.3858571	0
LGG	ImmuneScore	-0.30853767	4.22E-14
LGG	ESTIMATEScore	-0.34906605	0
LGG	TumorPurity	0.334047689	2.22E-16
LIHC	StromalScore	-0.20705653	4.54E-05
LIHC	ImmuneScore	-0.15453514	0.002456273
LIHC	ESTIMATEScore	-0.1926437	0.000151626
LIHC	TumorPurity	0.172403078	0.000714598
LUAD	StromalScore	-0.23311863	1.79E-08
LUAD	ImmuneScore	-0.19829157	1.83E-06
LUAD	ESTIMATEScore	-0.23215739	2.06E-08
LUAD	TumorPurity	0.225916454	4.97E-08
LUSC	StromalScore	-0.24816796	1.75E-08
LUSC	ImmuneScore	-0.17759283	6.31E-05
LUSC	ESTIMATEScore	-0.22373032	4.09E-07
LUSC	TumorPurity	0.21383348	1.33E-06
MESO	StromalScore	-0.18751309	0.083835939
MESO	ImmuneScore	0.036611737	0.737876102
MESO	ESTIMATEScore	-0.06238871	0.568230676
MESO	TumorPurity	0.056266929	0.60685319
OV	StromalScore	-0.09703855	0.05712746
OV	ImmuneScore	-0.13500023	0.007991342
OV	ESTIMATEScore	-0.12932546	0.011086118
OV	TumorPurity	0.124736124	0.014320838
PAAD	StromalScore	0.075196284	0.315737004
PAAD	ImmuneScore	0.21340699	0.004022145
PAAD	ESTIMATEScore	0.152603509	0.040842893
PAAD	TumorPurity	-0.1643988	0.027432905
PCPG	StromalScore	-0.26156031	0.000238521
PCPG	ImmuneScore	-0.31212344	9.92E-06
PCPG	ESTIMATEScore	-0.30263724	1.89E-05
PCPG	TumorPurity	0.274354188	0.000112905
PRAD	StromalScore	-0.03939433	0.376057944
PRAD	ImmuneScore	0.015238948	0.732122769
PRAD	ESTIMATEScore	-0.01075016	0.809193052
PRAD	TumorPurity	0.004724718	0.915484854
READ	StromalScore	-0.12024976	0.119398998
READ	ImmuneScore	-0.27011297	0.000382811
READ	ESTIMATEScore	-0.20272951	0.008207045
READ	TumorPurity	0.175311543	0.022618303
SARC	StromalScore	-0.18729511	0.00195845
SARC	ImmuneScore	-0.09446929	0.120795358
SARC	ESTIMATEScore	-0.14015227	0.02100273
SARC	TumorPurity	0.115735186	0.057062225
SKCM	StromalScore	-0.02294153	0.617214659
SKCM	ImmuneScore	0.075165964	0.101075369
SKCM	ESTIMATEScore	0.040786398	0.37409924
SKCM	TumorPurity	-0.05726576	0.21186766
STAD	StromalScore	-0.10103647	0.050578764
STAD	ImmuneScore	-0.19558437	0.000138011
STAD	ESTIMATEScore	-0.16254317	0.001587715
STAD	TumorPurity	0.155941196	0.002459718
TGCT	StromalScore	0.149155645	0.053649918
TGCT	ImmuneScore	0.278522112	0.000256467
TGCT	ESTIMATEScore	0.291077111	0.000129271
TGCT	TumorPurity	-0.30340992	6.39E-05
THCA	StromalScore	-0.13444648	0.00200026

THCA	ImmuneScore	-0.17596814	4.95E-05
THCA	ESTIMATEScore	-0.17181069	7.48E-05
THCA	TumorPurity	0.147771369	0.000674574
THYM	StromalScore	-0.08160096	0.377648182
THYM	ImmuneScore	0.002303114	0.980167553
THYM	ESTIMATEScore	-0.04321825	0.640716476
THYM	TumorPurity	0.062257704	0.50117912
UCEC	StromalScore	-0.14669666	0.000447138
UCEC	ImmuneScore	-0.16913034	5.02E-05
UCEC	ESTIMATEScore	-0.17837618	1.87E-05
UCEC	TumorPurity	0.175326358	2.60E-05
UCS	StromalScore	0.000288715	0.998315018
UCS	ImmuneScore	0.008117752	0.952650629
UCS	ESTIMATEScore	0.005137999	0.970020728
UCS	TumorPurity	0.014456765	0.915780738
UVM	StromalScore	-0.21064462	0.060722178
UVM	ImmuneScore	-0.02178486	0.847894354
UVM	ESTIMATEScore	-0.08692412	0.443266131
UVM	TumorPurity	0.026457037	0.815796082
