# Supplementary material for: LncRNA WAC-AS1 expression in human tumors correlates with immune infiltration and affects prognosis
Source: Hereditas. 2023 May 30;160:26. doi: 10.1186/s41065-023-00290-z (PMC10227977; doi:10.1186/s41065-023-00290-z)
Supplement: Supplementary file 8 — Additional file 8: Supplemental Table 2. Correlation of WAC-AS1 expression with immune cell infiltration in 33 types of tumor. [file 41065_2023_290_MOESM8_ESM.pdf]

Supplemental Table2.

Correlation of WAC-AS1 expression with immune cell infiltration in 33 types of tumor.

Cancer Type	Cell Type	cor	p-Value
ACC	B cells naive	0.099164824	0.532095814
ACC	B cells memory	-0.060323188	0.704326359
ACC	Plasma cells	0.073351056	0.644335242
ACC	T cells CD8	0.361479497	0.018667211
ACC	T cells CD4 naive	0	NA
ACC	T cells CD4 memory resting	-0.409167448	0.007132446
ACC	T cells CD4 memory activated	0.131594664	0.406134587
ACC	T cells follicular helper	0.29501311	0.057872941
ACC	T cells regulatory (Tregs)	0.03689013	0.816585187
ACC	T cells gamma delta	-0.090194329	0.57000392
ACC	NK cells resting	-0.028241124	0.859087941
ACC	NK cells activated	0.229561546	0.143619899
ACC	Monocytes	-0.217387085	0.166690789
ACC	Macrophages M0	0.239180886	0.127127207
ACC	Macrophages M1	0.348473685	0.023720671
ACC	Macrophages M2	-0.078726053	0.620193402
ACC	Dendritic cells resting	0.142871944	0.366729822
ACC	Dendritic cells activated	-0.23399483	0.135833077
ACC	Mast cells resting	-0.228904862	0.144800766
ACC	Mast cells activated	0.312892451	0.043637764
ACC	Eosinophils	-0.074835573	0.637629984
ACC	Neutrophils	0.454727842	0.002482839
BLCA	B cells naive	0.000571949	0.991542291
BLCA	B cells memory	0.100569618	0.061668231
BLCA	Plasma cells	0.031285652	0.561926184
BLCA	T cells CD8	-0.07180991	0.182653638
BLCA	T cells CD4 naive	0.030548613	0.571181068
BLCA	T cells CD4 memory resting	-0.062600668	0.245493365
BLCA	T cells CD4 memory activated	-0.124750178	0.020277962
BLCA	T cells follicular helper	-0.05482526	0.309208917
BLCA	T cells regulatory (Tregs)	0.036902092	0.493869285
BLCA	T cells gamma delta	0.037331591	0.488851826
BLCA	NK cells resting	0.105428619	0.050059756
BLCA	NK cells activated	-0.102808993	0.056068516
BLCA	Monocytes	0.039385418	0.465241425
BLCA	Macrophages M0	0.124187828	0.020853893
BLCA	Macrophages M1	-0.06345665	0.239084835
BLCA	Macrophages M2	0.188377938	0.000426782
BLCA	Dendritic cells resting	-0.153889815	0.004114232
BLCA	Dendritic cells activated	0.045529685	0.398516147
BLCA	Mast cells resting	0.046319248	0.390378552
BLCA	Mast cells activated	-0.023616676	0.661555241
BLCA	Eosinophils	-0.005733761	0.915368899
BLCA	Neutrophils	-0.123021579	0.022093703
BRCA	B cells naive	0.047232967	0.118610569
BRCA	B cells memory	0.037905072	0.210504784
BRCA	Plasma cells	-0.02358516	0.436006514
BRCA	T cells CD8	0.079249409	0.008763253
BRCA	T cells CD4 naive	0	NA
BRCA	T cells CD4 memory resting	-0.110907862	0.000238952
BRCA	T cells CD4 memory activated	0.087356498	0.003849037
BRCA	T cells follicular helper	0.128098119	2.16E-05

BRCA	T cells regulatory (Tregs)	-0.005349857	0.859769106
BRCA	T cells gamma delta	-0.009576468	0.751814058
BRCA	NK cells resting	-0.002420297	0.936296982
BRCA	NK cells activated	0.082533857	0.006330522
BRCA	Monocytes	-0.027192529	0.369112304
BRCA	Macrophages M0	0.048714098	0.107479143
BRCA	Macrophages M1	0.106581916	0.000416058
BRCA	Macrophages M2	-0.047867841	0.113733364
BRCA	Dendritic cells resting	-0.04991577	0.099069241
BRCA	Dendritic cells activated	0.097581732	0.001237357
BRCA	Mast cells resting	-0.178234271	2.97E-09
BRCA	Mast cells activated	0.026800159	0.376063242
BRCA	Eosinophils	-0.032248974	0.286773695
BRCA	Neutrophils	-0.044599086	0.140611844
CESC	B cells naive	-0.046848957	0.42273732
CESC	B cells memory	0.053445972	0.360337916
CESC	Plasma cells	-0.072344303	0.215381496
CESC	T cells CD8	0.018467492	0.752101274
CESC	T cells CD4 naive	0	NA
CESC	T cells CD4 memory resting	0.036266864	0.534952731
CESC	T cells CD4 memory activated	0.051811241	0.375237793
CESC	T cells follicular helper	-0.059915742	0.305061631
CESC	T cells regulatory (Tregs)	0.029131689	0.618248166
CESC	T cells gamma delta	-0.00901058	0.877524684
CESC	NK cells resting	0.061417477	0.293077623
CESC	NK cells activated	-0.056928156	0.3298518
CESC	Monocytes	-0.051206448	0.380844837
CESC	Macrophages M0	0.03529777	0.545927812
CESC	Macrophages M1	0.093709212	0.108227435
CESC	Macrophages M2	0.062061731	0.288034088
CESC	Dendritic cells resting	0.011493897	0.844154246
CESC	Dendritic cells activated	-0.051192874	0.380971273
CESC	Mast cells resting	-0.000119958	0.998363059
CESC	Mast cells activated	-0.078506264	0.17870889
CESC	Eosinophils	0.029637655	0.612159274
CESC	Neutrophils	-0.109679692	0.059904922
CHOL	B cells naive	-0.050203678	0.784954774
CHOL	B cells memory	0.322229127	0.072082083
CHOL	Plasma cells	-0.233637181	0.198105844
CHOL	T cells CD8	0.150746181	0.410199043
CHOL	T cells CD4 naive	0	NA
CHOL	T cells CD4 memory resting	-0.036001992	0.844908786
CHOL	T cells CD4 memory activated	-0.033975501	0.853542086
CHOL	T cells follicular helper	0.076881811	0.675781948
CHOL	T cells regulatory (Tregs)	0.023944868	0.896501583
CHOL	T cells gamma delta	0.059394556	0.746767916
CHOL	NK cells resting	-0.335644855	0.060374578
CHOL	NK cells activated	0.523767814	0.002094052
CHOL	Monocytes	0.140645363	0.442615225
CHOL	Macrophages M0	-0.365998287	0.039386839
CHOL	Macrophages M1	0.112847778	0.538597704
CHOL	Macrophages M2	-0.092764133	0.613582359
CHOL	Dendritic cells resting	-0.064163342	0.727178135
CHOL	Dendritic cells activated	-0.02310559	0.900110876
CHOL	Mast cells resting	0.306620849	0.087842288

CHOL	Mast cells activated	-0.217431191	0.231927937
CHOL	Eosinophils	-0.047553472	0.796062244
CHOL	Neutrophils	-0.317664207	0.076444524
COAD	B cells naive	0.096818514	0.039856212
COAD	B cells memory	0.034398314	0.466186931
COAD	Plasma cells	-0.034226232	0.468421593
COAD	T cells CD8	0.024836816	0.598838947
COAD	T cells CD4 naive	0	NA
COAD	T cells CD4 memory resting	0.043250592	0.359466397
COAD	T cells CD4 memory activated	0.048472416	0.304352561
COAD	T cells follicular helper	0.009350766	0.843019074
COAD	T cells regulatory (Tregs)	0.055671243	0.238035584
COAD	T cells gamma delta	-0.048559651	0.303482056
COAD	NK cells resting	-0.090893211	0.053741429
COAD	NK cells activated	-0.081504836	0.083813981
COAD	Monocytes	-0.028931566	0.539984818
COAD	Macrophages M0	0.122642524	0.009129915
COAD	Macrophages M1	-0.019040941	0.686740571
COAD	Macrophages M2	-0.068909743	0.143989168
COAD	Dendritic cells resting	-0.000290594	0.995089735
COAD	Dendritic cells activated	-0.119663831	0.01097906
COAD	Mast cells resting	0.104624111	0.026296021
COAD	Mast cells activated	-0.168149411	0.000335007
COAD	Eosinophils	-0.119534007	0.011066709
COAD	Neutrophils	-0.208851875	7.73E-06
DLBC	B cells naive	0.095659659	0.517783764
DLBC	B cells memory	0.073291382	0.620558189
DLBC	Plasma cells	-0.068833671	0.642021022
DLBC	T cells CD8	-0.171734792	0.243151343
DLBC	T cells CD4 naive	0	NA
DLBC	T cells CD4 memory resting	0.148793923	0.312814701
DLBC	T cells CD4 memory activated	-0.306080386	0.034365165
DLBC	T cells follicular helper	0.017528925	0.905868199
DLBC	T cells regulatory (Tregs)	0.463528154	0.000906634
DLBC	T cells gamma delta	-0.050856487	0.731388842
DLBC	NK cells resting	-0.081227842	0.583117319
DLBC	NK cells activated	-0.065341625	0.659039924
DLBC	Monocytes	0.182344166	0.214809865
DLBC	Macrophages M0	0.070295347	0.634950361
DLBC	Macrophages M1	-0.094266588	0.523926732
DLBC	Macrophages M2	-0.20620978	0.159673993
DLBC	Dendritic cells resting	0.068362	0.64430942
DLBC	Dendritic cells activated	-0.111508396	0.450521223
DLBC	Mast cells resting	0.073850376	0.617888196
DLBC	Mast cells activated	0	NA
DLBC	Eosinophils	-0.343015936	0.016997972
DLBC	Neutrophils	-0.128525401	0.383974354
ESCA	B cells naive	0.133975261	0.10450669
ESCA	B cells memory	-0.185075268	0.024326173
ESCA	Plasma cells	0.068721211	0.406582494
ESCA	T cells CD8	-0.096343492	0.244082492
ESCA	T cells CD4 naive	0	NA
ESCA	T cells CD4 memory resting	0.072826717	0.379058757
ESCA	T cells CD4 memory activated	-0.099219819	0.230221062
ESCA	T cells follicular helper	0.121561183	0.141080206

ESCA	T cells regulatory (Tregs)	-0.023651447	0.775389397
ESCA	T cells gamma delta	-0.068917499	0.405240282
ESCA	NK cells resting	-0.06360352	0.44249461
ESCA	NK cells activated	-0.04870545	0.556631363
ESCA	Monocytes	0.087999013	0.287536317
ESCA	Macrophages M0	0.12747811	0.122589559
ESCA	Macrophages M1	0.009546826	0.908318299
ESCA	Macrophages M2	-0.062435234	0.450937467
ESCA	Dendritic cells resting	0.069524957	0.401103144
ESCA	Dendritic cells activated	-0.132406795	0.108667404
ESCA	Mast cells resting	0.01813553	0.826824899
ESCA	Mast cells activated	-0.245130063	0.002675061
ESCA	Eosinophils	-0.040918274	0.621462682
ESCA	Neutrophils	-0.154459368	0.060873709
GBM	B cells naive	-0.041702466	0.602885148
GBM	B cells memory	0.121413649	0.128597599
GBM	Plasma cells	-0.086594179	0.279313531
GBM	T cells CD8	0.053504196	0.504340683
GBM	T cells CD4 naive	-0.005366248	0.946647327
GBM	T cells CD4 memory resting	-0.171563199	0.031130561
GBM	T cells CD4 memory activated	-0.058294905	0.466885243
GBM	T cells follicular helper	-0.008580644	0.914786944
GBM	T cells regulatory (Tregs)	0.039334777	0.623642529
GBM	T cells gamma delta	-0.175726562	0.027207723
GBM	NK cells resting	-0.03537805	0.658994123
GBM	NK cells activated	-0.173897845	0.028875451
GBM	Monocytes	0.196987155	0.013109292
GBM	Macrophages M0	0.045847933	0.567305375
GBM	Macrophages M1	-0.010726687	0.89358793
GBM	Macrophages M2	0.030336391	0.705146986
GBM	Dendritic cells resting	0.041575252	0.603992564
GBM	Dendritic cells activated	-0.147194248	0.064953556
GBM	Mast cells resting	-0.096480924	0.227843599
GBM	Mast cells activated	-0.008659717	0.914004682
GBM	Eosinophils	0.067838119	0.397041224
GBM	Neutrophils	-0.117442835	0.141669456
HNSC	B cells naive	0.07788843	0.081566047
HNSC	B cells memory	0.024173367	0.589334601
HNSC	Plasma cells	0.068033989	0.128318717
HNSC	T cells CD8	0.156453448	0.000440048
HNSC	T cells CD4 naive	0.036491884	0.415055064
HNSC	T cells CD4 memory resting	-0.171933004	0.000109909
HNSC	T cells CD4 memory activated	0.029785602	0.505939032
HNSC	T cells follicular helper	0.195759432	1.02E-05
HNSC	T cells regulatory (Tregs)	0.103536268	0.02045481
HNSC	T cells gamma delta	0.002290043	0.95922182
HNSC	NK cells resting	-0.022693671	0.612330556
HNSC	NK cells activated	-0.08629453	0.053568398
HNSC	Monocytes	-0.045262232	0.311971686
HNSC	Macrophages M0	-0.010632102	0.812353537
HNSC	Macrophages M1	-0.063785543	0.153984003
HNSC	Macrophages M2	0.039945412	0.372273895
HNSC	Dendritic cells resting	0.016797476	0.707611592
HNSC	Dendritic cells activated	-0.042842771	0.338569727
HNSC	Mast cells resting	-0.050993611	0.254585657

HNSC	Mast cells activated	-0.066823152	0.135270175
HNSC	Eosinophils	-0.090110248	0.043799445
HNSC	Neutrophils	-0.166500129	0.00018138
KICH	B cells naive	-0.090335326	0.600308616
KICH	B cells memory	0.038176902	0.82504737
KICH	Plasma cells	0.153544238	0.371284176
KICH	T cells CD8	0.452104671	0.00563635
KICH	T cells CD4 naive	0	NA
KICH	T cells CD4 memory resting	-0.110056632	0.522835368
KICH	T cells CD4 memory activated	0.285312706	0.091656331
KICH	T cells follicular helper	-0.14809683	0.38869361
KICH	T cells regulatory (Tregs)	-0.162885622	0.342527943
KICH	T cells gamma delta	-0.121949441	0.478619271
KICH	NK cells resting	-0.17677671	0.302363278
KICH	NK cells activated	-0.267405339	0.114870432
KICH	Monocytes	0.669060043	8.16E-06
KICH	Macrophages M0	-0.301478142	0.073959301
KICH	Macrophages M1	-0.055866527	0.746224025
KICH	Macrophages M2	-0.176326241	0.303616736
KICH	Dendritic cells resting	-0.157460051	0.359060126
KICH	Dendritic cells activated	-0.253408283	0.135894922
KICH	Mast cells resting	0.046513486	0.787639273
KICH	Mast cells activated	0.237812986	0.162518401
KICH	Eosinophils	0.086582231	0.615594227
KICH	Neutrophils	0.11442498	0.506365088
KIRC	B cells naive	-0.082366144	0.05857643
KIRC	B cells memory	0.038935742	0.371914235
KIRC	Plasma cells	-0.047865826	0.272250074
KIRC	T cells CD8	0.137893108	0.001492085
KIRC	T cells CD4 naive	0	NA
KIRC	T cells CD4 memory resting	0.005599695	0.897858345
KIRC	T cells CD4 memory activated	-0.01851403	0.671239003
KIRC	T cells follicular helper	0.096855461	0.026045744
KIRC	T cells regulatory (Tregs)	-0.003265914	0.940320259
KIRC	T cells gamma delta	-0.015056746	0.729961091
KIRC	NK cells resting	0.047006245	0.280963503
KIRC	NK cells activated	0.081564064	0.061085078
KIRC	Monocytes	0.023360677	0.592243047
KIRC	Macrophages M0	-0.142430483	0.001031493
KIRC	Macrophages M1	0.069135328	0.112570894
KIRC	Macrophages M2	-0.061293951	0.159601701
KIRC	Dendritic cells resting	-0.042839468	0.325856471
KIRC	Dendritic cells activated	-0.091880398	0.034796942
KIRC	Mast cells resting	0.017190816	0.693501447
KIRC	Mast cells activated	0.022595201	0.604434664
KIRC	Eosinophils	-0.031060257	0.47634668
KIRC	Neutrophils	-0.094701429	0.029569634
KIRP	B cells naive	-0.222166045	0.00029771
KIRP	B cells memory	0.158990095	0.010093219
KIRP	Plasma cells	-0.047920204	0.440765225
KIRP	T cells CD8	-0.150140785	0.015193515
KIRP	T cells CD4 naive	0	NA
KIRP	T cells CD4 memory resting	0.297469859	9.88E-07
KIRP	T cells CD4 memory activated	-0.120918773	0.051022366
KIRP	T cells follicular helper	-0.026542838	0.669503417

KIRP	T cells regulatory (Tregs)	-0.077243004	0.213584871
KIRP	T cells gamma delta	-0.119884893	0.053053431
KIRP	NK cells resting	0.030405543	0.6248616
KIRP	NK cells activated	0.027433076	0.659103924
KIRP	Monocytes	0.083104325	0.180745361
KIRP	Macrophages M0	-0.259716929	2.15E-05
KIRP	Macrophages M1	-0.186090488	0.002541449
KIRP	Macrophages M2	-0.084171694	0.175189758
KIRP	Dendritic cells resting	0.053275333	0.391352558
KIRP	Dendritic cells activated	0.101817108	0.100739644
KIRP	Mast cells resting	0.339720921	1.80E-08
KIRP	Mast cells activated	0.130953347	0.034466127
KIRP	Eosinophils	-0.13795837	0.025830182
KIRP	Neutrophils	-0.008257203	0.894382274
LAML	B cells naive	-0.201832526	0.012948562
LAML	B cells memory	0.029703396	0.71731598
LAML	Plasma cells	-0.141532613	0.083012768
LAML	T cells CD8	-0.201971466	0.012885155
LAML	T cells CD4 naive	0.034748507	0.671874707
LAML	T cells CD4 memory resting	0.12083953	0.13941225
LAML	T cells CD4 memory activated	-0.032340665	0.693426376
LAML	T cells follicular helper	0.18693343	0.021545528
LAML	T cells regulatory (Tregs)	-0.116570774	0.154039696
LAML	T cells gamma delta	5.29E-06	0.9999486
LAML	NK cells resting	-0.140354845	0.085633247
LAML	NK cells activated	-0.07459607	0.362662355
LAML	Monocytes	0.214200791	0.008266246
LAML	Macrophages M0	0.121982992	0.135679788
LAML	Macrophages M1	-0.016489602	0.840731823
LAML	Macrophages M2	0.125422338	0.12491306
LAML	Dendritic cells resting	-0.059767055	0.46601458
LAML	Dendritic cells activated	0.041113442	0.616213325
LAML	Mast cells resting	-0.20731283	0.010644911
LAML	Mast cells activated	-0.082531326	0.313716522
LAML	Eosinophils	0.056555682	0.490354486
LAML	Neutrophils	0.293396111	0.000256031
LGG	B cells naive	-0.086254806	0.103239412
LGG	B cells memory	0.033108269	0.532352939
LGG	Plasma cells	-0.029042928	0.583889844
LGG	T cells CD8	-0.049904174	0.346440577
LGG	T cells CD4 naive	0.097039123	0.06665762
LGG	T cells CD4 memory resting	-0.287190524	3.17E-08
LGG	T cells CD4 memory activated	-0.074580825	0.159083063
LGG	T cells follicular helper	-0.014236364	0.788363561
LGG	T cells regulatory (Tregs)	-0.105072636	0.046964851
LGG	T cells gamma delta	-0.038203481	0.471168627
LGG	NK cells resting	0.123194811	0.019716908
LGG	NK cells activated	0.109584667	0.038225066
LGG	Monocytes	0.086261804	0.103211342
LGG	Macrophages M0	-0.08415349	0.111945098
LGG	Macrophages M1	-0.170489778	0.001202543
LGG	Macrophages M2	0.143326127	0.006599378
LGG	Dendritic cells resting	0.058770476	0.267404499
LGG	Dendritic cells activated	-0.123295035	0.0196165
LGG	Mast cells resting	-0.062545134	0.237830506

LGG	Mast cells activated	0.090172892	0.088445261
LGG	Eosinophils	0.009540839	0.857235854
LGG	Neutrophils	-0.057344869	0.279202784
LIHC	B cells naive	-0.010695002	0.8546172
LIHC	B cells memory	0.122919825	0.034526627
LIHC	Plasma cells	0.077838656	0.181697176
LIHC	T cells CD8	-0.123612181	0.033514465
LIHC	T cells CD4 naive	0.01691316	0.771988735
LIHC	T cells CD4 memory resting	0.009089166	0.876255713
LIHC	T cells CD4 memory activated	0.008618059	0.882622097
LIHC	T cells follicular helper	0.098262403	0.091506622
LIHC	T cells regulatory (Tregs)	0.034486702	0.554522439
LIHC	T cells gamma delta	-0.04953648	0.395784916
LIHC	NK cells resting	-0.043635025	0.454517352
LIHC	NK cells activated	-0.105929287	0.06877597
LIHC	Monocytes	0.118868244	0.040984716
LIHC	Macrophages M0	0.218535997	0.000150747
LIHC	Macrophages M1	-0.053896263	0.35547866
LIHC	Macrophages M2	-0.132643416	0.022458519
LIHC	Dendritic cells resting	0.041497058	0.476942979
LIHC	Dendritic cells activated	0.223034412	0.000108902
LIHC	Mast cells resting	-0.088793751	0.127455331
LIHC	Mast cells activated	-0.073108457	0.209783004
LIHC	Eosinophils	-0.007493871	0.897843747
LIHC	Neutrophils	0.031262734	0.592152376
LUAD	B cells naive	0.14425192	0.000828402
LUAD	B cells memory	-0.022632095	0.601786132
LUAD	Plasma cells	0.14131363	0.001059125
LUAD	T cells CD8	0.082701602	0.056146169
LUAD	T cells CD4 naive	0	NA
LUAD	T cells CD4 memory resting	-0.039803327	0.358618229
LUAD	T cells CD4 memory activated	-0.063977739	0.139816644
LUAD	T cells follicular helper	0.177342429	3.77E-05
LUAD	T cells regulatory (Tregs)	0.09212746	0.033298854
LUAD	T cells gamma delta	-0.082781683	0.055907609
LUAD	NK cells resting	0.027328364	0.528594027
LUAD	NK cells activated	0.000310817	0.994282669
LUAD	Monocytes	-0.116460464	0.007058195
LUAD	Macrophages M0	0.082713328	0.056111186
LUAD	Macrophages M1	0.043545367	0.315193588
LUAD	Macrophages M2	-0.123105048	0.004386474
LUAD	Dendritic cells resting	-0.14010731	0.00116998
LUAD	Dendritic cells activated	0.019825021	0.647601559
LUAD	Mast cells resting	-0.098684696	0.022567691
LUAD	Mast cells activated	-0.012857786	0.766894563
LUAD	Eosinophils	-0.127532865	0.003154744
LUAD	Neutrophils	-0.211229369	8.42E-07
LUSC	B cells naive	0.126866118	0.004575629
LUSC	B cells memory	0.002735303	0.951448806
LUSC	Plasma cells	-0.135570629	0.002431097
LUSC	T cells CD8	0.14447447	0.001225169
LUSC	T cells CD4 naive	-0.044165016	0.325319129
LUSC	T cells CD4 memory resting	-0.138359253	0.001969781
LUSC	T cells CD4 memory activated	-0.005799245	0.897285385
LUSC	T cells follicular helper	0.113284344	0.011411477

LUSC	T cells regulatory (Tregs)	0.088364894	0.048741646
LUSC	T cells gamma delta	-0.046620865	0.299112996
LUSC	NK cells resting	-0.011126957	0.804372802
LUSC	NK cells activated	0.082355905	0.066306009
LUSC	Monocytes	0.008638626	0.847508796
LUSC	Macrophages M0	0.081776587	0.068244609
LUSC	Macrophages M1	0.100065337	0.025545967
LUSC	Macrophages M2	-0.173376764	0.00010073
LUSC	Dendritic cells resting	-0.042463969	0.344318177
LUSC	Dendritic cells activated	0.052985777	0.237887329
LUSC	Mast cells resting	0.048931716	0.27577268
LUSC	Mast cells activated	-0.055432863	0.216876645
LUSC	Eosinophils	-0.023660638	0.598363294
LUSC	Neutrophils	-0.188656933	2.26E-05
MESO	B cells naive	0.036939472	0.740233595
MESO	B cells memory	-0.041874785	0.707008543
MESO	Plasma cells	0.257992421	0.018533144
MESO	T cells CD8	0.184596791	0.094792291
MESO	T cells CD4 naive	0	NA
MESO	T cells CD4 memory resting	-0.218377353	0.047328594
MESO	T cells CD4 memory activated	-0.178638607	0.106133345
MESO	T cells follicular helper	0.395044516	0.000219045
MESO	T cells regulatory (Tregs)	-0.104327309	0.347927334
MESO	T cells gamma delta	-0.020921812	0.851083118
MESO	NK cells resting	-0.117099376	0.291755118
MESO	NK cells activated	0.201940796	0.067133713
MESO	Monocytes	-0.013325551	0.904827192
MESO	Macrophages M0	0.048283198	0.664678623
MESO	Macrophages M1	0.003881606	0.972217743
MESO	Macrophages M2	-0.261520338	0.01693334
MESO	Dendritic cells resting	0.14686013	0.185216047
MESO	Dendritic cells activated	-0.016246072	0.884100037
MESO	Mast cells resting	-0.161515867	0.144631059
MESO	Mast cells activated	-0.021475472	0.847189315
MESO	Eosinophils	-0.110135047	0.321597491
MESO	Neutrophils	-0.126213145	0.255550108
OV	B cells naive	0.129416069	0.023338482
OV	B cells memory	-0.102538741	0.072808953
OV	Plasma cells	0.045768384	0.424247273
OV	T cells CD8	-0.021051011	0.713335893
OV	T cells CD4 naive	0	NA
OV	T cells CD4 memory resting	-0.011013471	0.847593188
OV	T cells CD4 memory activated	0.0651185	0.25532028
OV	T cells follicular helper	0.097187282	0.089142473
OV	T cells regulatory (Tregs)	0.018781878	0.743084388
OV	T cells gamma delta	0.017316051	0.762509731
OV	NK cells resting	-0.073285781	0.200349992
OV	NK cells activated	0.015400796	0.78811521
OV	Monocytes	-0.127251298	0.025774081
OV	Macrophages M0	0.106346879	0.062740296
OV	Macrophages M1	0.133864293	0.018950953
OV	Macrophages M2	-0.089613657	0.117136428
OV	Dendritic cells resting	-0.135994752	0.017116902
OV	Dendritic cells activated	0.007869002	0.890781227
OV	Mast cells resting	0.077579433	0.17516262

OV	Mast cells activated	-0.025374823	0.657866671
OV	Eosinophils	-0.038170158	0.505213083
OV	Neutrophils	-0.071348539	0.212537951
PAAD	B cells naive	0.169538709	0.026635492
PAAD	B cells memory	-0.093144332	0.225618594
PAAD	Plasma cells	0.097942276	0.202509683
PAAD	T cells CD8	0.204668654	0.007247649
PAAD	T cells CD4 naive	0	NA
PAAD	T cells CD4 memory resting	-0.143622249	0.060922551
PAAD	T cells CD4 memory activated	0.083535428	0.27736613
PAAD	T cells follicular helper	0.096183856	0.210771993
PAAD	T cells regulatory (Tregs)	-0.092483213	0.228943494
PAAD	T cells gamma delta	-0.008404555	0.913123462
PAAD	NK cells resting	-0.088930436	0.247403081
PAAD	NK cells activated	-0.063382563	0.410176048
PAAD	Monocytes	0.136861914	0.074263931
PAAD	Macrophages M0	-0.182816349	0.016697351
PAAD	Macrophages M1	0.046880522	0.542603425
PAAD	Macrophages M2	-0.101082564	0.18834092
PAAD	Dendritic cells resting	0.048172255	0.531526811
PAAD	Dendritic cells activated	-0.102090334	0.1839513
PAAD	Mast cells resting	-0.042012832	0.585340593
PAAD	Mast cells activated	0.017297029	0.822332637
PAAD	Eosinophils	-0.020146733	0.79367076
PAAD	Neutrophils	-0.114810438	0.134841786
PCPG	B cells naive	-0.107503404	0.368737639
PCPG	B cells memory	0.035923541	0.764495069
PCPG	Plasma cells	0.00828136	0.944956978
PCPG	T cells CD8	0.127658439	0.285232154
PCPG	T cells CD4 naive	0	NA
PCPG	T cells CD4 memory resting	-0.169240734	0.1552595
PCPG	T cells CD4 memory activated	-9.52E-05	0.999366629
PCPG	T cells follicular helper	0.065726086	0.583322966
PCPG	T cells regulatory (Tregs)	0.023153822	0.846916613
PCPG	T cells gamma delta	0.016213537	0.892471548
PCPG	NK cells resting	-0.069062389	0.564313316
PCPG	NK cells activated	-0.01657088	0.8901162
PCPG	Monocytes	-0.258115597	0.028590145
PCPG	Macrophages M0	0.276503131	0.018713793
PCPG	Macrophages M1	0.073845147	0.537582344
PCPG	Macrophages M2	-0.008989547	0.940258204
PCPG	Dendritic cells resting	-0.176600347	0.137817261
PCPG	Dendritic cells activated	-0.023161726	0.846864985
PCPG	Mast cells resting	0.325744295	0.005234268
PCPG	Mast cells activated	-0.145454828	0.22279854
PCPG	Eosinophils	0.103679154	0.386112493
PCPG	Neutrophils	-0.091909169	0.44257423
PRAD	B cells naive	0.048082148	0.363680156
PRAD	B cells memory	0.085408099	0.106189042
PRAD	Plasma cells	-0.022492986	0.671019878
PRAD	T cells CD8	0.102776403	0.051690933
PRAD	T cells CD4 naive	0	NA
PRAD	T cells CD4 memory resting	-0.129434929	0.01411901
PRAD	T cells CD4 memory activated	-0.005376871	0.91913561
PRAD	T cells follicular helper	0.0323643	0.541042347

PRAD	T cells regulatory (Tregs)	0.211798544	5.23E-05
PRAD	T cells gamma delta	0.093740645	0.076088755
PRAD	NK cells resting	-0.091798805	0.082397347
PRAD	NK cells activated	0.109965546	0.037287418
PRAD	Monocytes	-0.141581171	0.007214623
PRAD	Macrophages M0	0.077155461	0.144574857
PRAD	Macrophages M1	-0.029410777	0.578599595
PRAD	Macrophages M2	0.072822121	0.168574451
PRAD	Dendritic cells resting	-0.132971074	0.01167349
PRAD	Dendritic cells activated	-0.052056923	0.325329404
PRAD	Mast cells resting	-0.042496456	0.422120686
PRAD	Mast cells activated	-0.06761629	0.201199702
PRAD	Eosinophils	0.002957112	0.95547405
PRAD	Neutrophils	-0.144613919	0.006052381
READ	B cells naive	0.014529343	0.858523796
READ	B cells memory	0.093581255	0.249909056
READ	Plasma cells	-0.032967489	0.685807474
READ	T cells CD8	0.051882101	0.524181862
READ	T cells CD4 naive	0	NA
READ	T cells CD4 memory resting	0.041086765	0.614078366
READ	T cells CD4 memory activated	-0.045239077	0.578708689
READ	T cells follicular helper	0.121264535	0.135391483
READ	T cells regulatory (Tregs)	-0.159507128	0.048902345
READ	T cells gamma delta	0.175168054	0.030333058
READ	NK cells resting	-0.149909249	0.064380721
READ	NK cells activated	-0.093527501	0.250182392
READ	Monocytes	-0.01407849	0.862870139
READ	Macrophages M0	0.14003185	0.084273581
READ	Macrophages M1	0.188367445	0.019712117
READ	Macrophages M2	0.047351608	0.561085482
READ	Dendritic cells resting	-0.152188245	0.060383714
READ	Dendritic cells activated	-0.073511853	0.366495605
READ	Mast cells resting	0.018794242	0.81763657
READ	Mast cells activated	-0.055907606	0.492457852
READ	Eosinophils	-0.168354714	0.037504701
READ	Neutrophils	-0.149981178	0.064251345
SARC	B cells naive	0.040417288	0.534063918
SARC	B cells memory	-0.03601251	0.579578309
SARC	Plasma cells	0.027275069	0.674829034
SARC	T cells CD8	0.07730604	0.233795236
SARC	T cells CD4 naive	0	NA
SARC	T cells CD4 memory resting	-0.016303059	0.802019411
SARC	T cells CD4 memory activated	0.025729283	0.692292998
SARC	T cells follicular helper	0.078060779	0.22924774
SARC	T cells regulatory (Tregs)	0.005670607	0.93050734
SARC	T cells gamma delta	0.003706253	0.954547518
SARC	NK cells resting	0.072769582	0.262467242
SARC	NK cells activated	0.035961049	0.580120565
SARC	Monocytes	-0.073019915	0.260824769
SARC	Macrophages M0	-0.021959715	0.735551718
SARC	Macrophages M1	-0.041757119	0.520584143
SARC	Macrophages M2	-0.021593483	0.739801893
SARC	Dendritic cells resting	-0.032203441	0.620337904
SARC	Dendritic cells activated	0.023413965	0.718755332
SARC	Mast cells resting	0.004343075	0.946748395

SARC	Mast cells activated	-0.00540819	0.93371568
SARC	Eosinophils	-0.108509228	0.094196957
SARC	Neutrophils	-0.064080623	0.323896223
SKCM	B cells naive	0.085511089	0.078610511
SKCM	B cells memory	0.072167943	0.137921178
SKCM	Plasma cells	0.017942326	0.712579412
SKCM	T cells CD8	0.000516931	0.991532343
SKCM	T cells CD4 naive	0	NA
SKCM	T cells CD4 memory resting	0.136617764	0.004831607
SKCM	T cells CD4 memory activated	0.180640363	0.000184431
SKCM	T cells follicular helper	-0.001644841	0.973061046
SKCM	T cells regulatory (Tregs)	-0.135831336	0.005083772
SKCM	T cells gamma delta	0.157996711	0.001097454
SKCM	NK cells resting	-0.092592604	0.056771729
SKCM	NK cells activated	-0.044121385	0.364790703
SKCM	Monocytes	-0.109856705	0.023681276
SKCM	Macrophages M0	-0.112331557	0.020692599
SKCM	Macrophages M1	0.226000606	2.59E-06
SKCM	Macrophages M2	-0.139896613	0.003897384
SKCM	Dendritic cells resting	-0.034471524	0.47899053
SKCM	Dendritic cells activated	-0.010908097	0.822792279
SKCM	Mast cells resting	-0.022341513	0.646421543
SKCM	Mast cells activated	-0.01634286	0.737210958
SKCM	Eosinophils	-0.079668978	0.101370251
SKCM	Neutrophils	-0.00954514	0.844633094
STAD	B cells naive	0.17728022	0.000602814
STAD	B cells memory	-0.060141857	0.247864798
STAD	Plasma cells	-0.00880115	0.86583334
STAD	T cells CD8	-0.053815568	0.301223843
STAD	T cells CD4 naive	0	NA
STAD	T cells CD4 memory resting	0.049537631	0.341337108
STAD	T cells CD4 memory activated	-0.166641144	0.001275405
STAD	T cells follicular helper	0.064100881	0.218034687
STAD	T cells regulatory (Tregs)	0.09346218	0.072165683
STAD	T cells gamma delta	-0.032833461	0.528398224
STAD	NK cells resting	-0.07047985	0.175531695
STAD	NK cells activated	0.106757668	0.039856234
STAD	Monocytes	-0.07710586	0.138244624
STAD	Macrophages M0	0.153030794	0.003126093
STAD	Macrophages M1	0.045814032	0.378900007
STAD	Macrophages M2	-0.079196792	0.127838062
STAD	Dendritic cells resting	-0.014247087	0.784464179
STAD	Dendritic cells activated	-0.092297043	0.075805601
STAD	Mast cells resting	0.040858586	0.432650623
STAD	Mast cells activated	-0.102493663	0.048529358
STAD	Eosinophils	-0.15009426	0.003759146
STAD	Neutrophils	-0.170157794	0.001000315
TGCT	B cells naive	0.178112363	0.027615576
TGCT	B cells memory	-0.008387103	0.918047285
TGCT	Plasma cells	-0.02771933	0.733764094
TGCT	T cells CD8	-0.20242553	0.012094801
TGCT	T cells CD4 naive	0	NA
TGCT	T cells CD4 memory resting	0.404570525	2.14E-07
TGCT	T cells CD4 memory activated	0.034127971	0.67536242
TGCT	T cells follicular helper	-0.268960253	0.000774537

TGCT	T cells regulatory (Tregs)	0.218961145	0.00654266
TGCT	T cells gamma delta	0.079117998	0.330983448
TGCT	NK cells resting	-0.129380688	0.110950947
TGCT	NK cells activated	-0.308864573	0.000102529
TGCT	Monocytes	-0.110764825	0.17286754
TGCT	Macrophages M0	0.060404006	0.458265106
TGCT	Macrophages M1	-0.082008634	0.3135646
TGCT	Macrophages M2	-0.153987021	0.057374744
TGCT	Dendritic cells resting	0.082643112	0.309822694
TGCT	Dendritic cells activated	-0.21719528	0.007001003
TGCT	Mast cells resting	0.021409586	0.792796996
TGCT	Mast cells activated	0	NA
TGCT	Eosinophils	-0.251366698	0.001723131
TGCT	Neutrophils	-0.066061877	0.417178563
THCA	B cells naive	-0.048755389	0.334411516
THCA	B cells memory	0.057121143	0.257994635
THCA	Plasma cells	-0.033053828	0.512986855
THCA	T cells CD8	0.149839451	0.002867195
THCA	T cells CD4 naive	0	NA
THCA	T cells CD4 memory resting	-0.113949243	0.023696547
THCA	T cells CD4 memory activated	-0.034255817	0.497773488
THCA	T cells follicular helper	0.016672647	0.741464913
THCA	T cells regulatory (Tregs)	-0.110212754	0.028714975
THCA	T cells gamma delta	0.083007984	0.099914791
THCA	NK cells resting	0.097585222	0.05293158
THCA	NK cells activated	0.057020049	0.258839423
THCA	Monocytes	-0.004342399	0.931529448
THCA	Macrophages M0	-0.047592368	0.346083052
THCA	Macrophages M1	0.095026035	0.059499217
THCA	Macrophages M2	0.079984377	0.112934354
THCA	Dendritic cells resting	-0.033292575	0.509945997
THCA	Dendritic cells activated	-0.12992332	0.009832595
THCA	Mast cells resting	-0.069389909	0.169245688
THCA	Mast cells activated	0.058581992	0.245999407
THCA	Eosinophils	0.080753351	0.109503199
THCA	Neutrophils	-0.129552944	0.010046078
THYM	B cells naive	-0.018299525	0.844074756
THYM	B cells memory	-0.17043519	0.065006057
THYM	Plasma cells	-0.189830927	0.039503728
THYM	T cells CD8	-0.083052481	0.3712561
THYM	T cells CD4 naive	0.146117851	0.114373716
THYM	T cells CD4 memory resting	0.362279786	5.55E-05
THYM	T cells CD4 memory activated	-0.070808506	0.446092055
THYM	T cells follicular helper	-0.035709042	0.701057982
THYM	T cells regulatory (Tregs)	-0.099399429	0.284207795
THYM	T cells gamma delta	-0.172402611	0.061923982
THYM	NK cells resting	0.111308289	0.230147456
THYM	NK cells activated	-0.04619471	0.619381924
THYM	Monocytes	-0.119032214	0.199202852
THYM	Macrophages M0	-0.134448213	0.146631379
THYM	Macrophages M1	-0.036638718	0.693659393
THYM	Macrophages M2	-0.318271364	0.000444245
THYM	Dendritic cells resting	0.1850362	0.044859667
THYM	Dendritic cells activated	-0.03923025	0.673188831
THYM	Mast cells resting	-0.011898259	0.898246456

THYM	Mast cells activated	-0.036938736	0.691277867
THYM	Eosinophils	-0.06028881	0.516646856
THYM	Neutrophils	-0.075790878	0.414666228
UCEC	B cells naive	0.020062132	0.659080236
UCEC	B cells memory	0.046623711	0.305009509
UCEC	Plasma cells	-0.094763411	0.036759949
UCEC	T cells CD8	-0.085420245	0.059874665
UCEC	T cells CD4 naive	0	NA
UCEC	T cells CD4 memory resting	0.088688671	0.050701797
UCEC	T cells CD4 memory activated	-0.064174277	0.157784728
UCEC	T cells follicular helper	0.172505897	0.00013245
UCEC	T cells regulatory (Tregs)	-0.130421866	0.00397536
UCEC	T cells gamma delta	0.099887636	0.027672189
UCEC	NK cells resting	-0.037227114	0.412867746
UCEC	NK cells activated	-0.041422418	0.362182766
UCEC	Monocytes	-0.03912544	0.38943373
UCEC	Macrophages M0	0.015943742	0.725885854
UCEC	Macrophages M1	0.045562396	0.316162171
UCEC	Macrophages M2	-0.03216715	0.479258937
UCEC	Dendritic cells resting	-0.055931864	0.218386731
UCEC	Dendritic cells activated	0.146473361	0.001202732
UCEC	Mast cells resting	0.030956169	0.495968173
UCEC	Mast cells activated	-0.068390642	0.132174929
UCEC	Eosinophils	-0.016272563	0.720466645
UCEC	Neutrophils	-0.054756022	0.228236302
UCS	B cells naive	0.039480296	0.811395957
UCS	B cells memory	-0.165314317	0.314540715
UCS	Plasma cells	-0.254204341	0.118374539
UCS	T cells CD8	0.001247158	0.993987926
UCS	T cells CD4 naive	0	NA
UCS	T cells CD4 memory resting	-0.015552012	0.925134626
UCS	T cells CD4 memory activated	-0.01398974	0.932637083
UCS	T cells follicular helper	-0.057311038	0.728931162
UCS	T cells regulatory (Tregs)	-0.007669027	0.96304288
UCS	T cells gamma delta	-0.021264093	0.897762724
UCS	NK cells resting	0.124378214	0.450605057
UCS	NK cells activated	0.20941372	0.200727766
UCS	Monocytes	0.142951333	0.385308731
UCS	Macrophages M0	0.037173521	0.822233639
UCS	Macrophages M1	-0.052738892	0.749832242
UCS	Macrophages M2	0.135301065	0.411503745
UCS	Dendritic cells resting	-0.248115748	0.127759603
UCS	Dendritic cells activated	0.030632715	0.853134548
UCS	Mast cells resting	-0.146696268	0.372850743
UCS	Mast cells activated	-0.219422027	0.1795538
UCS	Eosinophils	-0.043314974	0.79345726
UCS	Neutrophils	0.012351389	0.940511051
UVM	B cells naive	0.040243565	0.810417083
UVM	B cells memory	0.118821233	0.477382682
UVM	Plasma cells	-0.123679885	0.459425617
UVM	T cells CD8	0.179690283	0.280365116
UVM	T cells CD4 naive	0	NA
UVM	T cells CD4 memory resting	0.008730789	0.958510166
UVM	T cells CD4 memory activated	0.351317088	0.03055743
UVM	T cells follicular helper	0.39371551	0.014459685

UVM	T cells regulatory (Tregs)	-0.006487253	0.969165776
UVM	T cells gamma delta	0.093814632	0.575321446
UVM	NK cells resting	0.080871848	0.629332846
UVM	NK cells activated	-0.188125725	0.258021199
UVM	Monocytes	-0.397974671	0.013342258
UVM	Macrophages M0	0.092255775	0.581714359
UVM	Macrophages M1	0.294079495	0.073111639
UVM	Macrophages M2	-0.341518439	0.03586174
UVM	Dendritic cells resting	0.007687551	0.963464223
UVM	Dendritic cells activated	0	NA
UVM	Mast cells resting	-0.228342982	0.16793312
UVM	Mast cells activated	0.257538154	0.118523395
UVM	Eosinophils	-0.151948682	0.362459541
UVM	Neutrophils	0.300457884	0.066819887
